# Supplementary figures and images for: Corneal biomechanical cues mediated by PAI-2: the origin of PM2.5-induced corneal disease (part 1 of 2)
Source: EMBO Mol Med. 2025 Dec 1;18(1):120–50. doi: 10.1038/s44321-025-00341-0 (PMC12808792; doi:10.1038/s44321-025-00341-0)

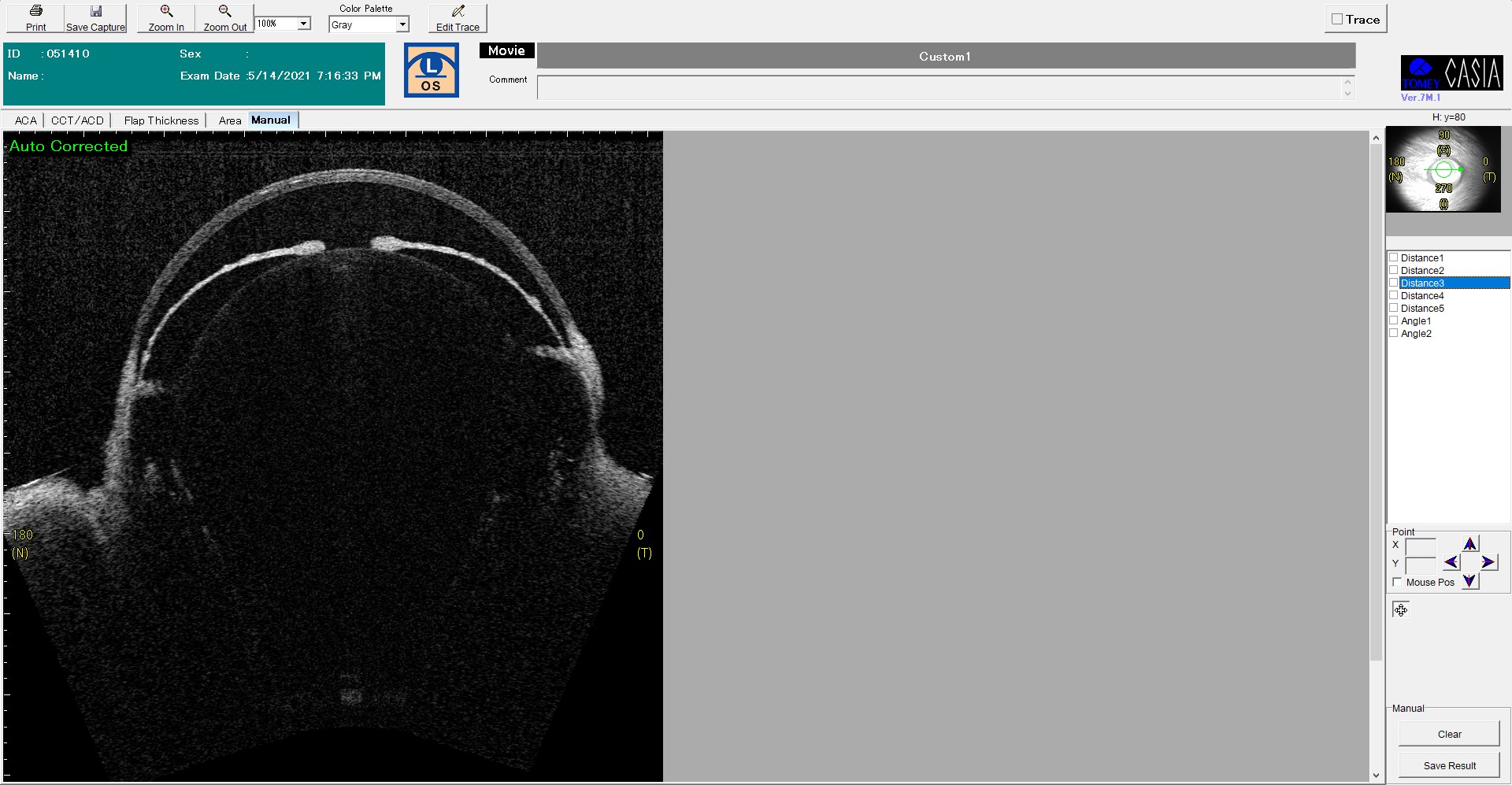

Supplement: Supplementary file 7 — Source data Fig. 1 [file 44321_2025_341_MOESM7_ESM.zip › Figure 1/1E/Rat corneal photo by AS-OCT_PM 0 day.jpg]

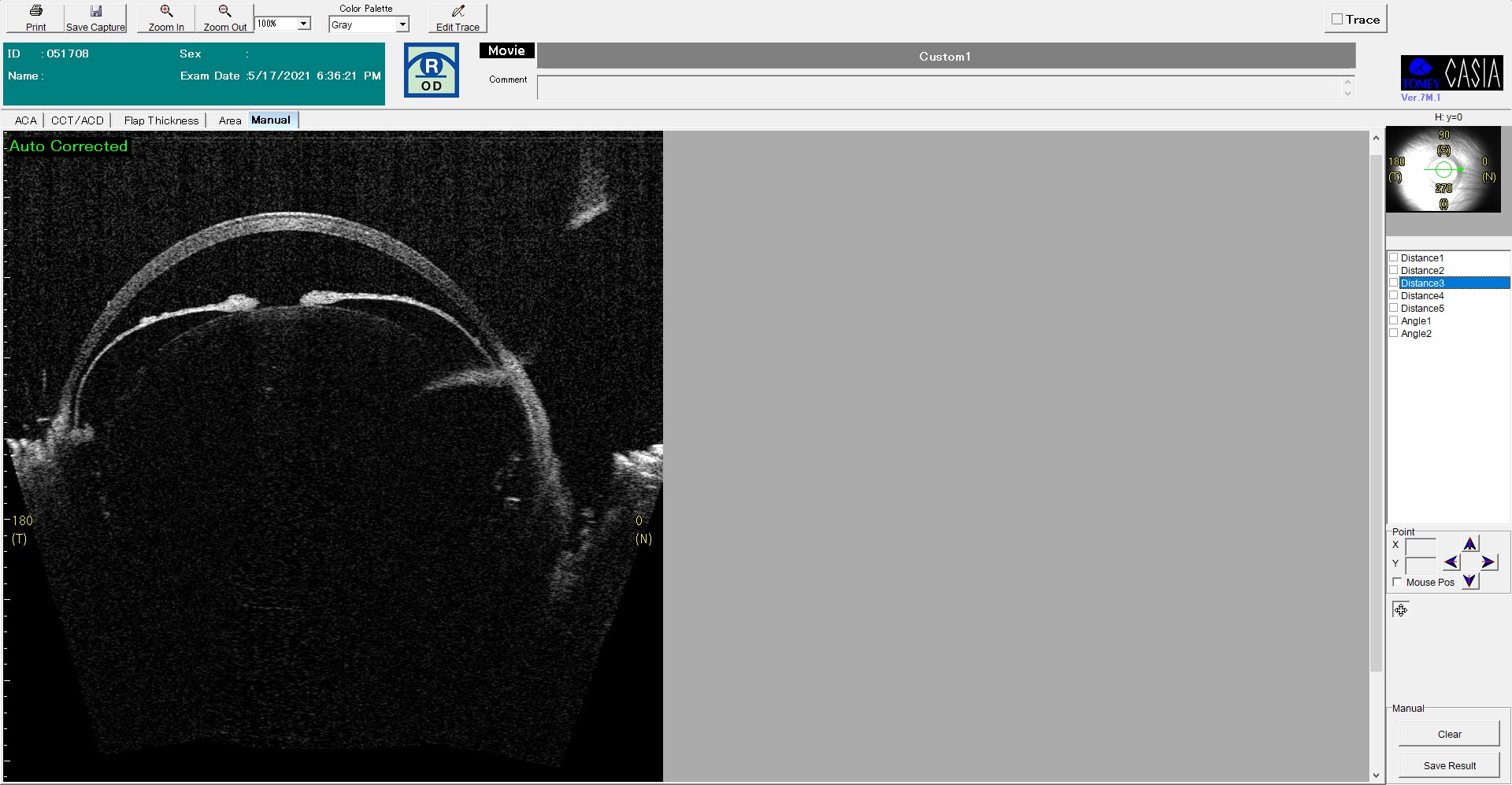

Supplement: Supplementary file 7 — Source data Fig. 1 [file 44321_2025_341_MOESM7_ESM.zip › Figure 1/1E/Rat corneal photo by AS-OCT_PM 2 days.jpg]

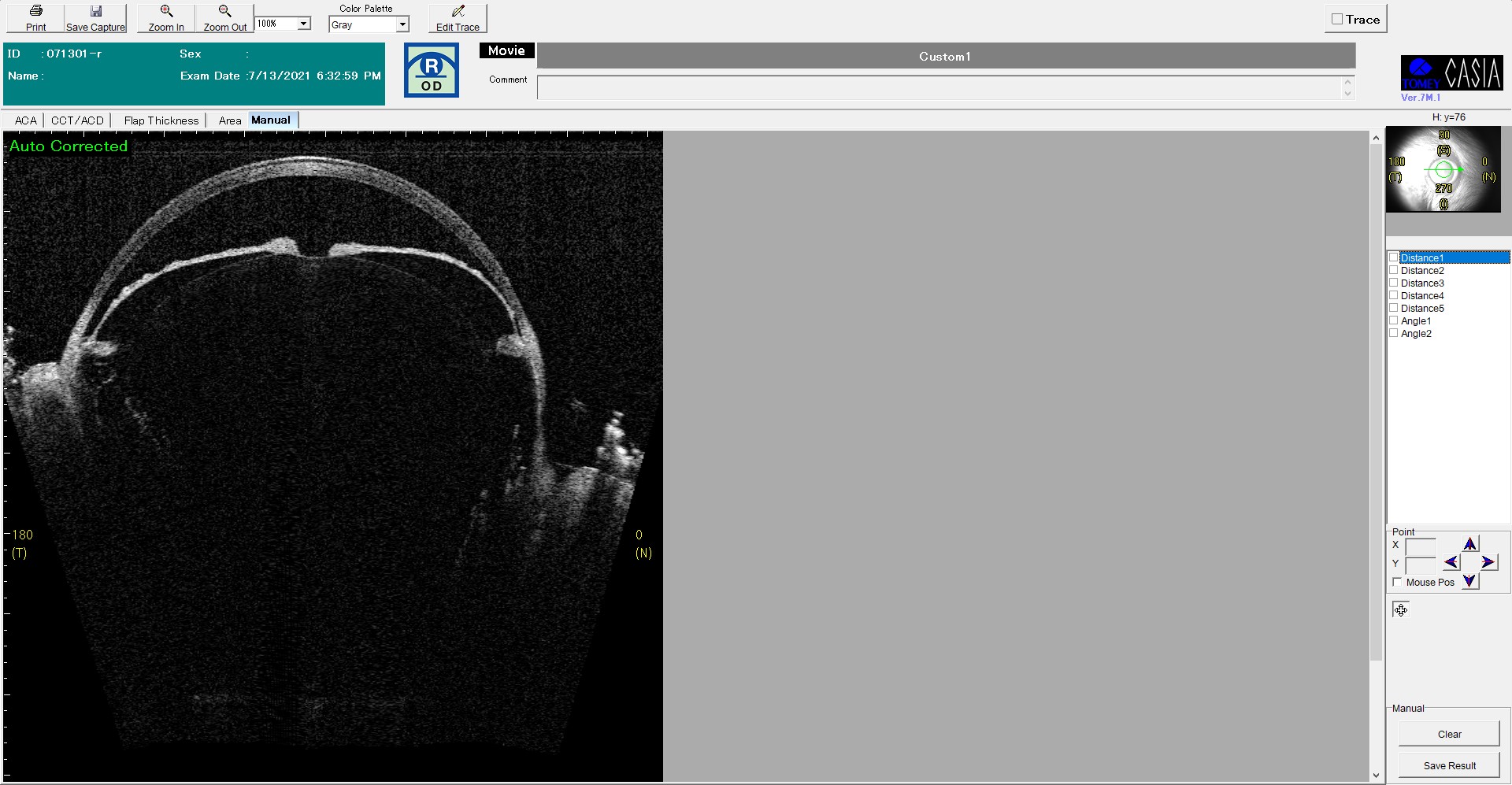

Supplement: Supplementary file 7 — Source data Fig. 1 [file 44321_2025_341_MOESM7_ESM.zip › Figure 1/1E/Rat corneal photo by AS-OCT_PBS 3 weeks.jpg]

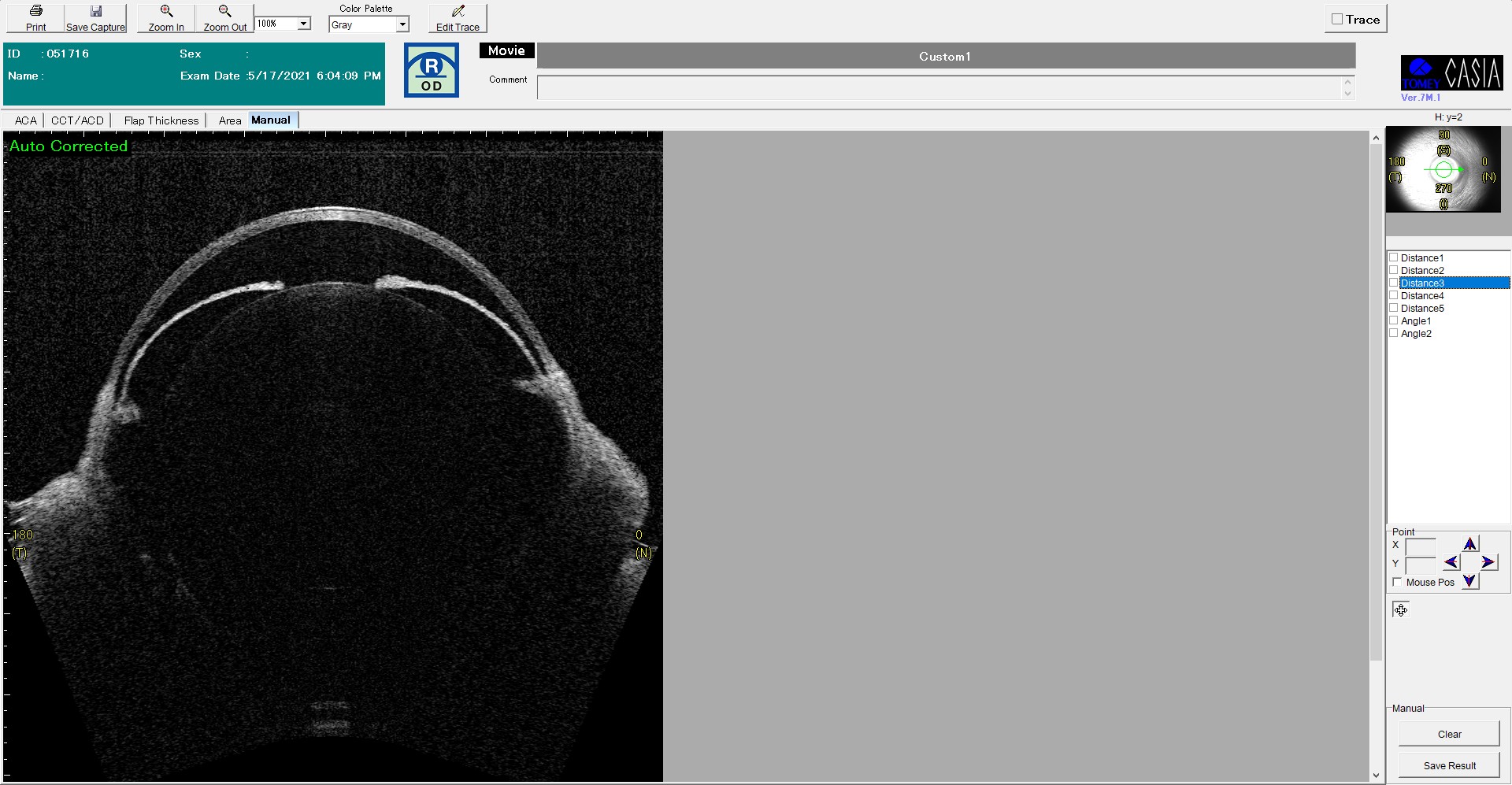

Supplement: Supplementary file 7 — Source data Fig. 1 [file 44321_2025_341_MOESM7_ESM.zip › Figure 1/1E/Rat corneal photo by AS-OCT_PBS 2 days.jpg]

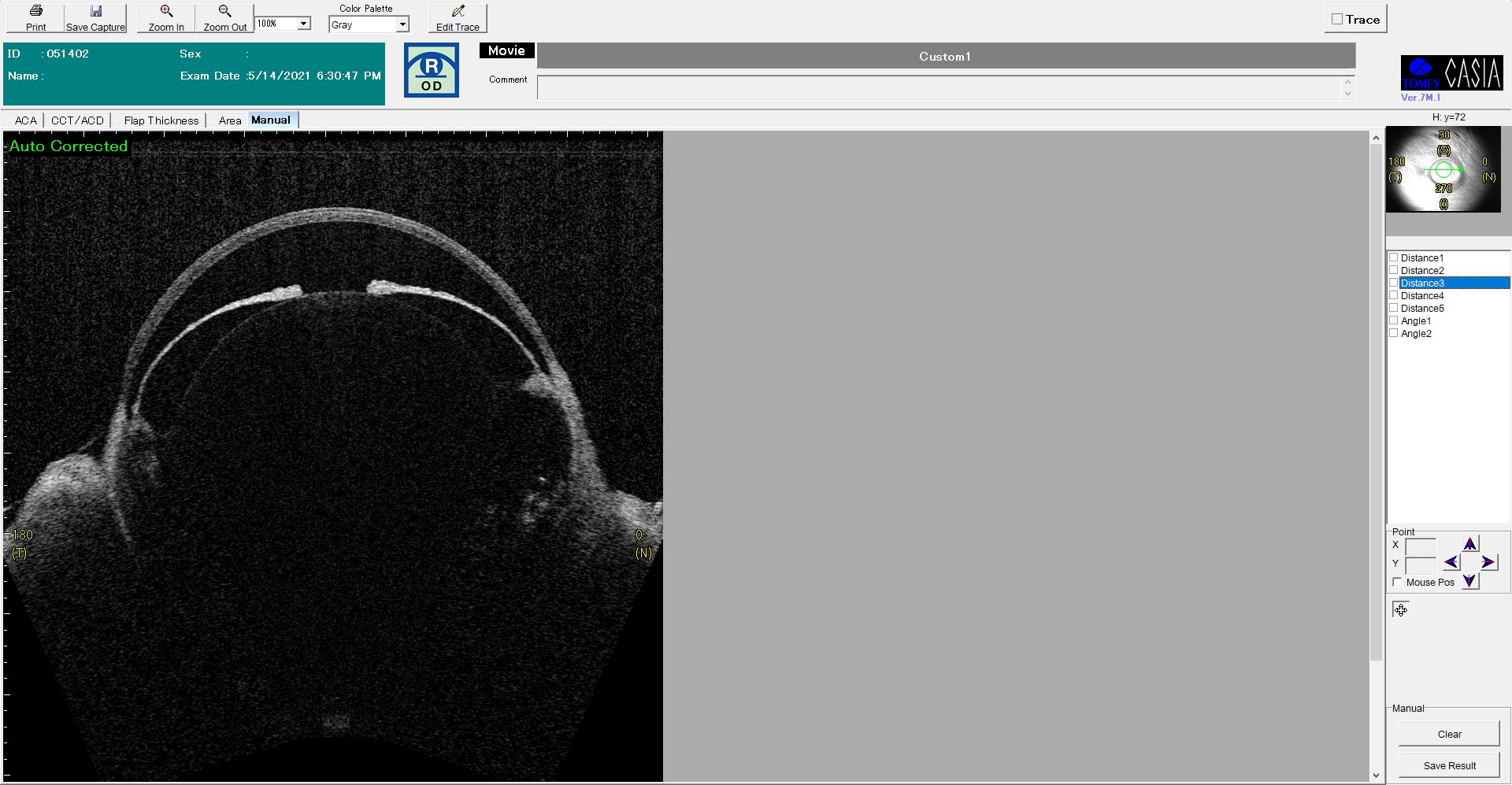

Supplement: Supplementary file 7 — Source data Fig. 1 [file 44321_2025_341_MOESM7_ESM.zip › Figure 1/1E/Rat corneal photo by AS-OCT_PBS 0 day.jpg]

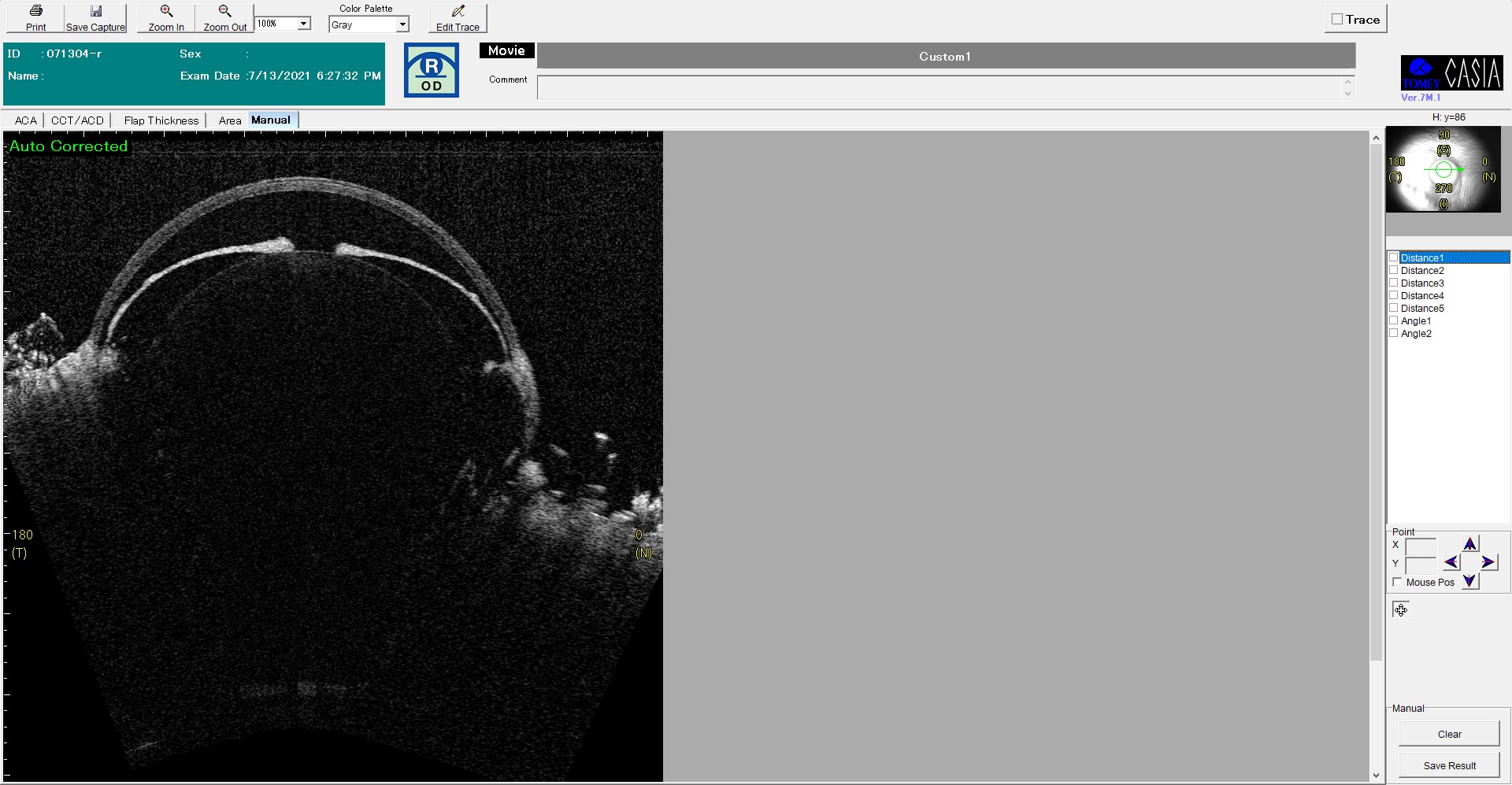

Supplement: Supplementary file 7 — Source data Fig. 1 [file 44321_2025_341_MOESM7_ESM.zip › Figure 1/1E/Rat corneal photo by AS-OCT_PM 3 weeks.jpg]

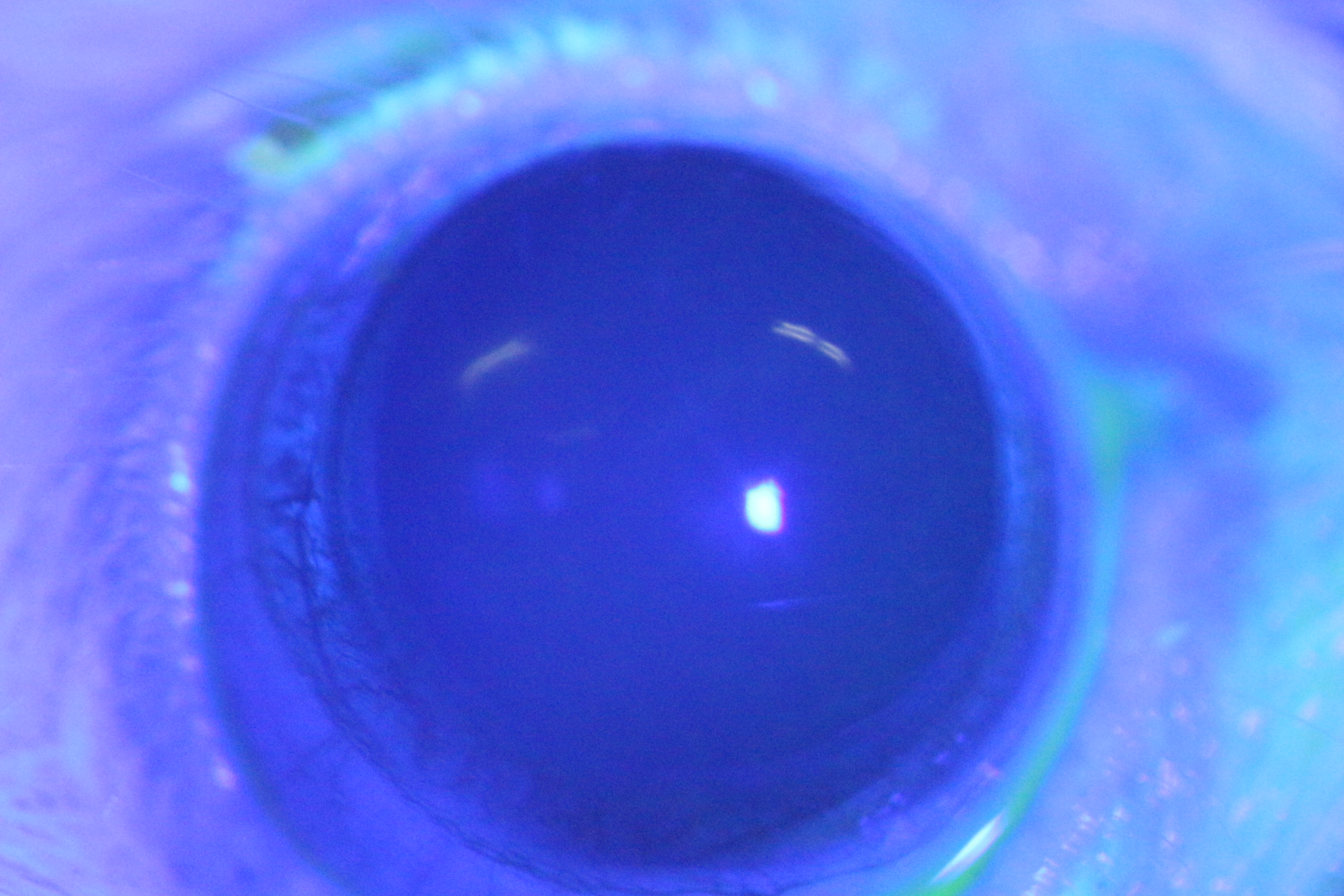

Supplement: Supplementary file 7 — Source data Fig. 1 [file 44321_2025_341_MOESM7_ESM.zip › Figure 1/1D/Rat corneal photo by slit lamp_PM 0 day.JPG]

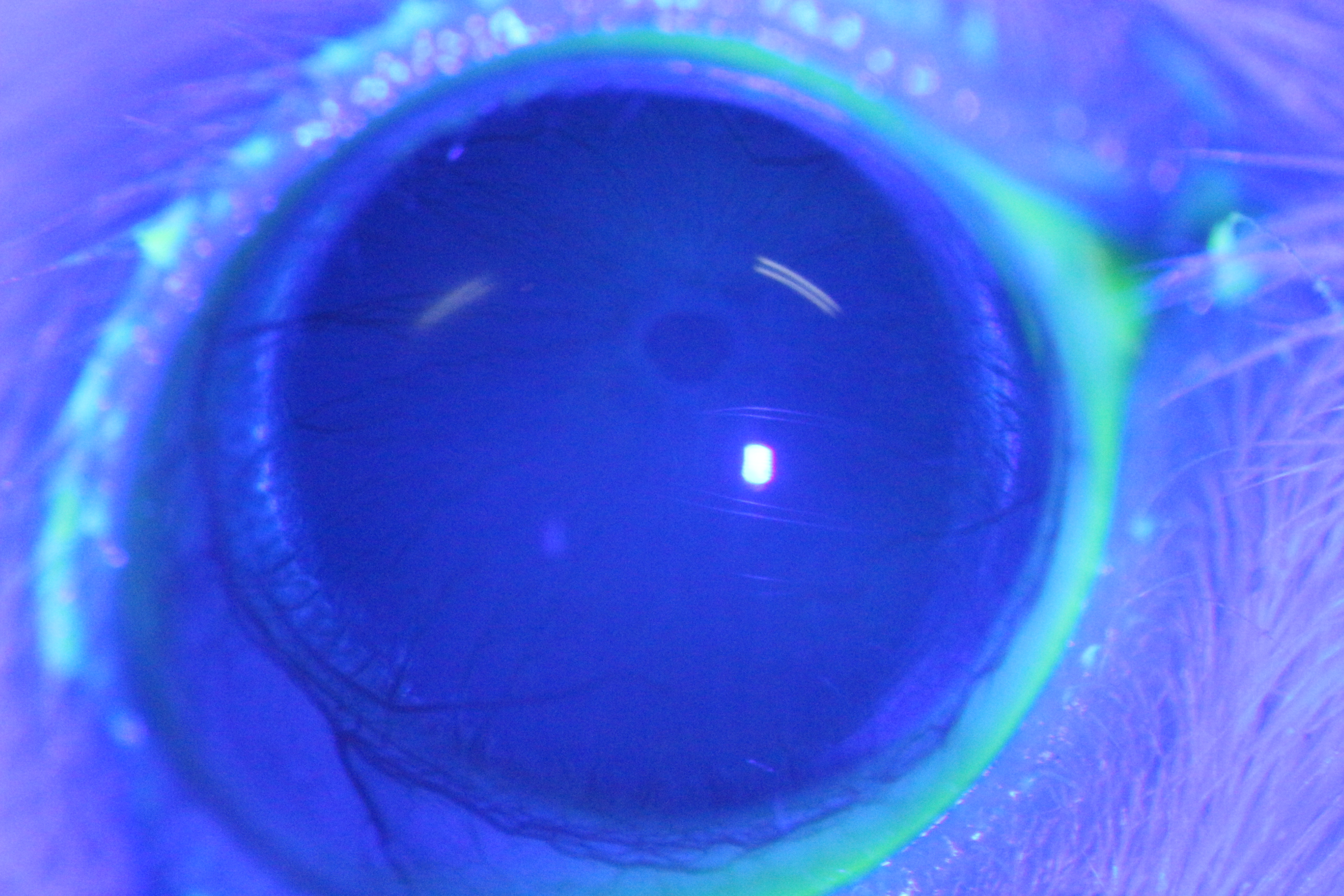

Supplement: Supplementary file 7 — Source data Fig. 1 [file 44321_2025_341_MOESM7_ESM.zip › Figure 1/1D/Rat corneal photo by slit lamp_PBS 2 days.JPG]

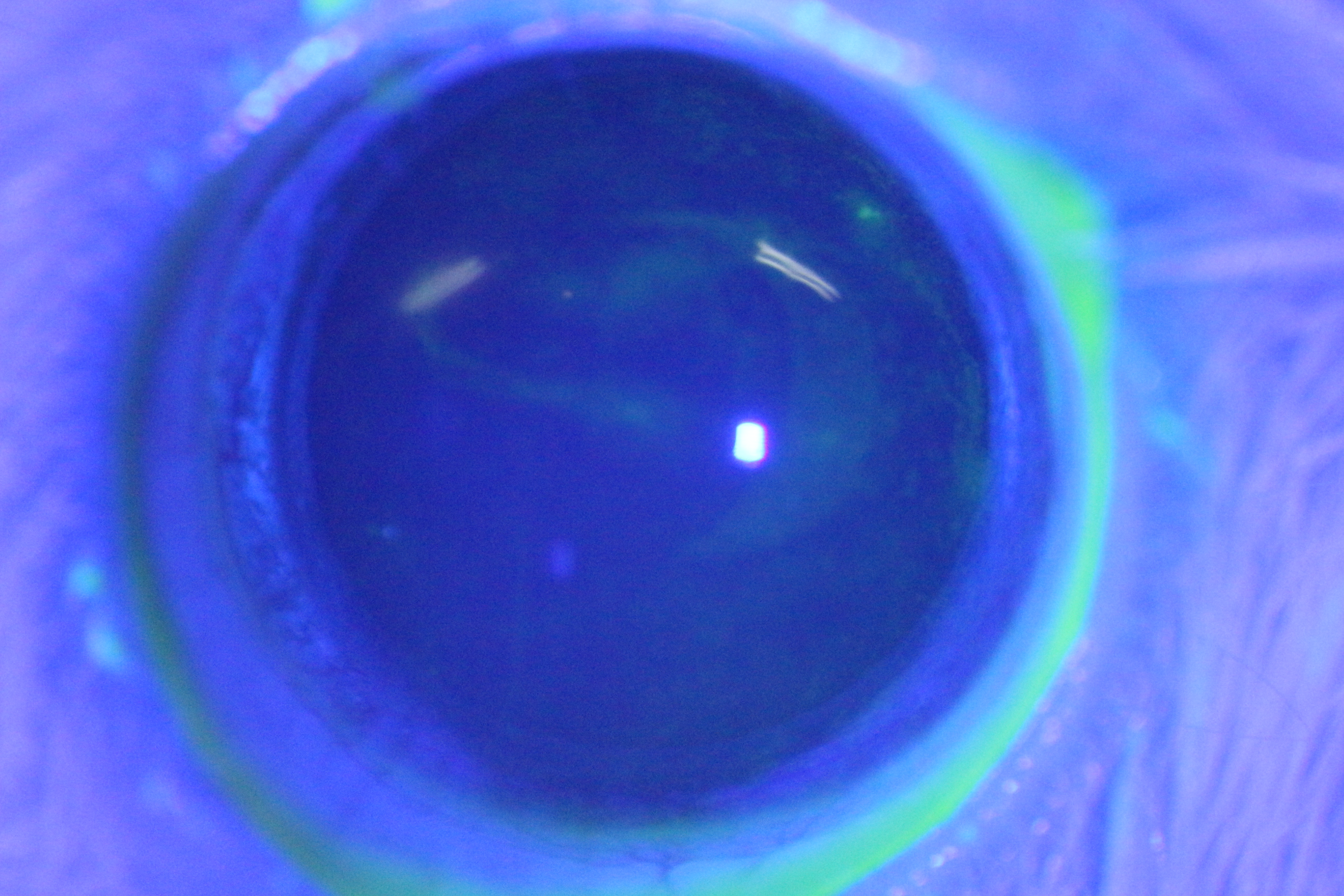

Supplement: Supplementary file 7 — Source data Fig. 1 [file 44321_2025_341_MOESM7_ESM.zip › Figure 1/1D/Rat corneal photo by slit lamp_PM 3 weeks.JPG]

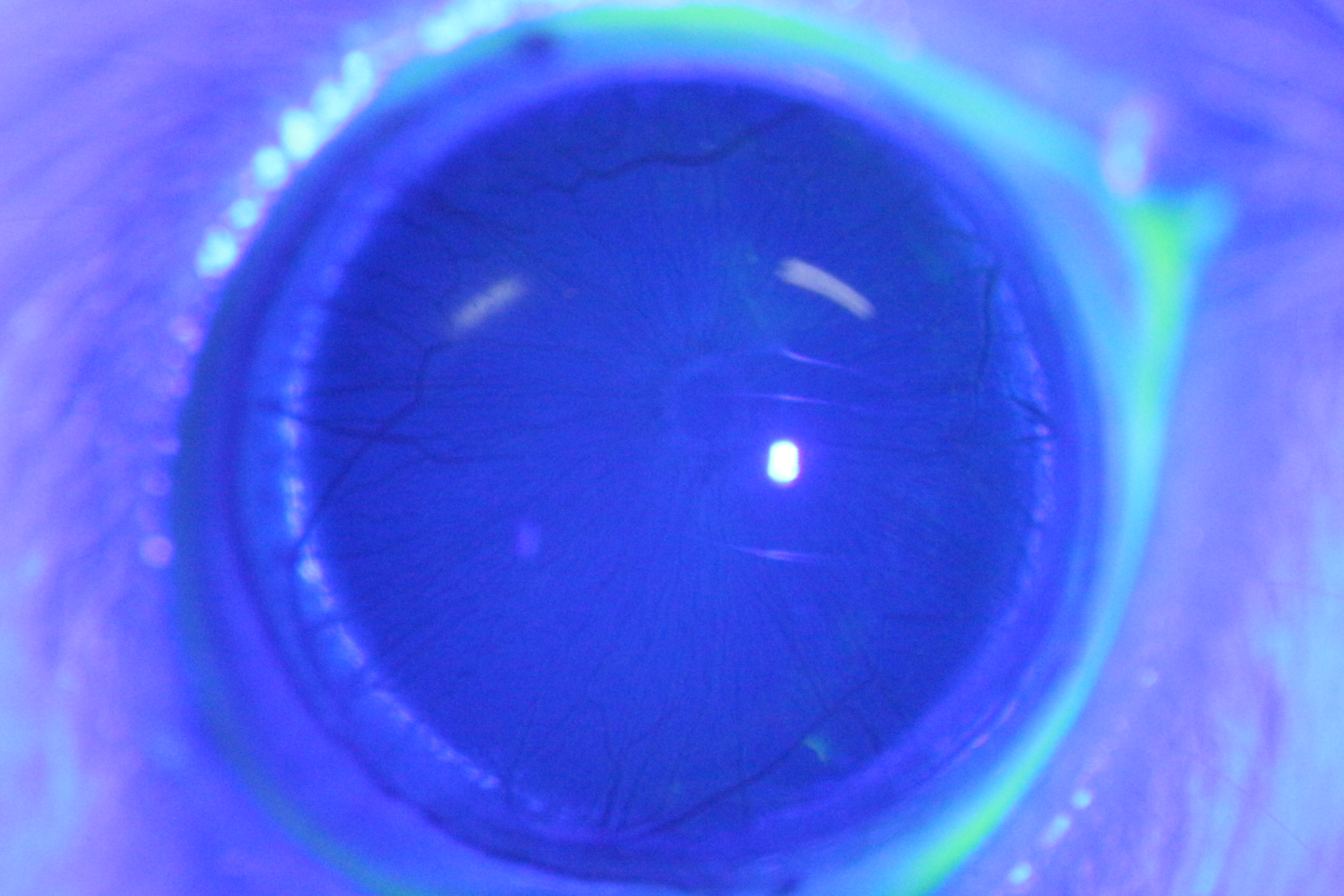

Supplement: Supplementary file 7 — Source data Fig. 1 [file 44321_2025_341_MOESM7_ESM.zip › Figure 1/1D/Rat corneal photo by slit lamp_PM 2 days.JPG]

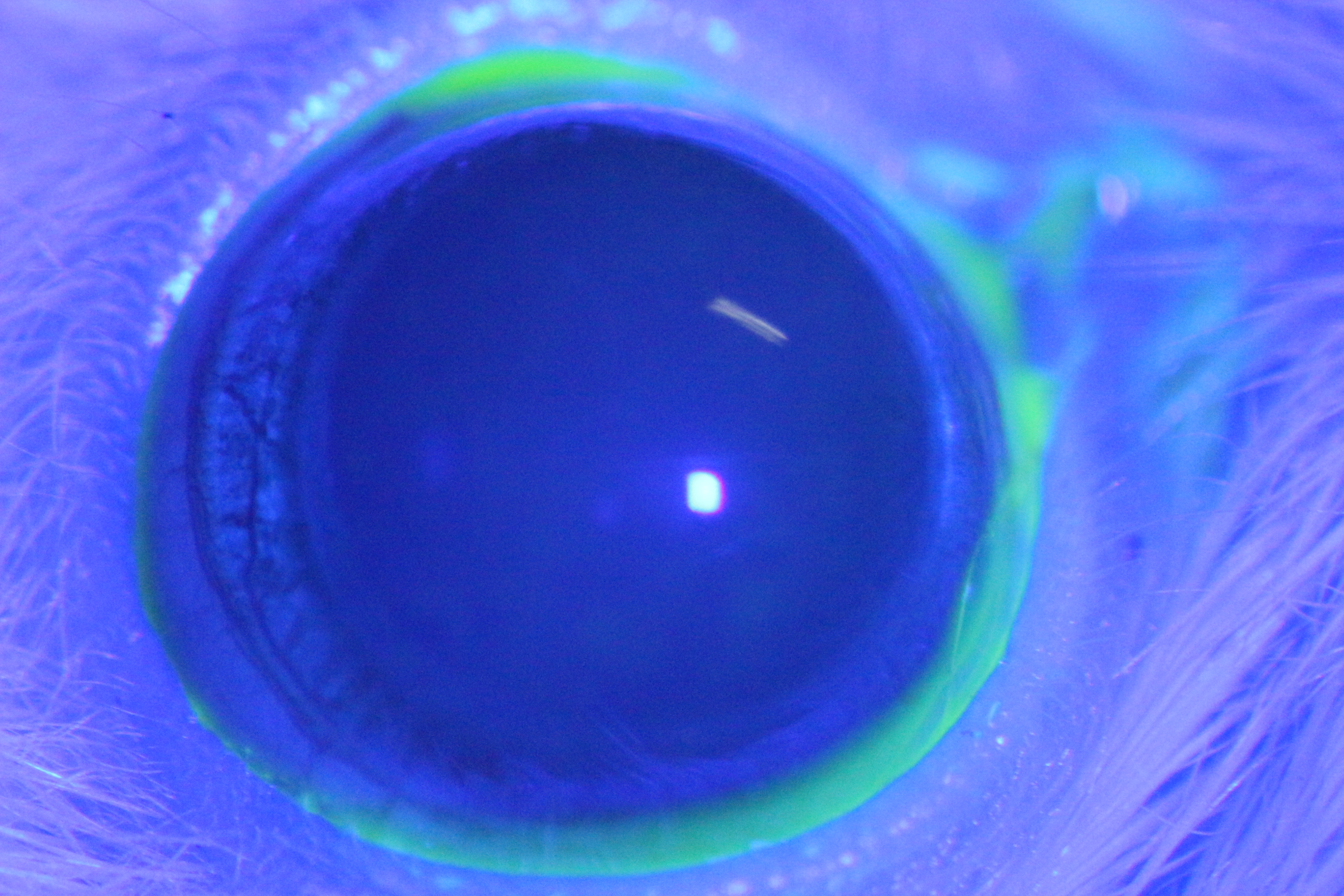

Supplement: Supplementary file 7 — Source data Fig. 1 [file 44321_2025_341_MOESM7_ESM.zip › Figure 1/1D/Rat corneal photo by slit lamp_PBS 0 day.JPG]

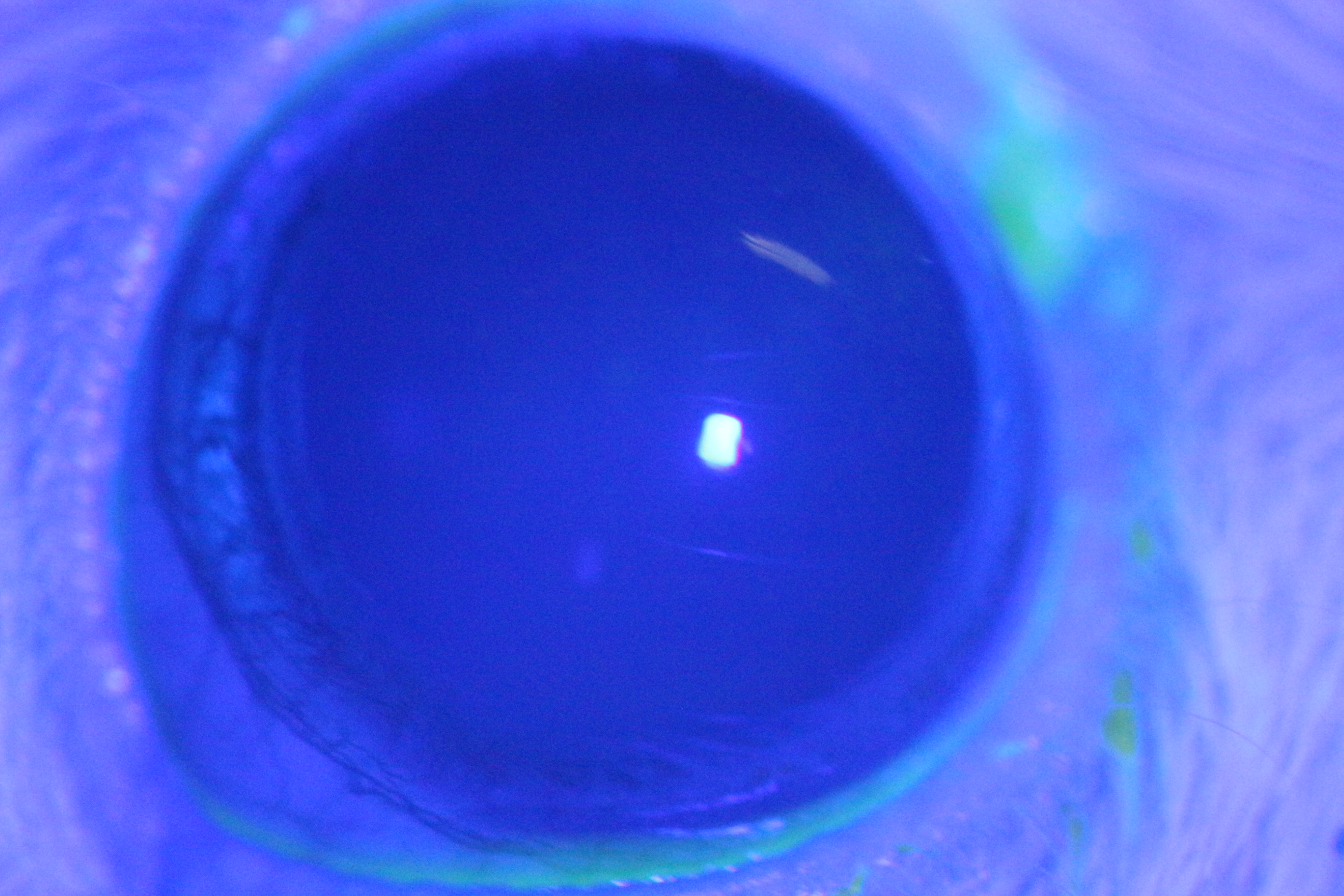

Supplement: Supplementary file 7 — Source data Fig. 1 [file 44321_2025_341_MOESM7_ESM.zip › Figure 1/1D/Rat corneal photo by slit lamp_PBS 3 weeks.JPG]

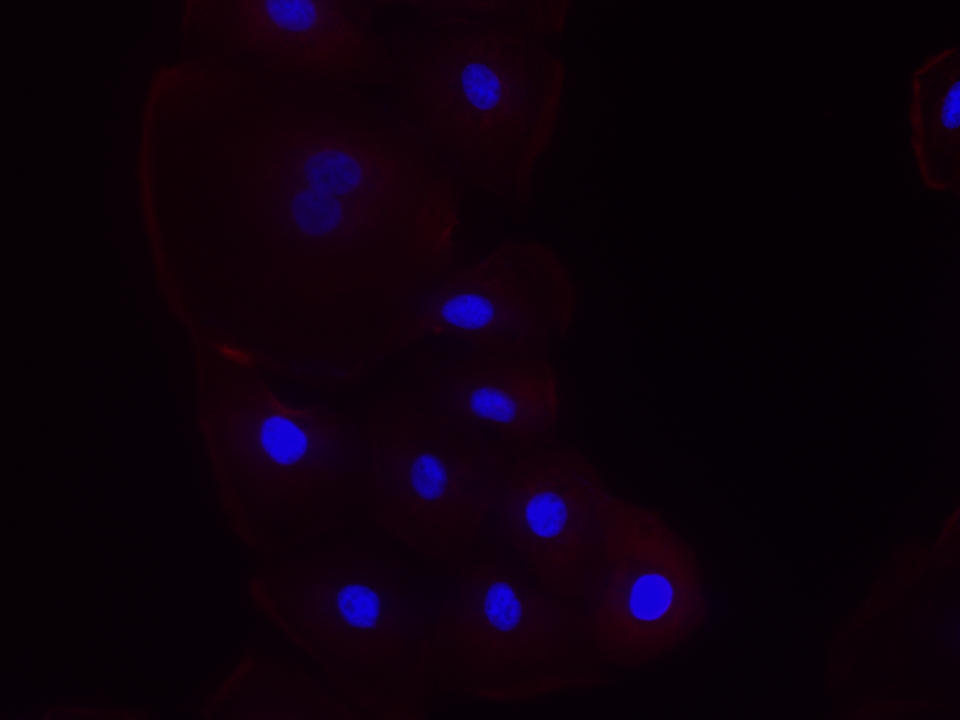

Supplement: Supplementary file 8 — Source data Fig. 2 [file 44321_2025_341_MOESM8_ESM.zip › Figure 2/2A/Immunofluorescence_0h merge.tif]

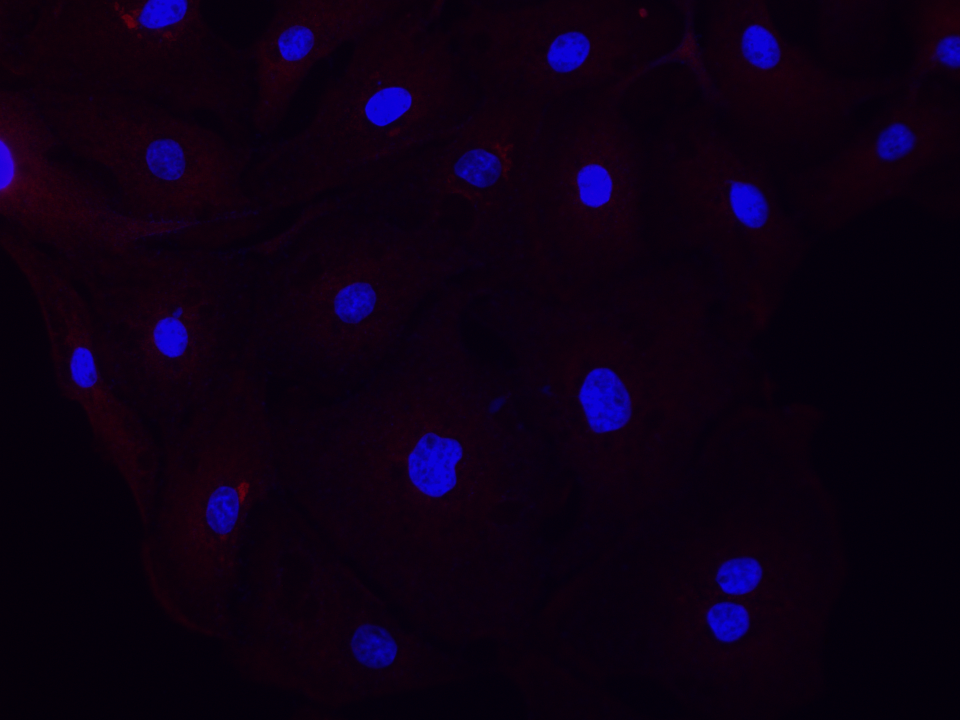

Supplement: Supplementary file 8 — Source data Fig. 2 [file 44321_2025_341_MOESM8_ESM.zip › Figure 2/2A/Immunofluorescence_24h merge.tif]

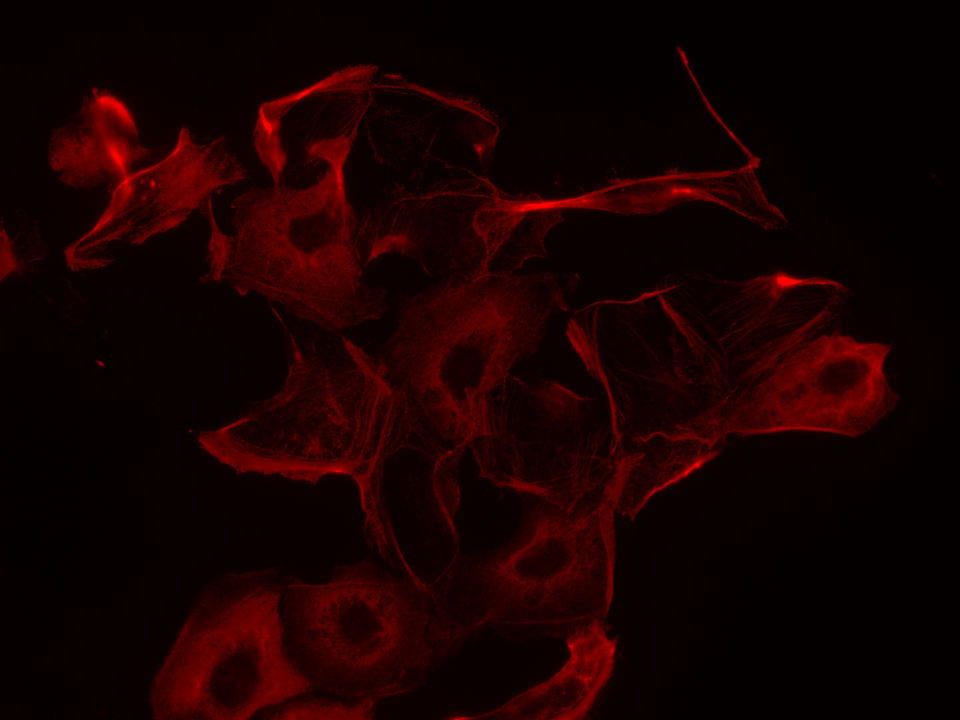

Supplement: Supplementary file 8 — Source data Fig. 2 [file 44321_2025_341_MOESM8_ESM.zip › Figure 2/2A/Immunofluorescence_1h p-myosin IIa.tif]

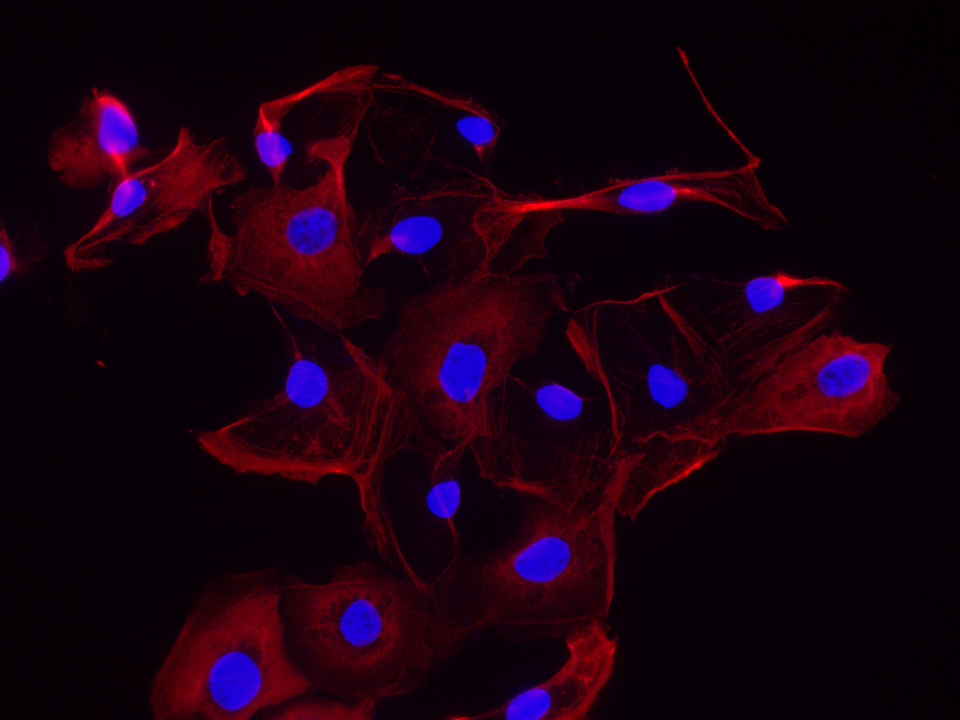

Supplement: Supplementary file 8 — Source data Fig. 2 [file 44321_2025_341_MOESM8_ESM.zip › Figure 2/2A/Immunofluorescence_1h merge.tif]

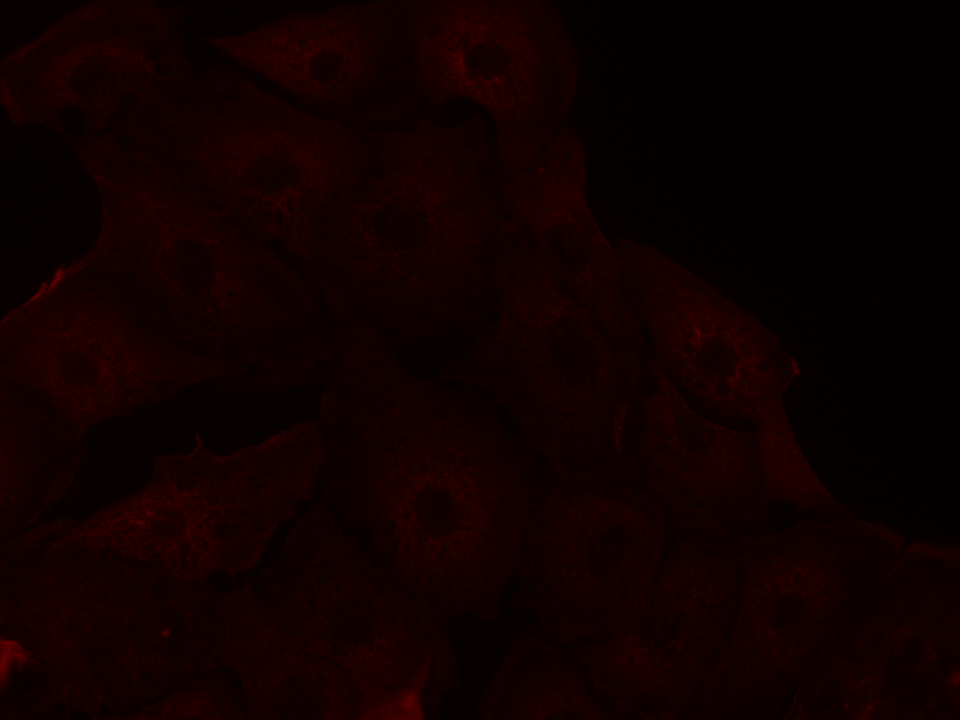

Supplement: Supplementary file 8 — Source data Fig. 2 [file 44321_2025_341_MOESM8_ESM.zip › Figure 2/2A/Immunofluorescence_12h p-myosin IIa.tif]

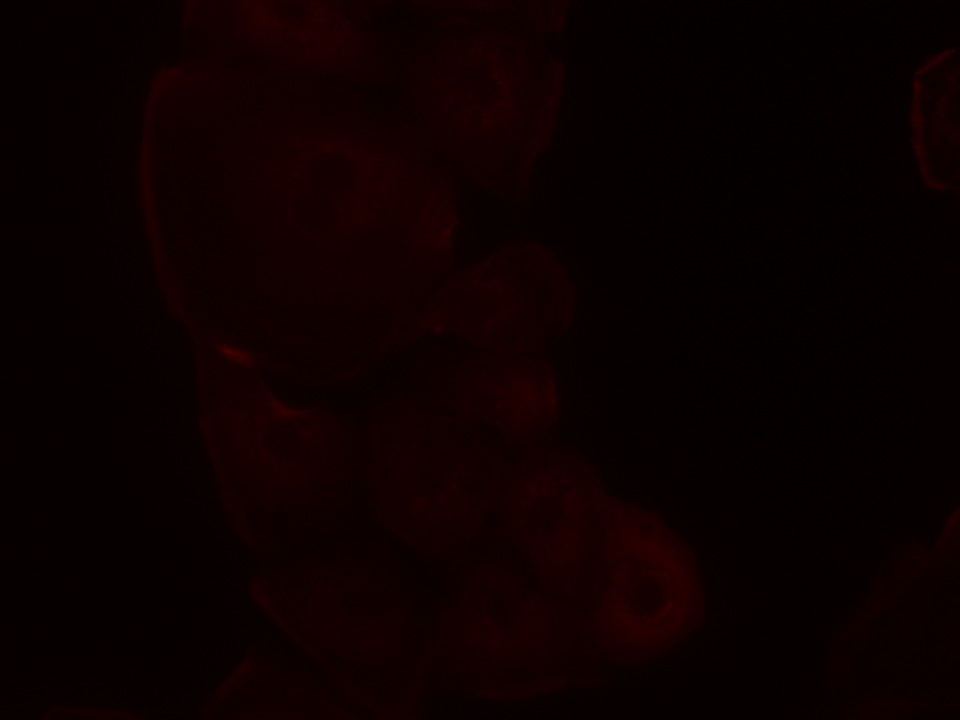

Supplement: Supplementary file 8 — Source data Fig. 2 [file 44321_2025_341_MOESM8_ESM.zip › Figure 2/2A/Immunofluorescence_0h p-myosin IIa.tif]

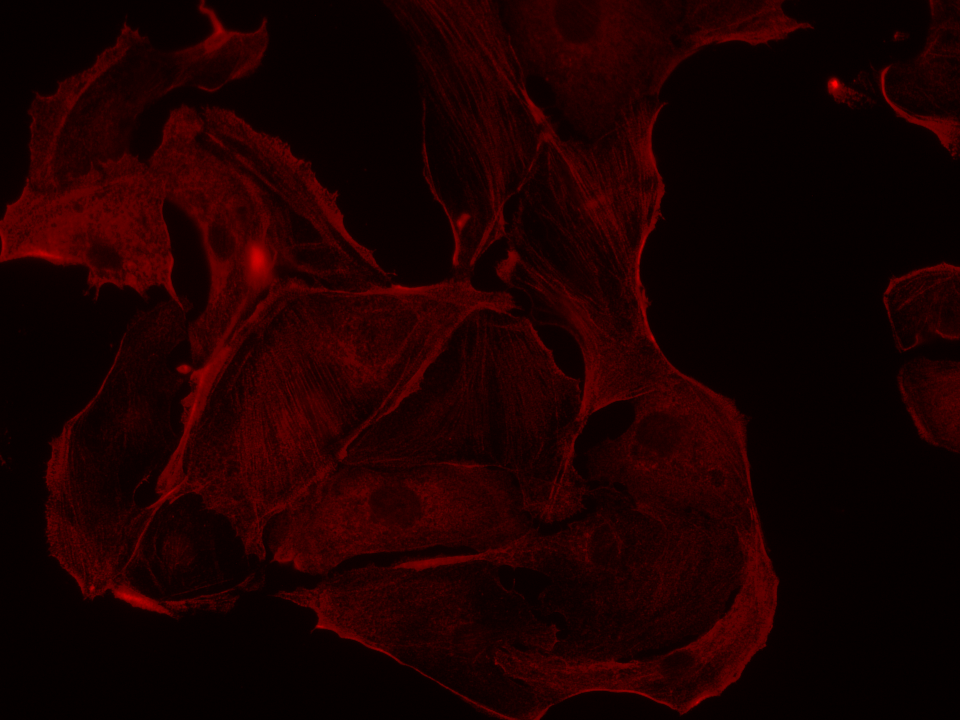

Supplement: Supplementary file 8 — Source data Fig. 2 [file 44321_2025_341_MOESM8_ESM.zip › Figure 2/2A/Immunofluorescence_6h p-myosin IIa.tif]

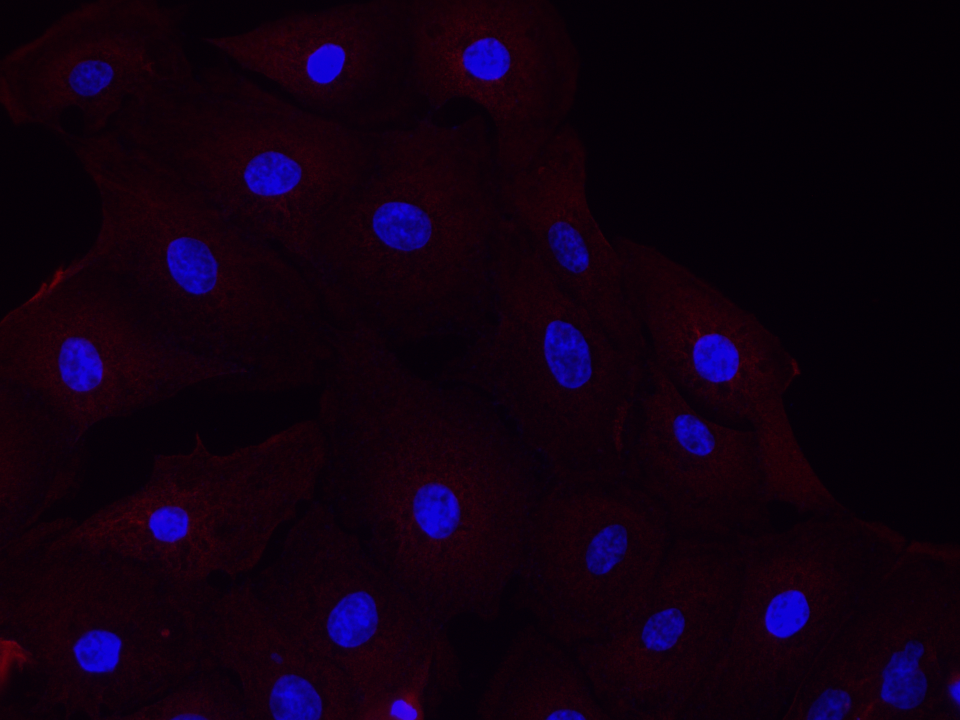

Supplement: Supplementary file 8 — Source data Fig. 2 [file 44321_2025_341_MOESM8_ESM.zip › Figure 2/2A/Immunofluorescence_12h merge.tif]

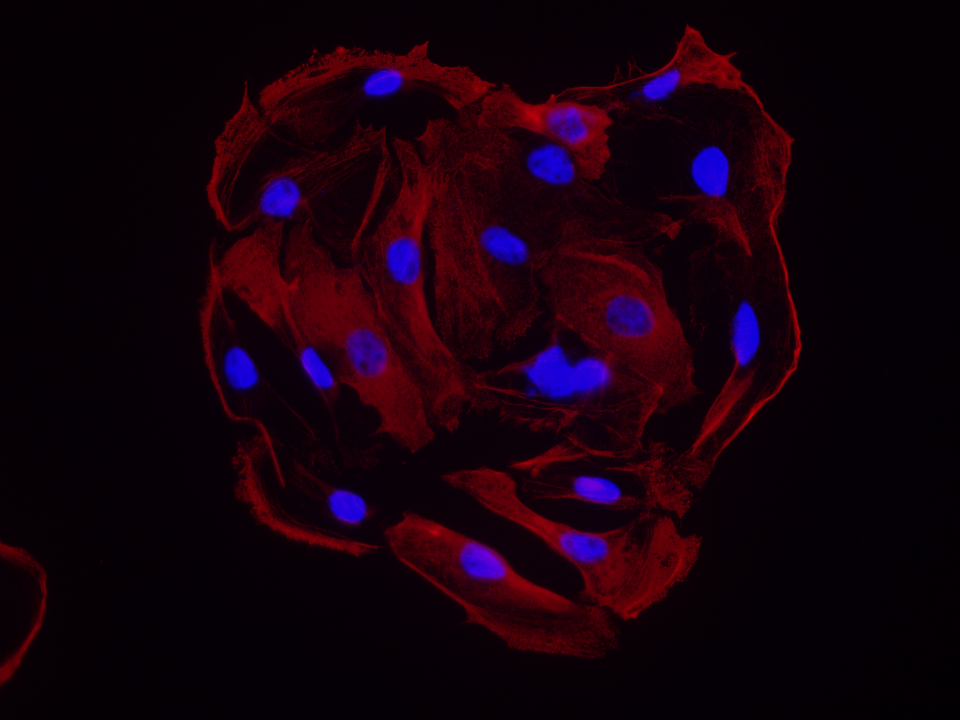

Supplement: Supplementary file 8 — Source data Fig. 2 [file 44321_2025_341_MOESM8_ESM.zip › Figure 2/2A/Immunofluorescence_3h merge.tif]

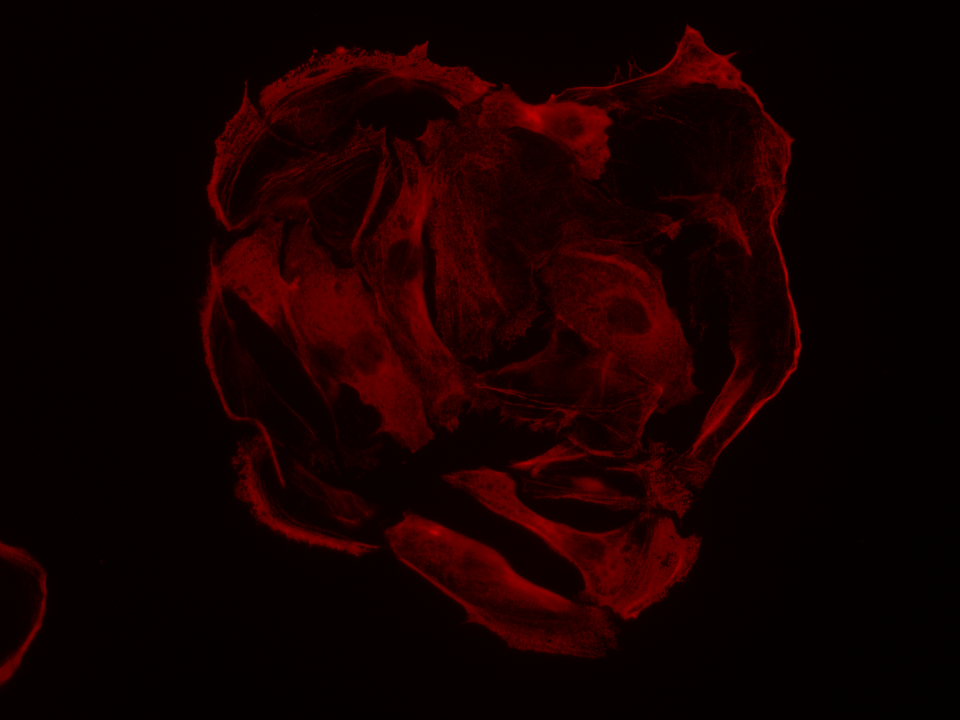

Supplement: Supplementary file 8 — Source data Fig. 2 [file 44321_2025_341_MOESM8_ESM.zip › Figure 2/2A/Immunofluorescence_3h p-myosin IIa.tif]

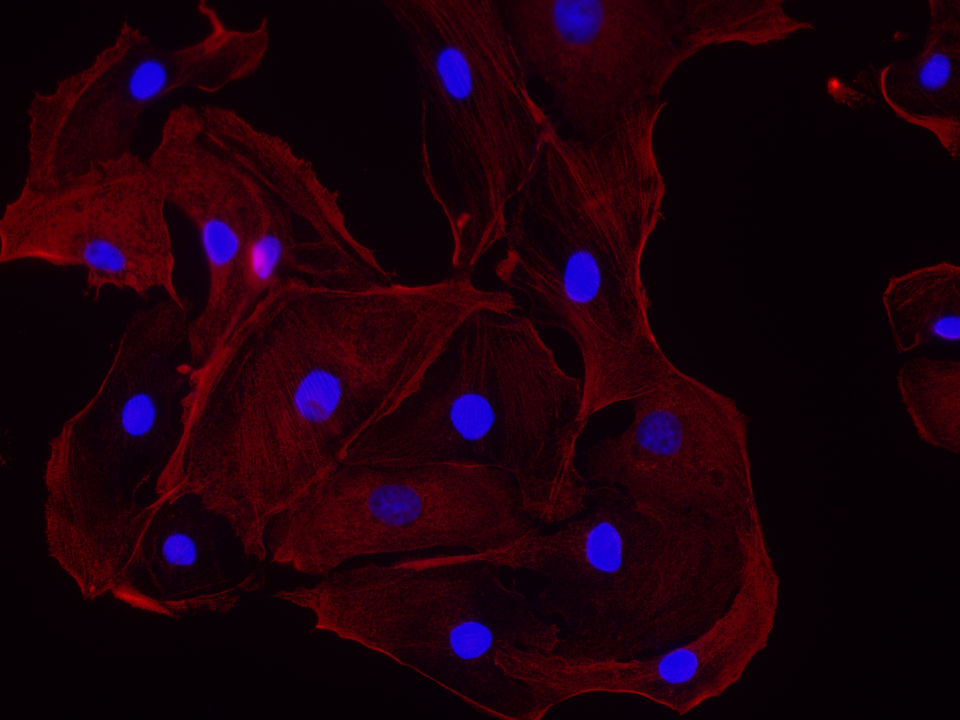

Supplement: Supplementary file 8 — Source data Fig. 2 [file 44321_2025_341_MOESM8_ESM.zip › Figure 2/2A/Immunofluorescence_6h merge.tif]

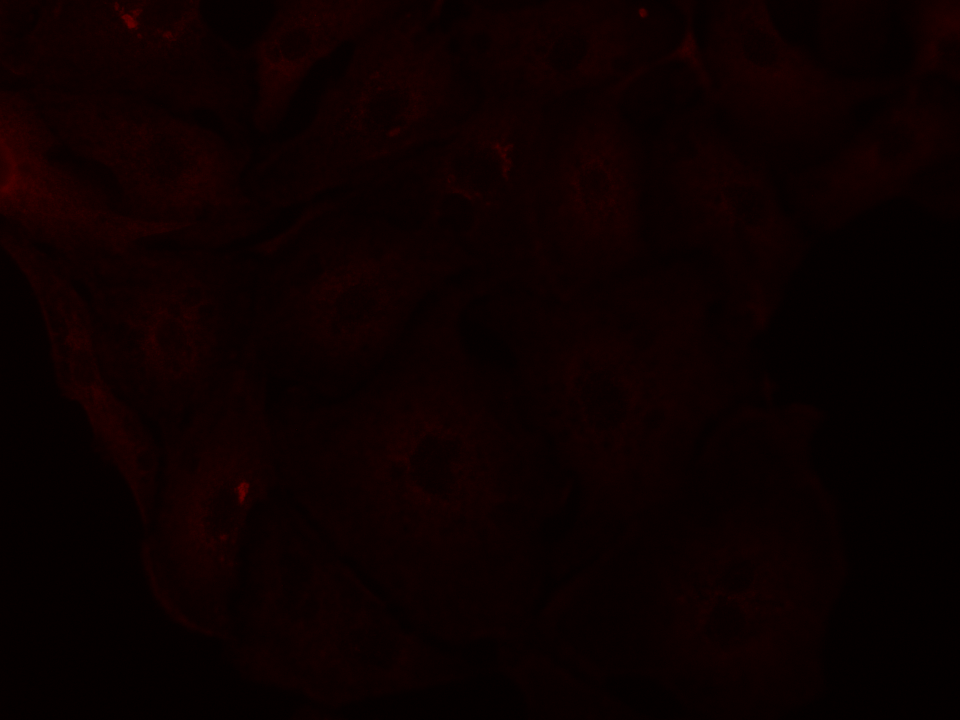

Supplement: Supplementary file 8 — Source data Fig. 2 [file 44321_2025_341_MOESM8_ESM.zip › Figure 2/2A/Immunofluorescence_24h p-myosin IIa.tif]

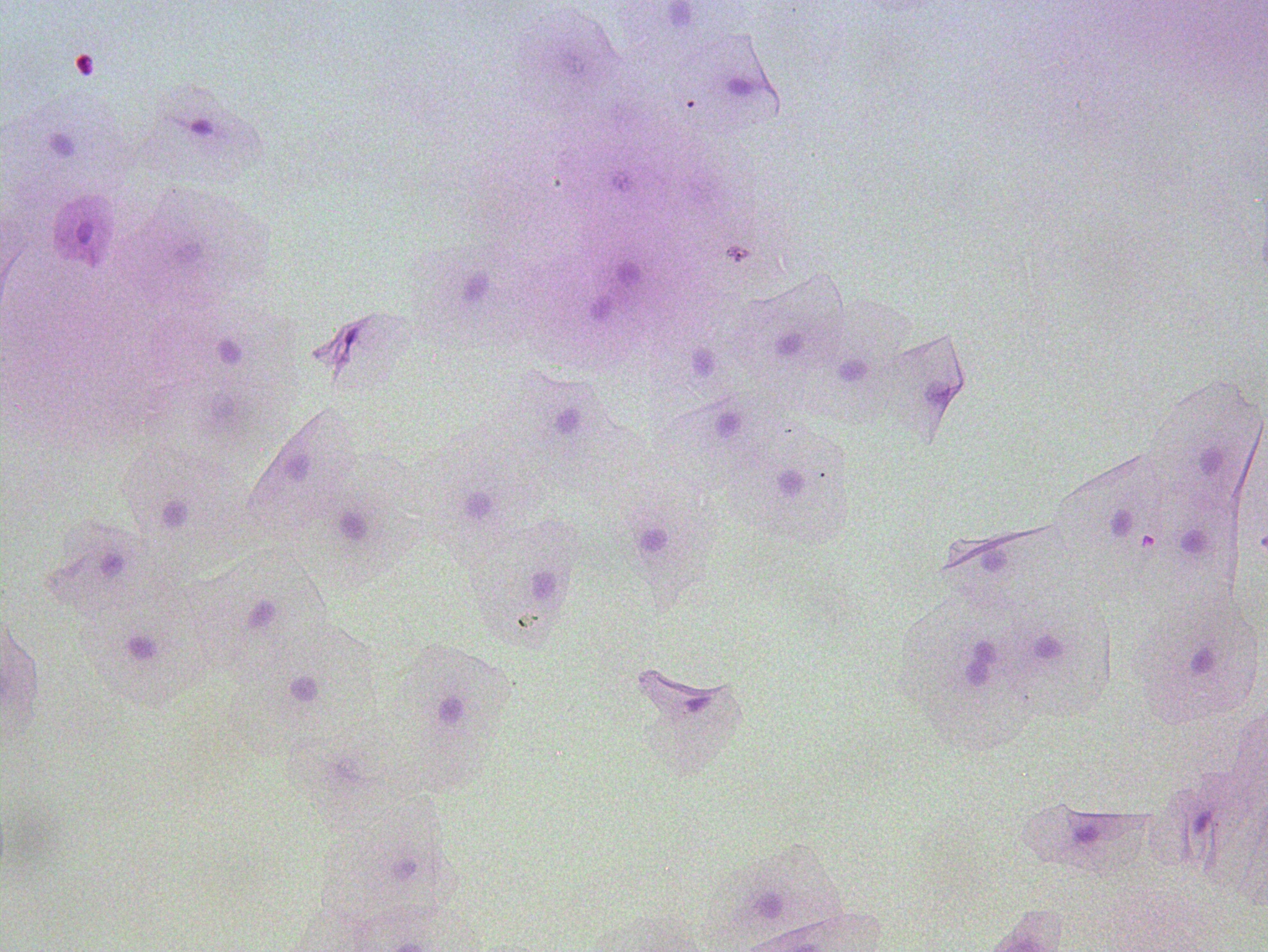

Supplement: Supplementary file 8 — Source data Fig. 2 [file 44321_2025_341_MOESM8_ESM.zip › Figure 2/2E/Impression cytology_PBS.tif]

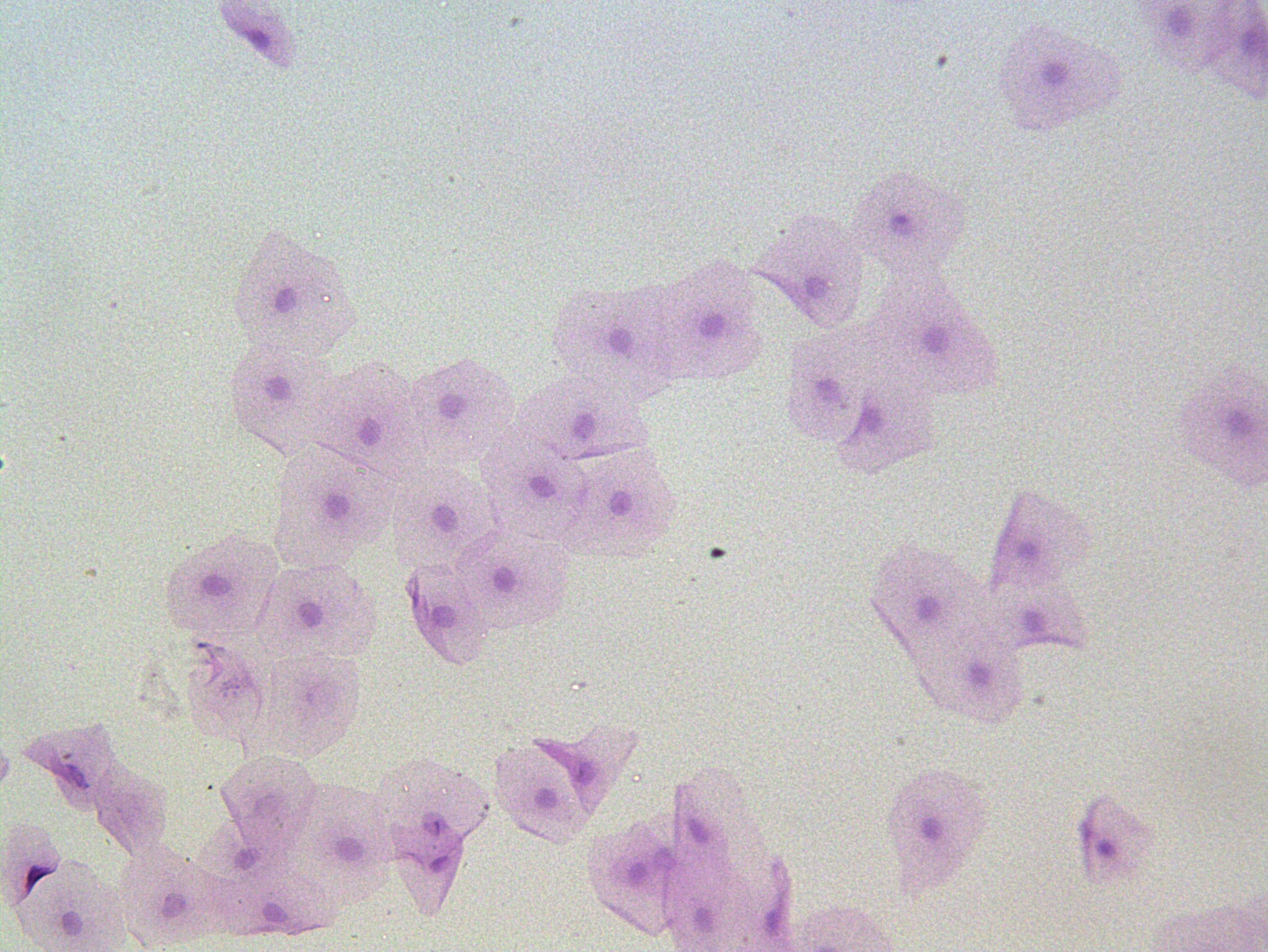

Supplement: Supplementary file 8 — Source data Fig. 2 [file 44321_2025_341_MOESM8_ESM.zip › Figure 2/2E/Impression cytology_PM.tif]

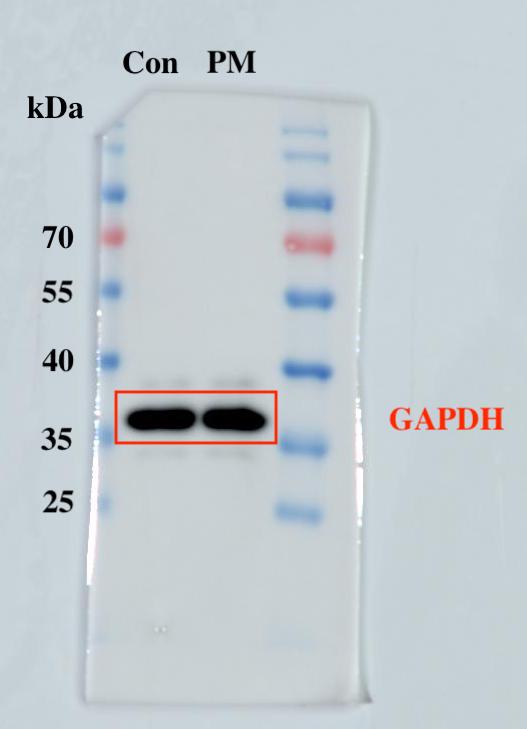

Supplement: Supplementary file 9 — Source data Fig. 3 [file 44321_2025_341_MOESM9_ESM.zip › Figure 3/3C/WB GAPDH.jpg]

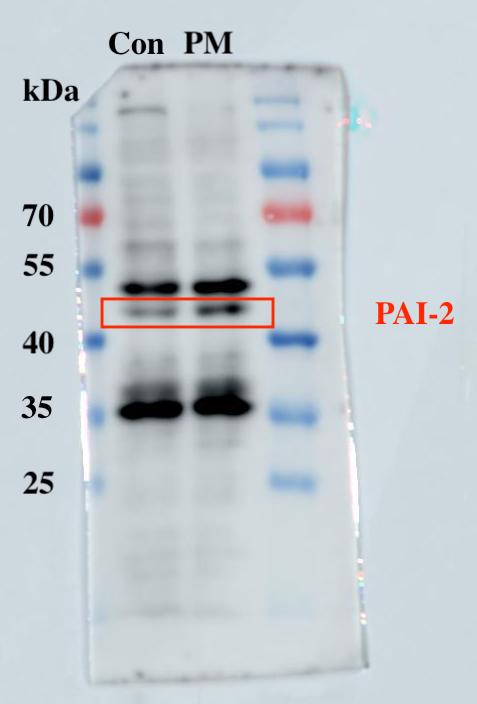

Supplement: Supplementary file 9 — Source data Fig. 3 [file 44321_2025_341_MOESM9_ESM.zip › Figure 3/3C/WB PAI-2.jpg]

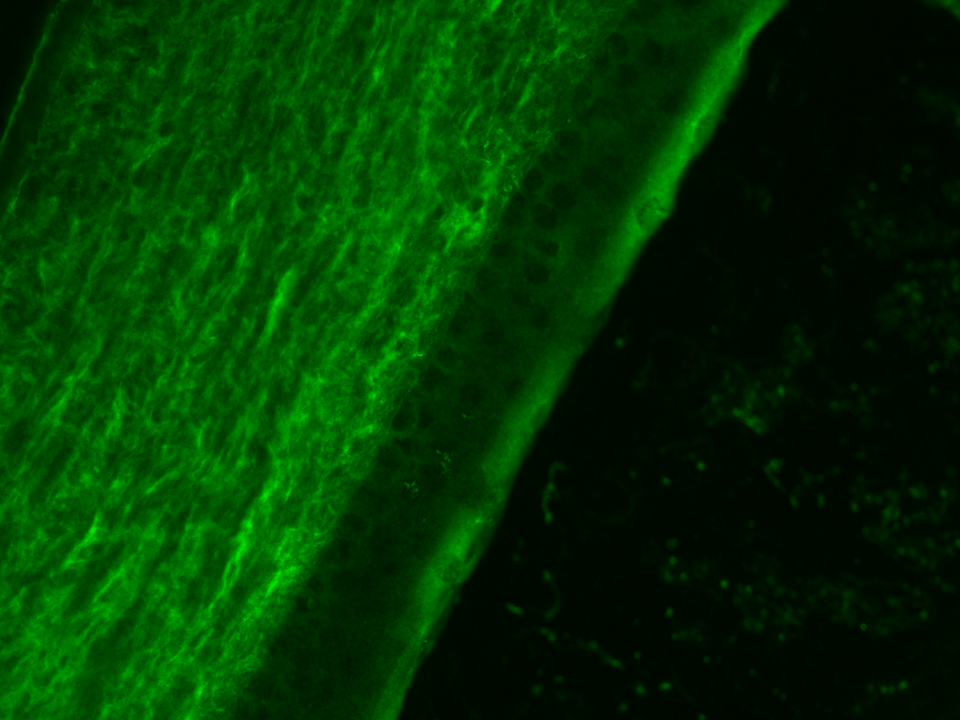

Supplement: Supplementary file 9 — Source data Fig. 3 [file 44321_2025_341_MOESM9_ESM.zip › Figure 3/3A/Immunofluorescence_PM PAI-2.tif]

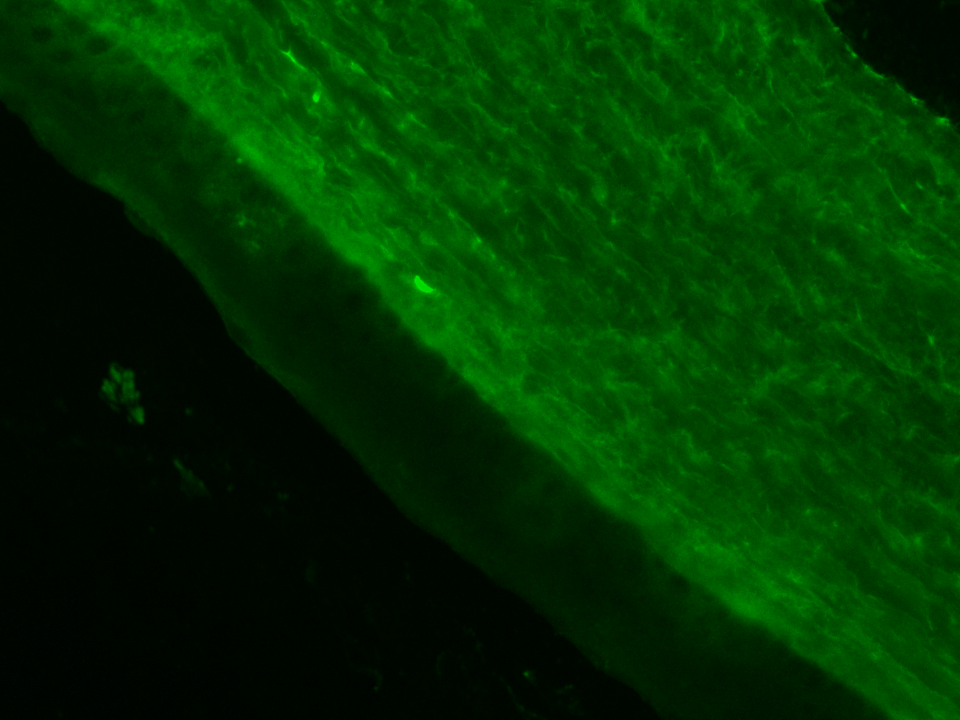

Supplement: Supplementary file 9 — Source data Fig. 3 [file 44321_2025_341_MOESM9_ESM.zip › Figure 3/3A/Immunofluorescence_PBS PAI-2.tif]

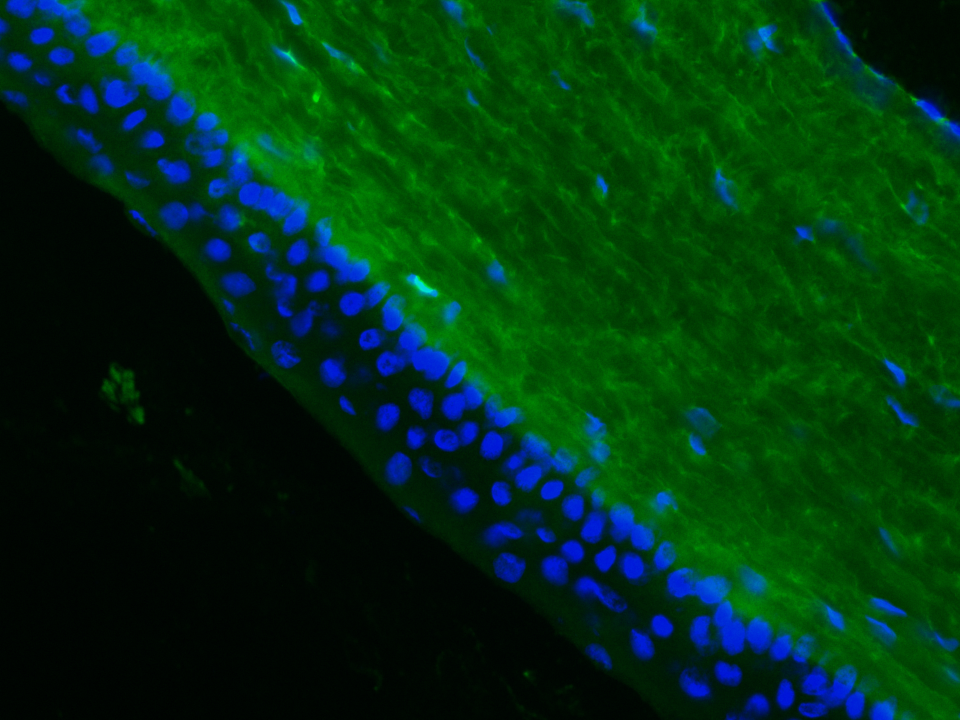

Supplement: Supplementary file 9 — Source data Fig. 3 [file 44321_2025_341_MOESM9_ESM.zip › Figure 3/3A/Immunofluorescence_PBS merge.tif]

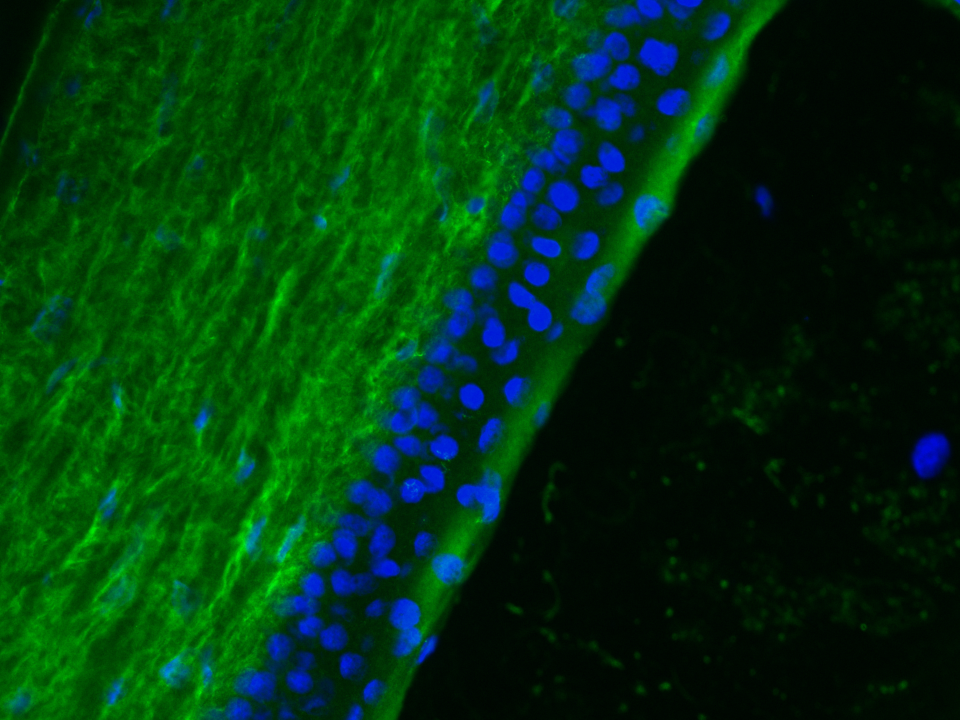

Supplement: Supplementary file 9 — Source data Fig. 3 [file 44321_2025_341_MOESM9_ESM.zip › Figure 3/3A/Immunofluorescence_PM merge.tif]

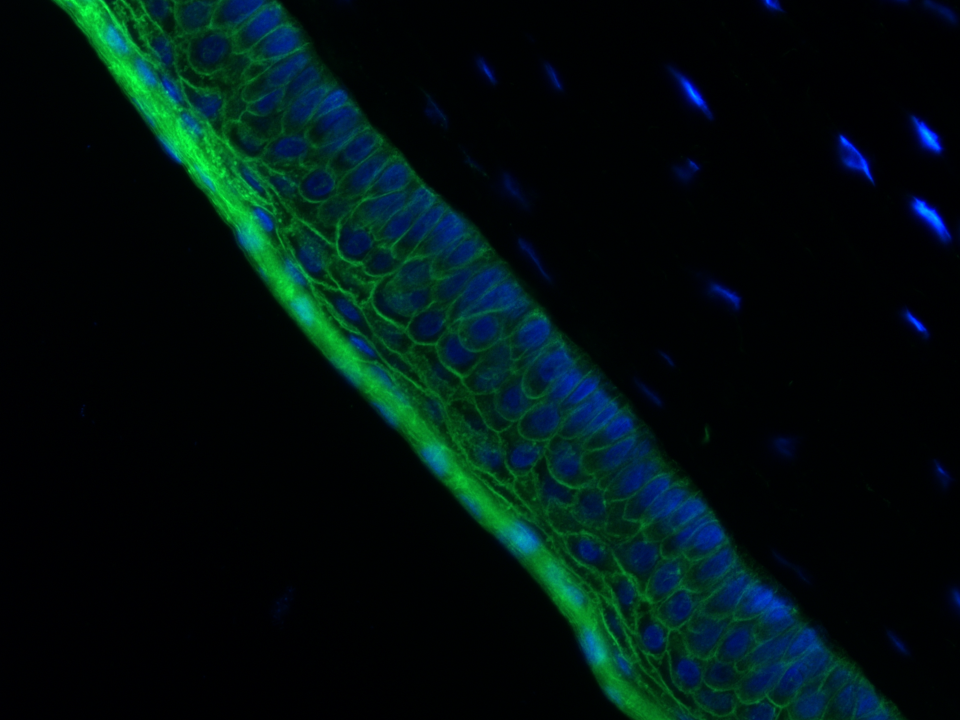

Supplement: Supplementary file 10 — Source data Fig. 4 [file 44321_2025_341_MOESM10_ESM.zip › Figure 4/4E/Immunofluorescence_PBS merge.tif]

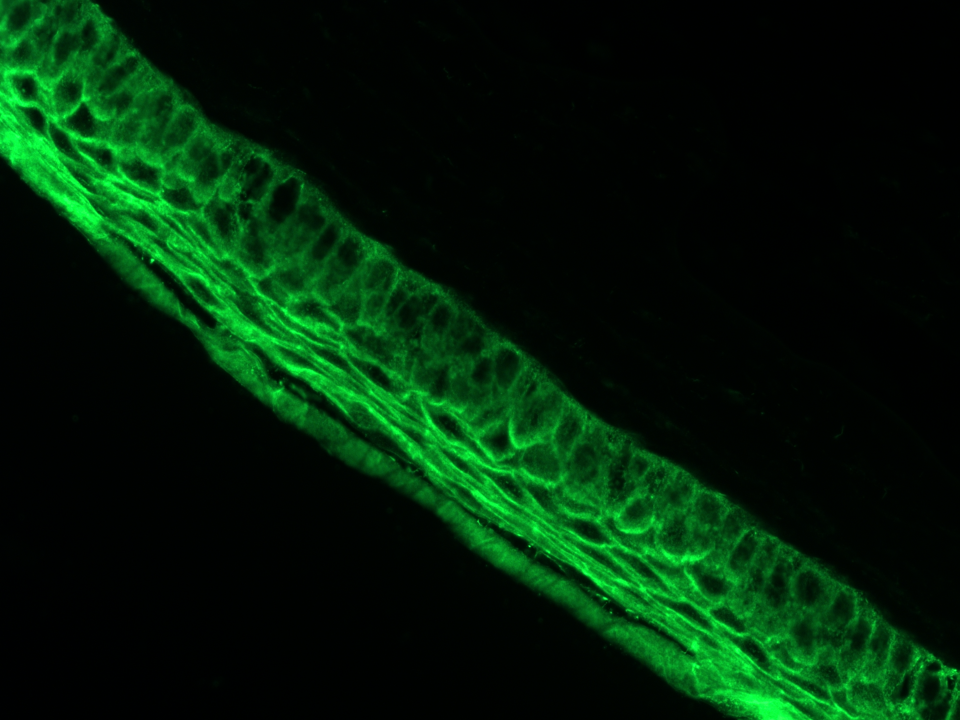

Supplement: Supplementary file 10 — Source data Fig. 4 [file 44321_2025_341_MOESM10_ESM.zip › Figure 4/4E/Immunofluorescence_PM F-actin.tif]

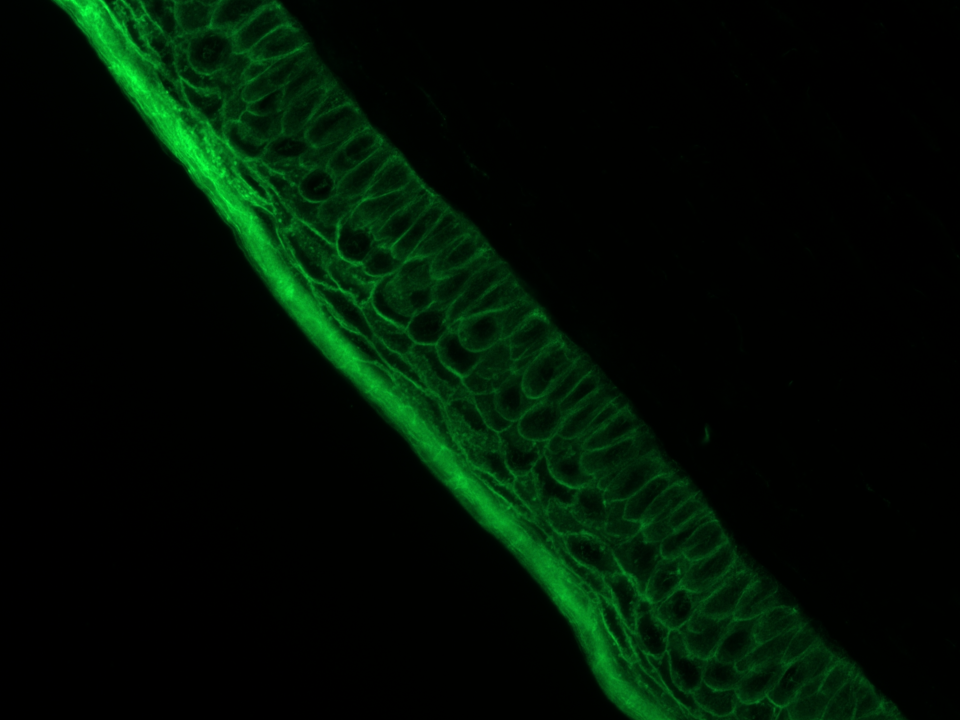

Supplement: Supplementary file 10 — Source data Fig. 4 [file 44321_2025_341_MOESM10_ESM.zip › Figure 4/4E/Immunofluorescence_PBS F-actin.tif]

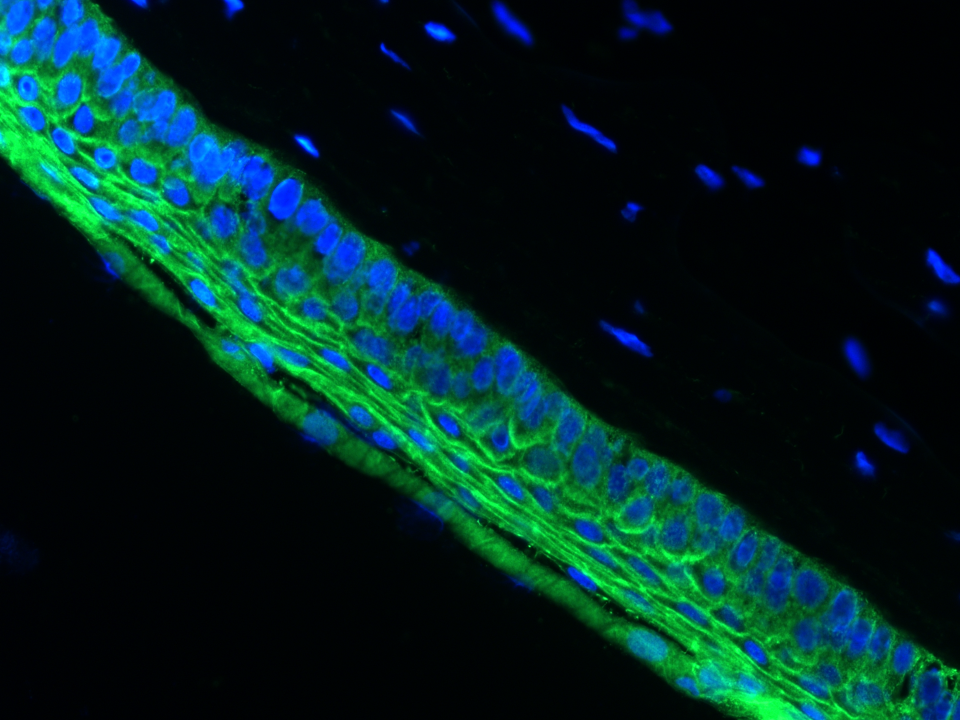

Supplement: Supplementary file 10 — Source data Fig. 4 [file 44321_2025_341_MOESM10_ESM.zip › Figure 4/4E/Immunofluorescence_PM merge.tif]

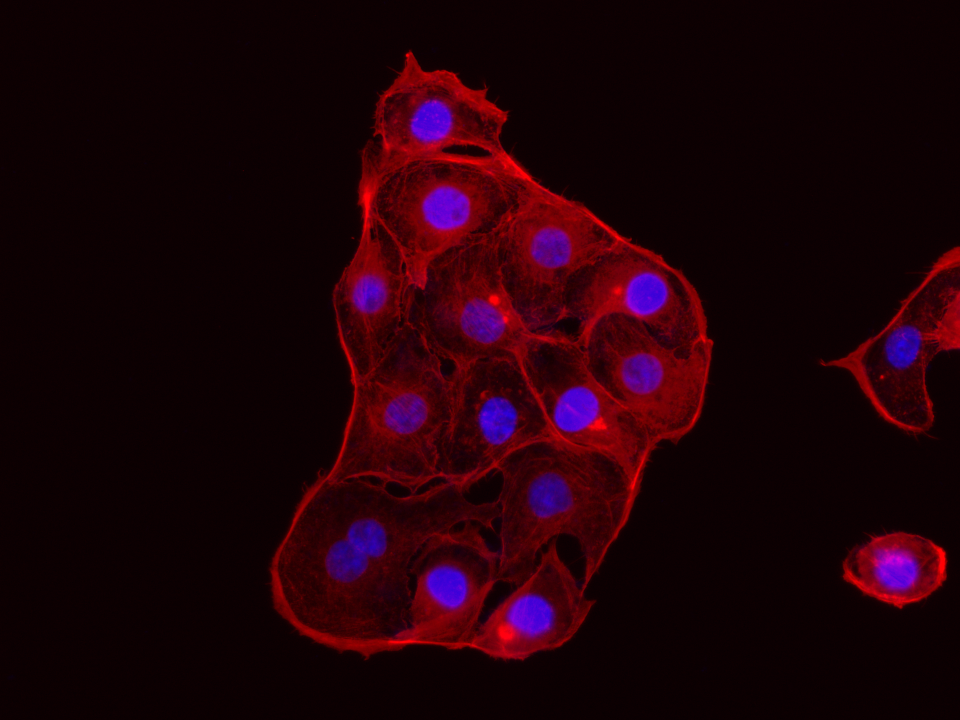

Supplement: Supplementary file 10 — Source data Fig. 4 [file 44321_2025_341_MOESM10_ESM.zip › Figure 4/4F-4H/Immunofluorescence_NC merge.tif]

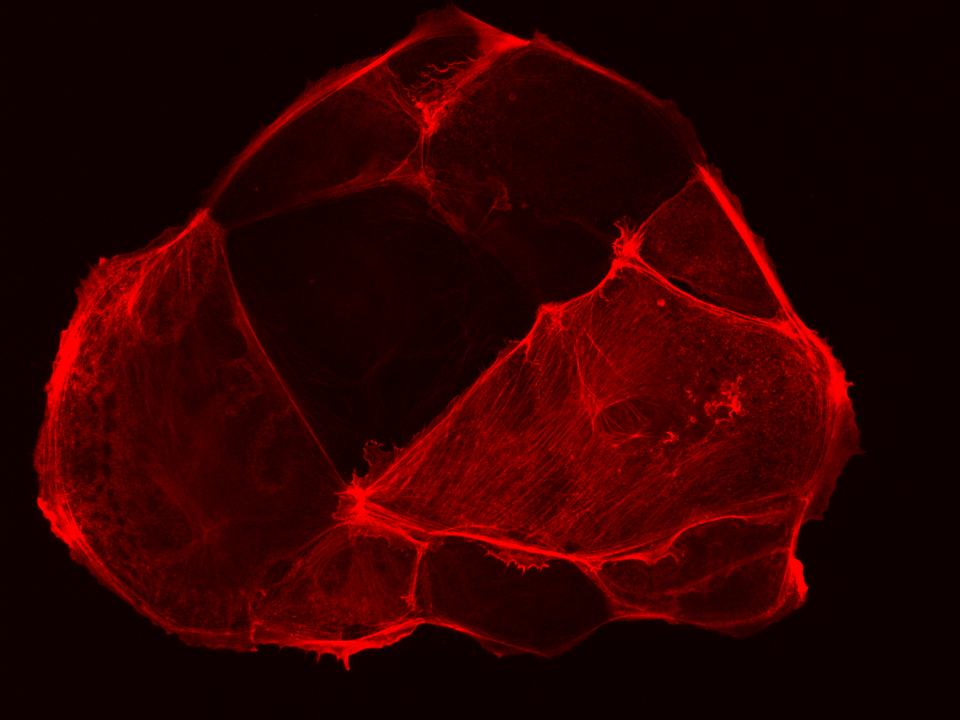

Supplement: Supplementary file 10 — Source data Fig. 4 [file 44321_2025_341_MOESM10_ESM.zip › Figure 4/4F-4H/Immunofluorescence_KO+PM+Jasp F-actin.tif]

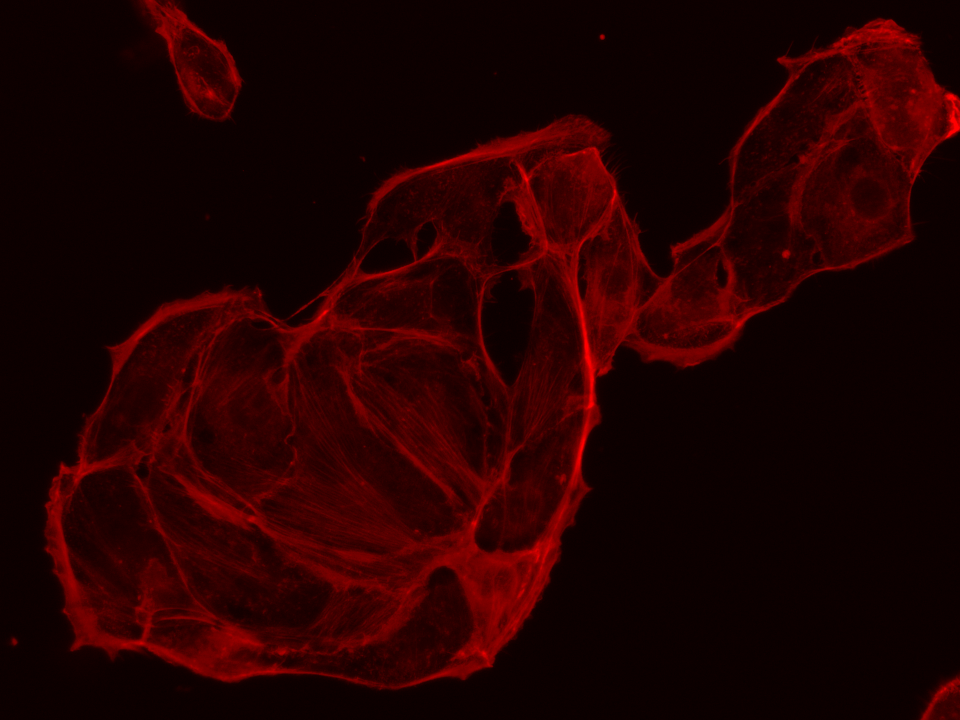

Supplement: Supplementary file 10 — Source data Fig. 4 [file 44321_2025_341_MOESM10_ESM.zip › Figure 4/4F-4H/Immunofluorescence_NC+PM F-actin.tif]

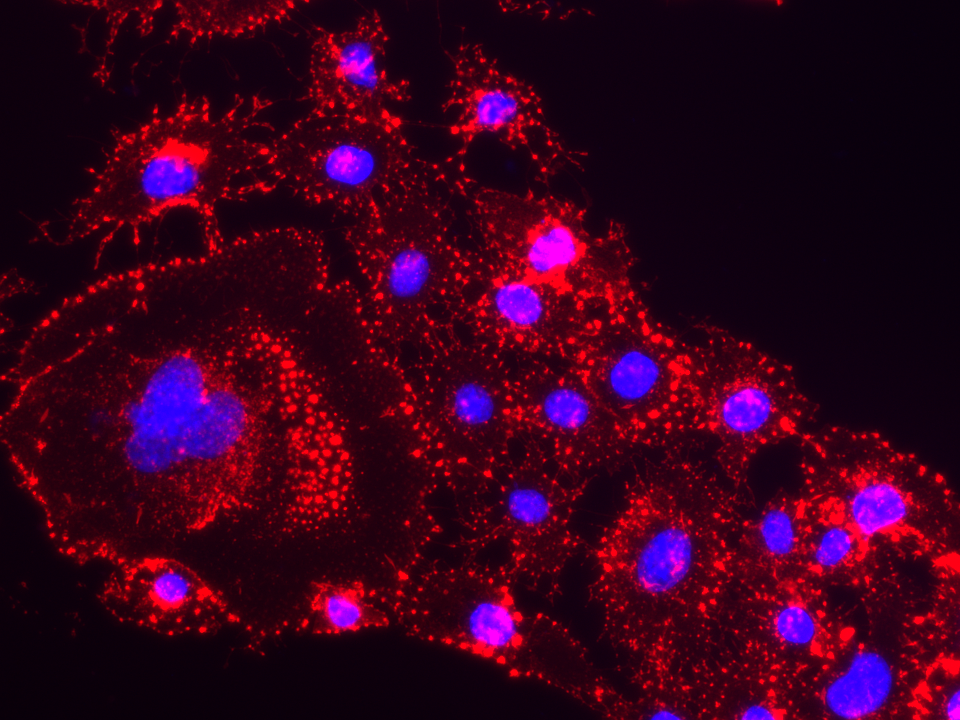

Supplement: Supplementary file 10 — Source data Fig. 4 [file 44321_2025_341_MOESM10_ESM.zip › Figure 4/4F-4H/Immunofluorescence_KO+PM+CytoD merge.tif]

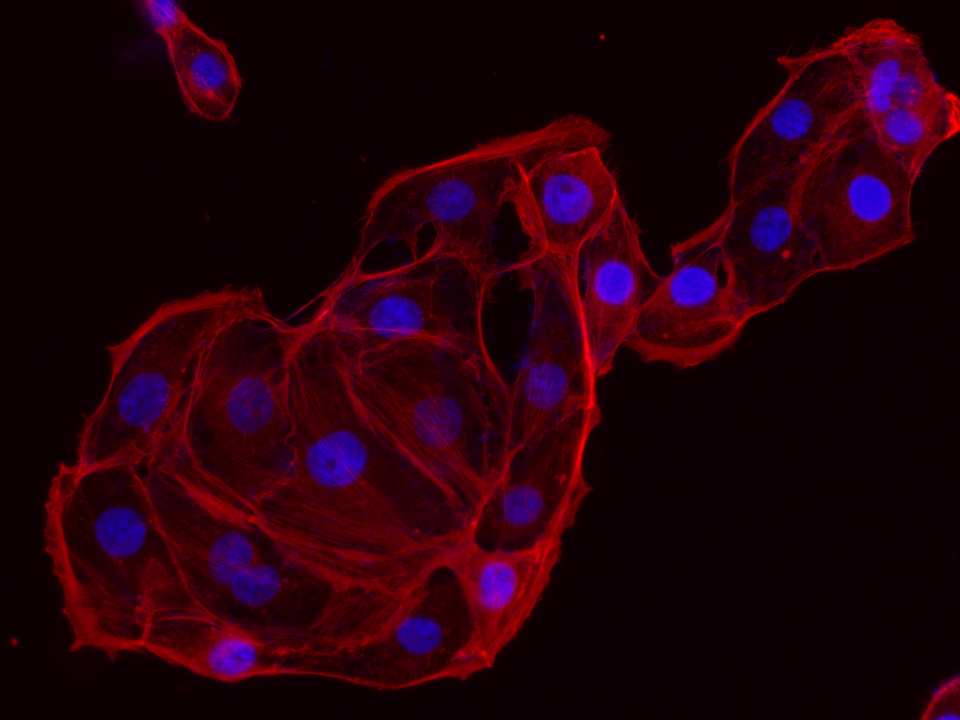

Supplement: Supplementary file 10 — Source data Fig. 4 [file 44321_2025_341_MOESM10_ESM.zip › Figure 4/4F-4H/Immunofluorescence_NC+PM merge.tif]

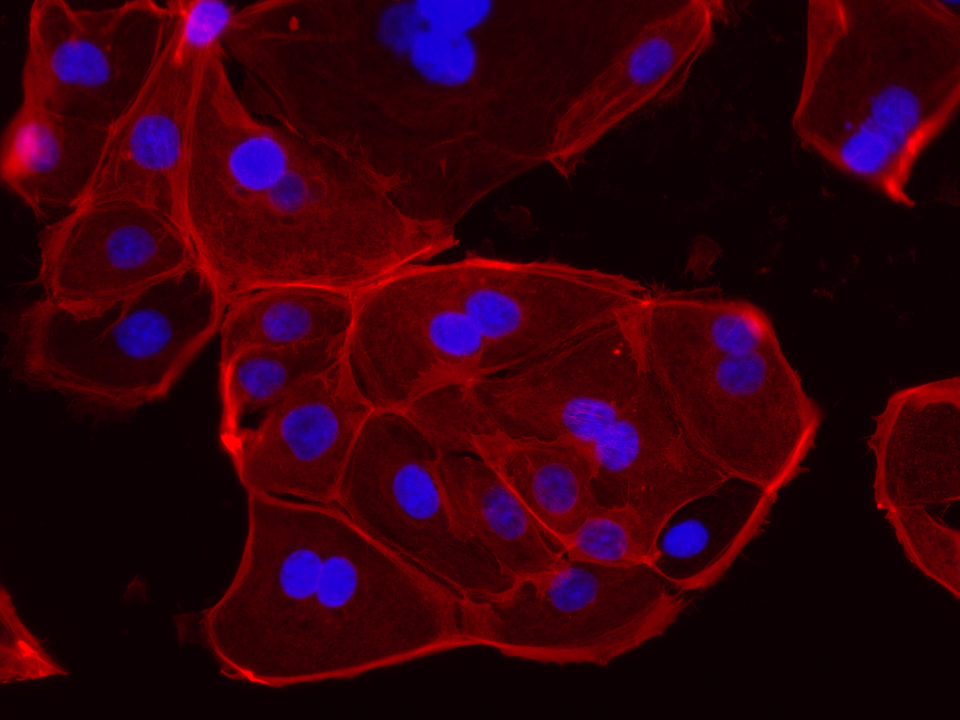

Supplement: Supplementary file 10 — Source data Fig. 4 [file 44321_2025_341_MOESM10_ESM.zip › Figure 4/4F-4H/Immunofluorescence_KO merge.tif]

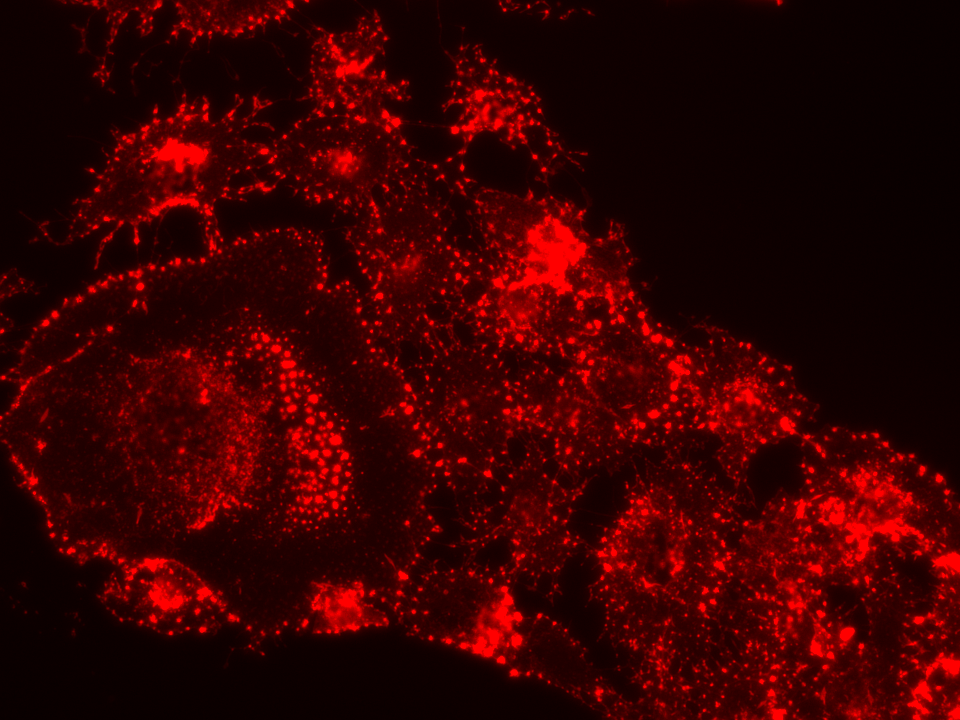

Supplement: Supplementary file 10 — Source data Fig. 4 [file 44321_2025_341_MOESM10_ESM.zip › Figure 4/4F-4H/Immunofluorescence_KO+PM+CytoD F-actin.tif]

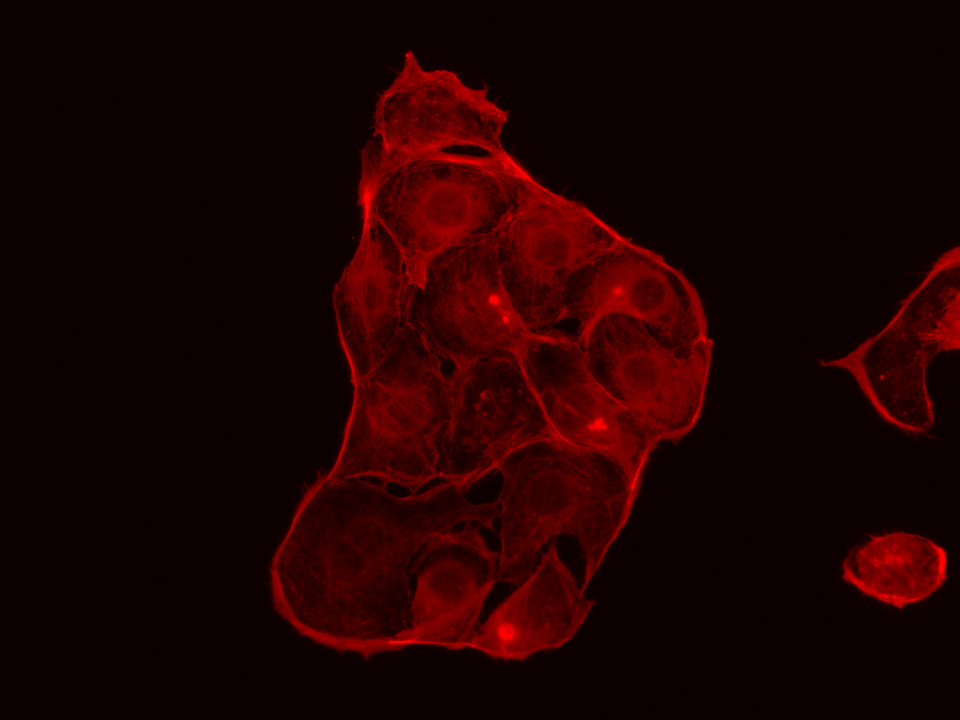

Supplement: Supplementary file 10 — Source data Fig. 4 [file 44321_2025_341_MOESM10_ESM.zip › Figure 4/4F-4H/Immunofluorescence_NC F-actin.tif]

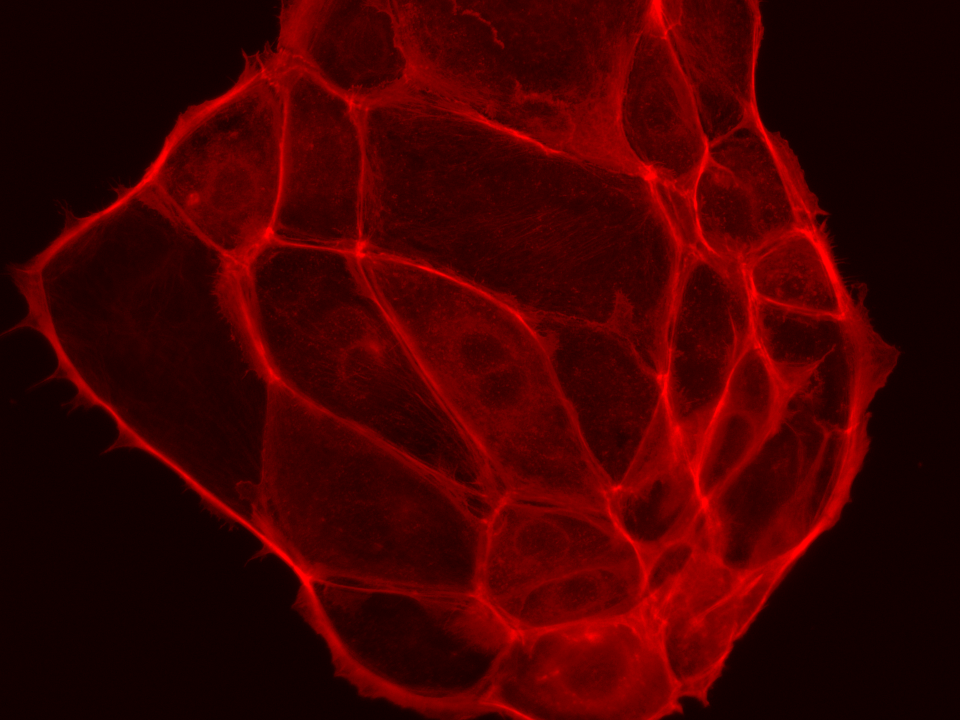

Supplement: Supplementary file 10 — Source data Fig. 4 [file 44321_2025_341_MOESM10_ESM.zip › Figure 4/4F-4H/Immunofluorescence_KO+PM F-actin.tif]

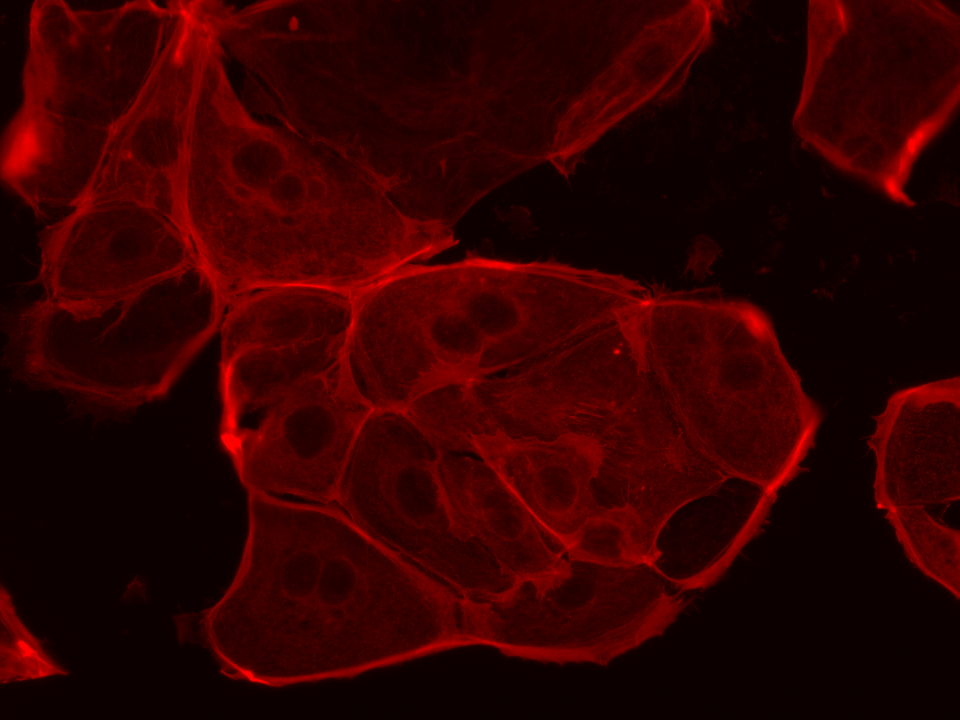

Supplement: Supplementary file 10 — Source data Fig. 4 [file 44321_2025_341_MOESM10_ESM.zip › Figure 4/4F-4H/Immunofluorescence_KO F-actin.tif]

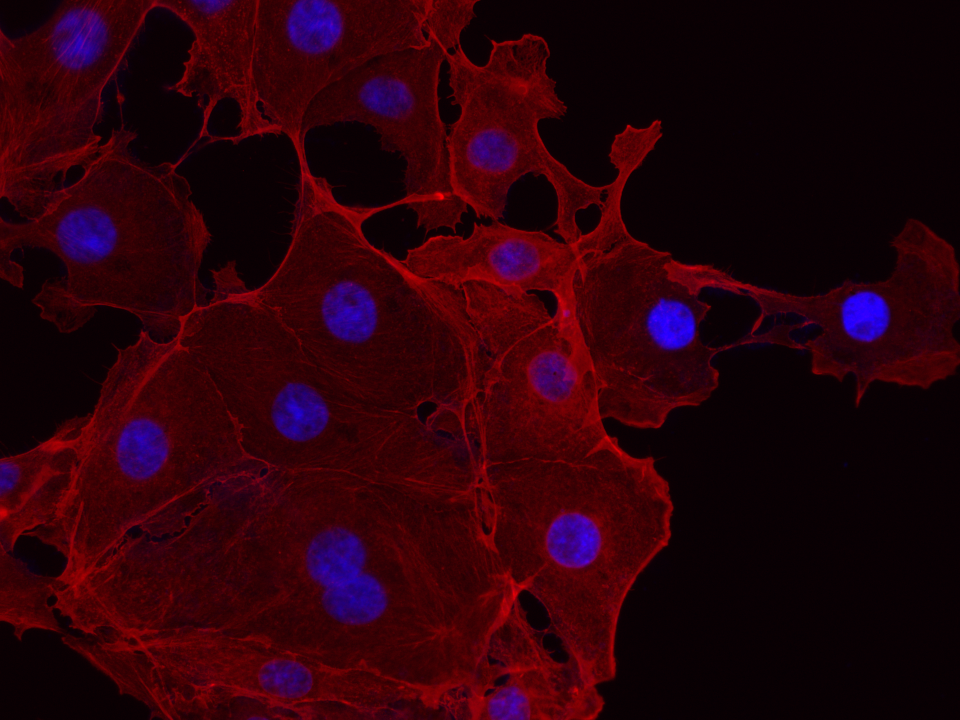

Supplement: Supplementary file 10 — Source data Fig. 4 [file 44321_2025_341_MOESM10_ESM.zip › Figure 4/4F-4H/Immunofluorescence_NC+PM+Blebb merge.tif]

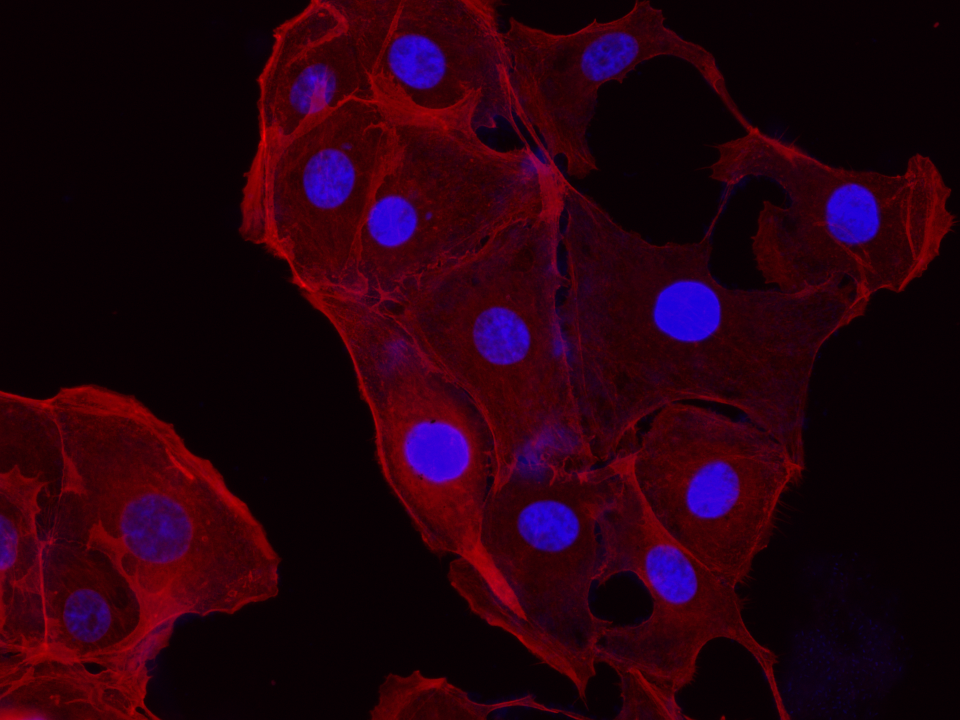

Supplement: Supplementary file 10 — Source data Fig. 4 [file 44321_2025_341_MOESM10_ESM.zip › Figure 4/4F-4H/Immunofluorescence_NC+PM+RI merge.tif]

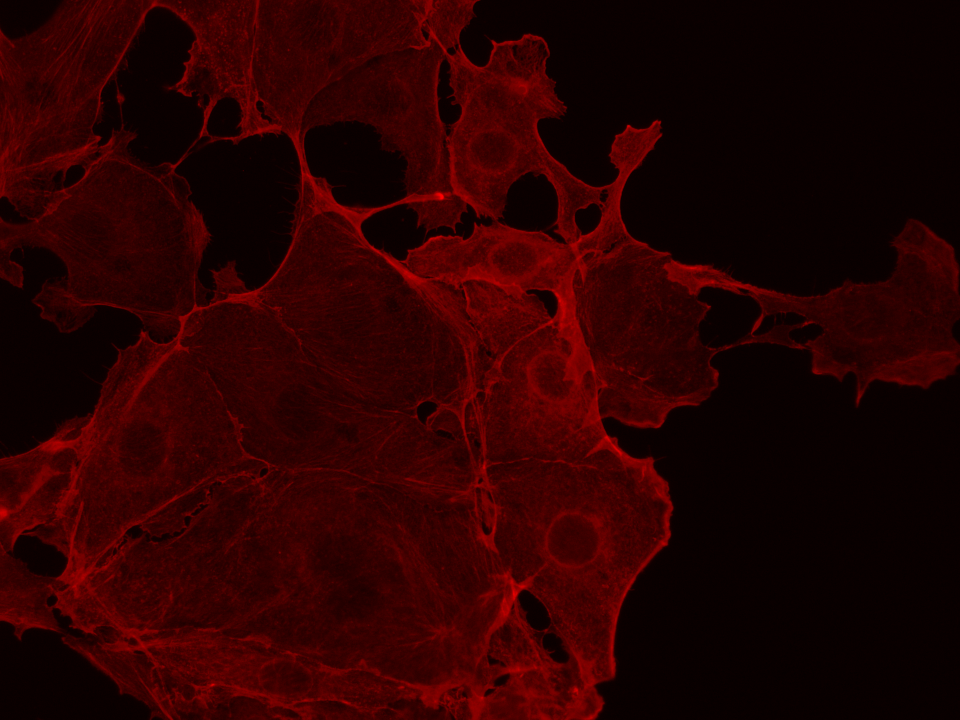

Supplement: Supplementary file 10 — Source data Fig. 4 [file 44321_2025_341_MOESM10_ESM.zip › Figure 4/4F-4H/Immunofluorescence_NC+PM+Blebb F-actin.tif]

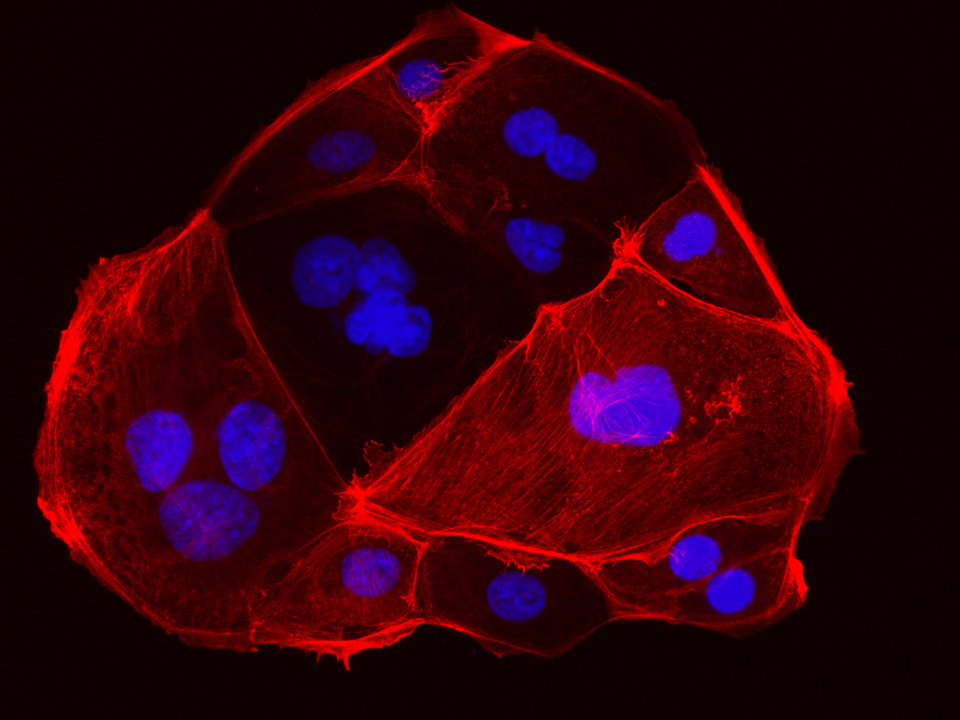

Supplement: Supplementary file 10 — Source data Fig. 4 [file 44321_2025_341_MOESM10_ESM.zip › Figure 4/4F-4H/Immunofluorescence_KO+PM+Jasp merge.tif]

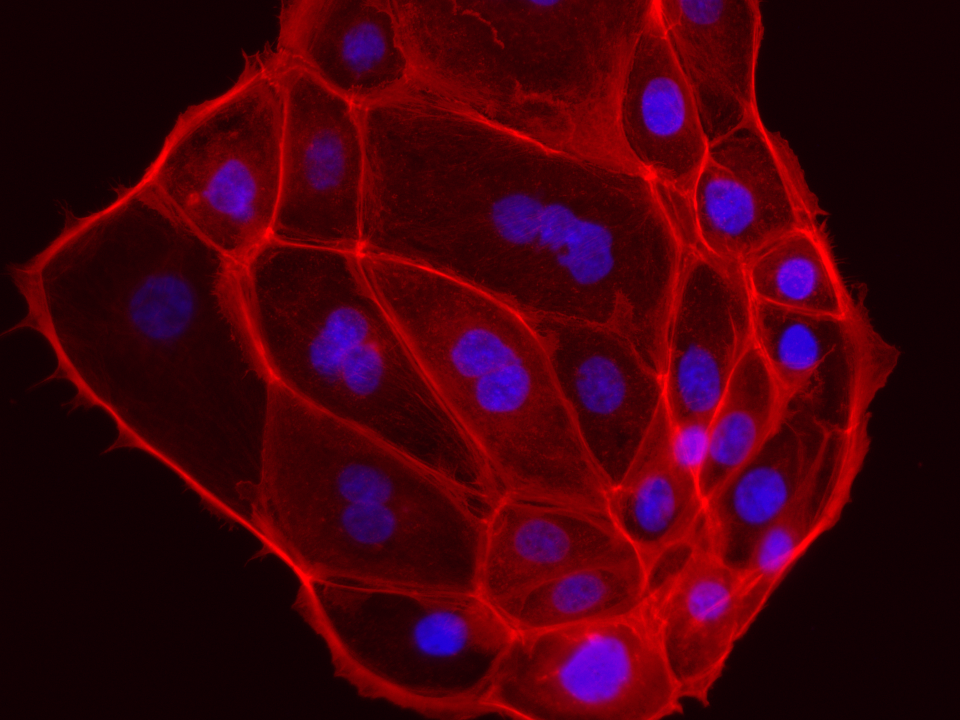

Supplement: Supplementary file 10 — Source data Fig. 4 [file 44321_2025_341_MOESM10_ESM.zip › Figure 4/4F-4H/Immunofluorescence_KO+PM merge.tif]

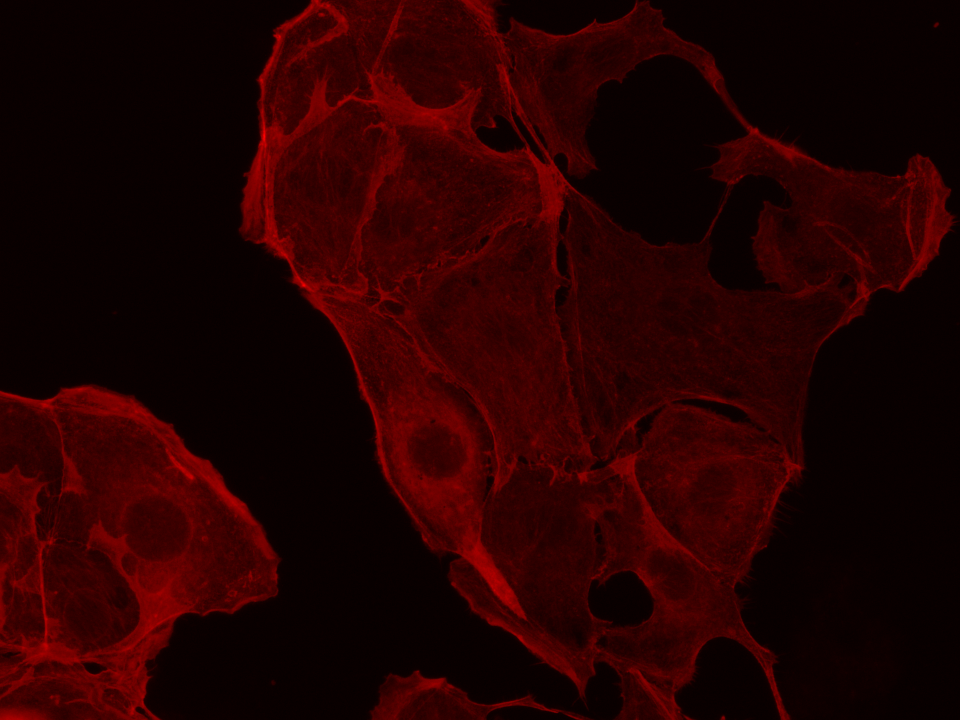

Supplement: Supplementary file 10 — Source data Fig. 4 [file 44321_2025_341_MOESM10_ESM.zip › Figure 4/4F-4H/Immunofluorescence_NC+PM+RI F-actin.tif]

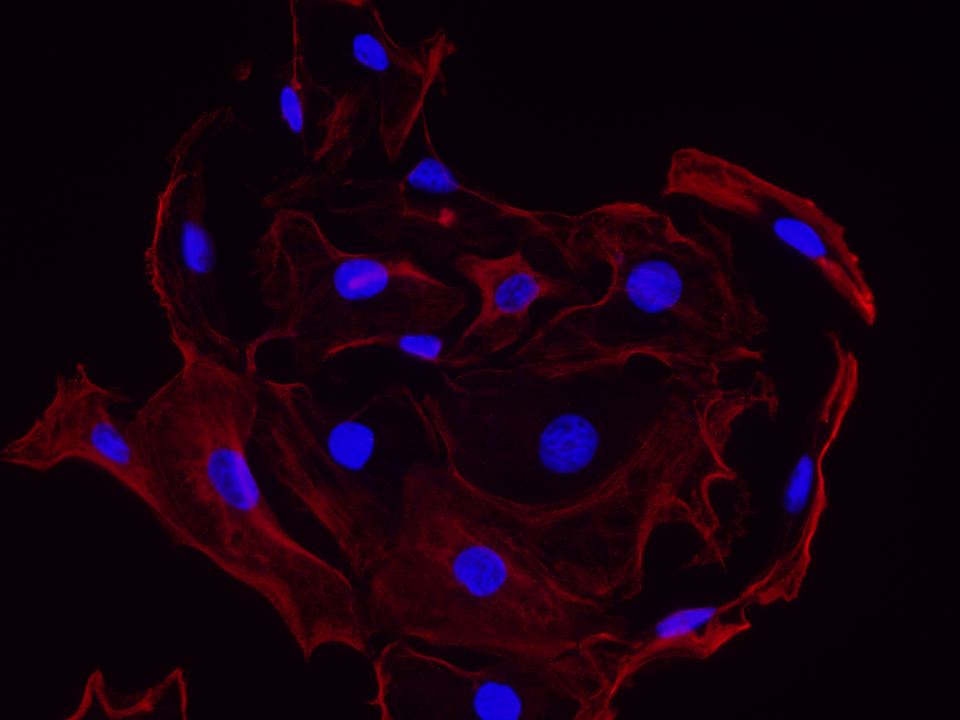

Supplement: Supplementary file 10 — Source data Fig. 4 [file 44321_2025_341_MOESM10_ESM.zip › Figure 4/4A/Immunofluorescence_NC merge.tif]

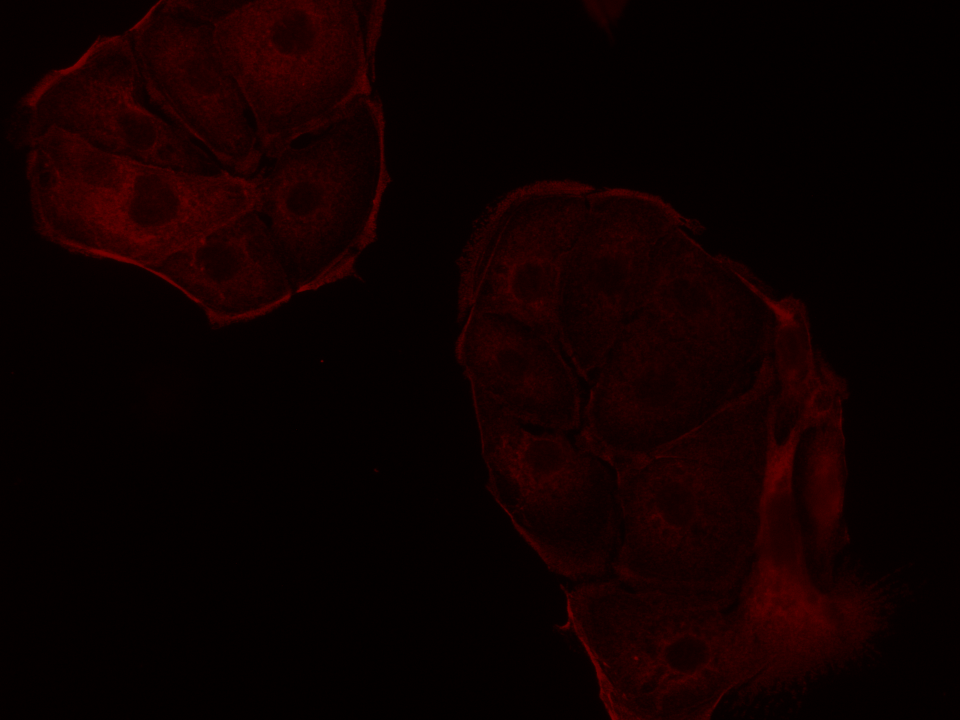

Supplement: Supplementary file 10 — Source data Fig. 4 [file 44321_2025_341_MOESM10_ESM.zip › Figure 4/4A/Immunofluorescence_KO p-myosin IIa.tif]

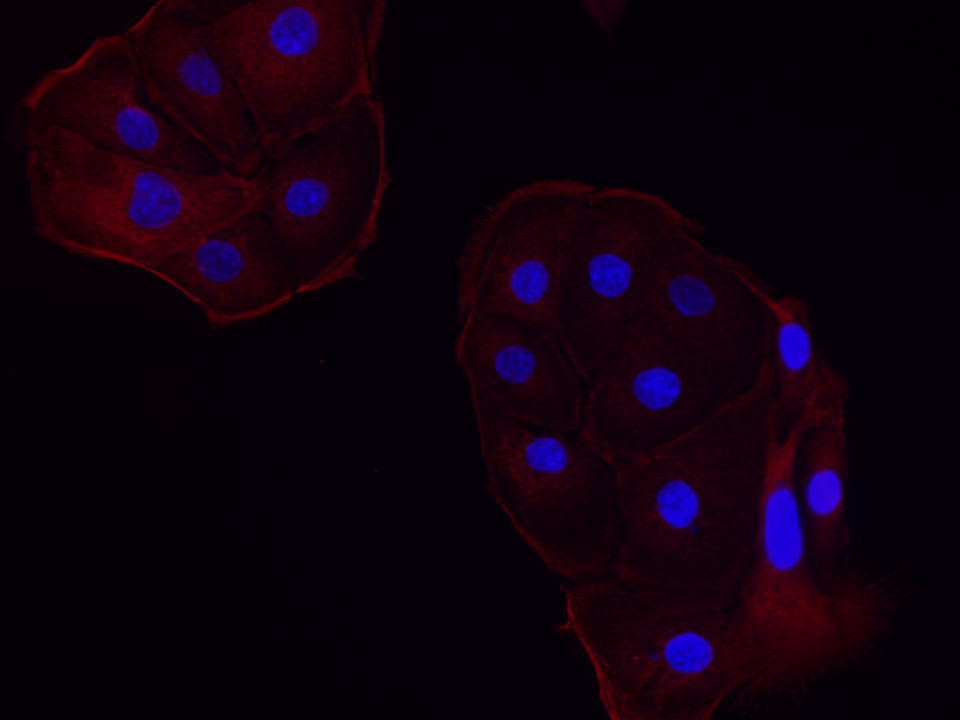

Supplement: Supplementary file 10 — Source data Fig. 4 [file 44321_2025_341_MOESM10_ESM.zip › Figure 4/4A/Immunofluorescence_KO merge.tif]

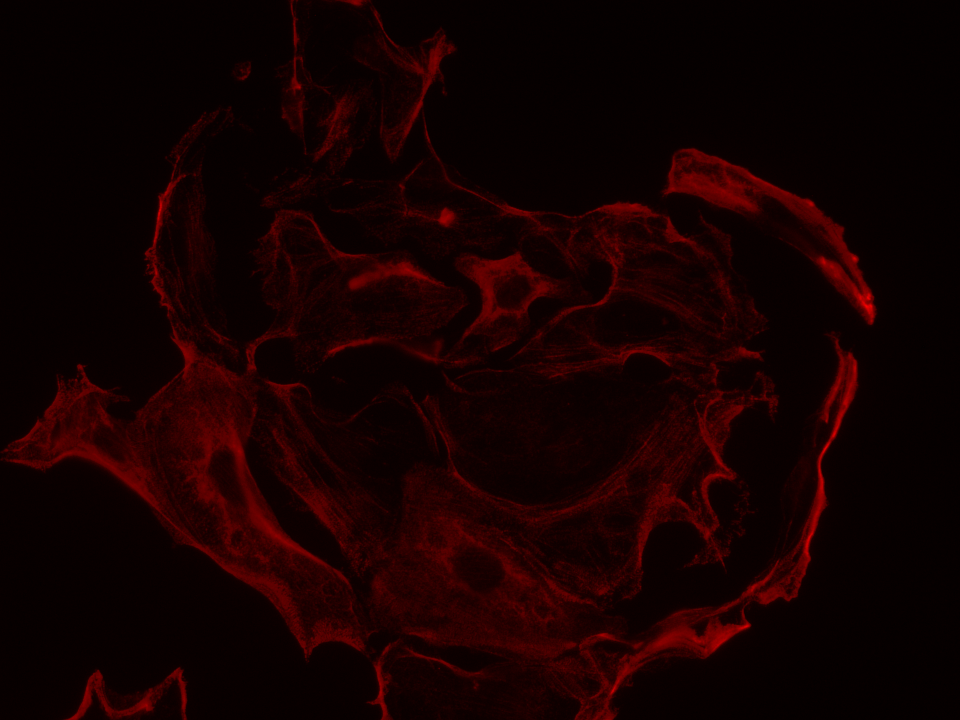

Supplement: Supplementary file 10 — Source data Fig. 4 [file 44321_2025_341_MOESM10_ESM.zip › Figure 4/4A/Immunofluorescence_NC p-myosin IIa.tif]

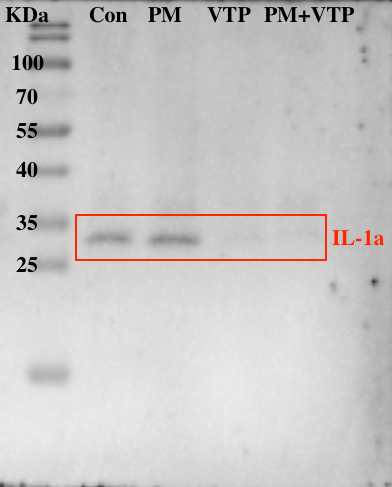

Supplement: Supplementary file 11 — Source data Fig. 5 [file 44321_2025_341_MOESM11_ESM.zip › Figure 5/5G/WB IL-1a.tif]

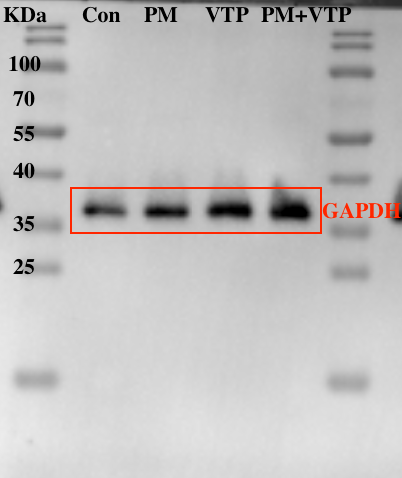

Supplement: Supplementary file 11 — Source data Fig. 5 [file 44321_2025_341_MOESM11_ESM.zip › Figure 5/5G/WB GAPDH.tif]

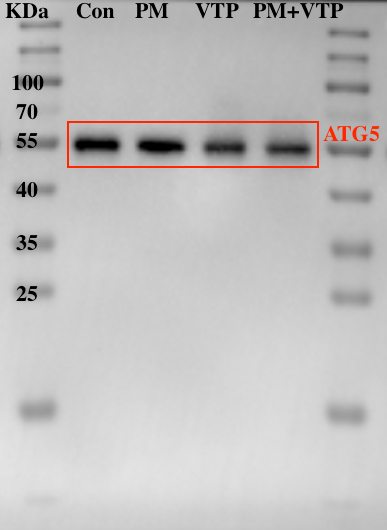

Supplement: Supplementary file 11 — Source data Fig. 5 [file 44321_2025_341_MOESM11_ESM.zip › Figure 5/5G/WB ATG5.tif]

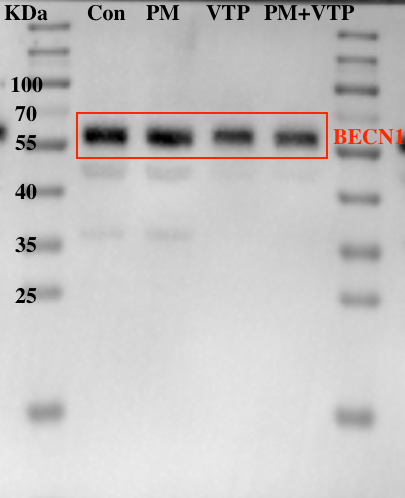

Supplement: Supplementary file 11 — Source data Fig. 5 [file 44321_2025_341_MOESM11_ESM.zip › Figure 5/5G/WB BECN1.tif]

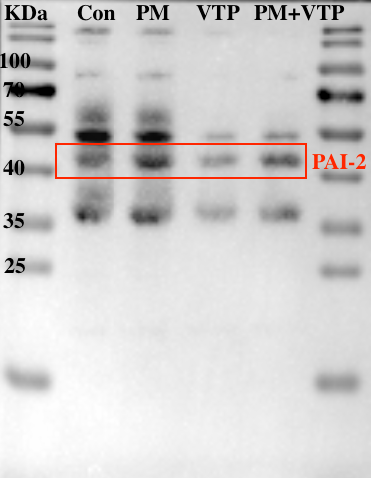

Supplement: Supplementary file 11 — Source data Fig. 5 [file 44321_2025_341_MOESM11_ESM.zip › Figure 5/5G/WB PAI-2.tif]

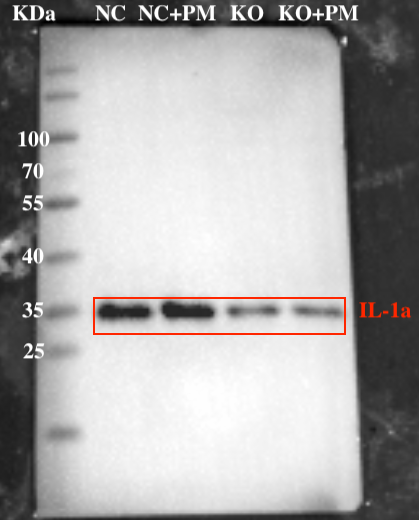

Supplement: Supplementary file 11 — Source data Fig. 5 [file 44321_2025_341_MOESM11_ESM.zip › Figure 5/5F/WB IL-1a.tif]

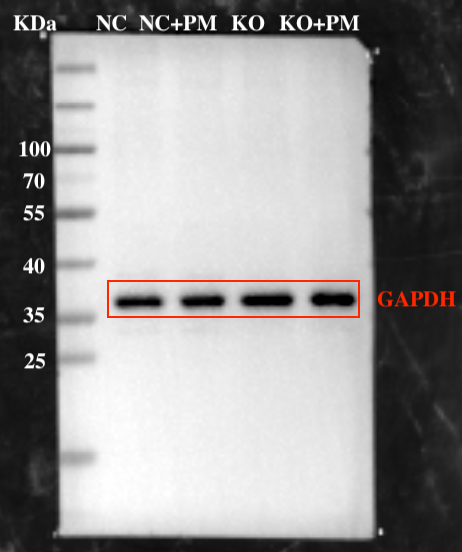

Supplement: Supplementary file 11 — Source data Fig. 5 [file 44321_2025_341_MOESM11_ESM.zip › Figure 5/5F/WB GAPDH.tif]

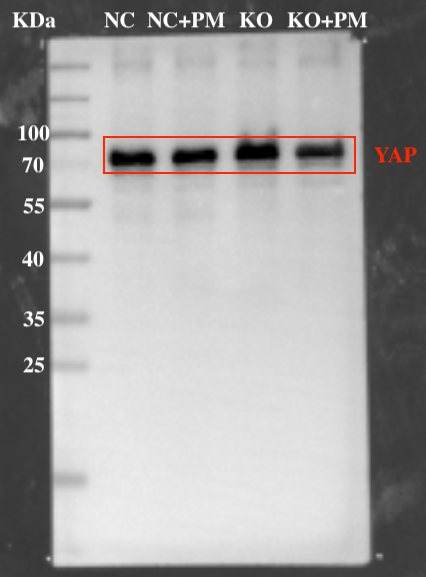

Supplement: Supplementary file 11 — Source data Fig. 5 [file 44321_2025_341_MOESM11_ESM.zip › Figure 5/5F/WB YAP.tif]

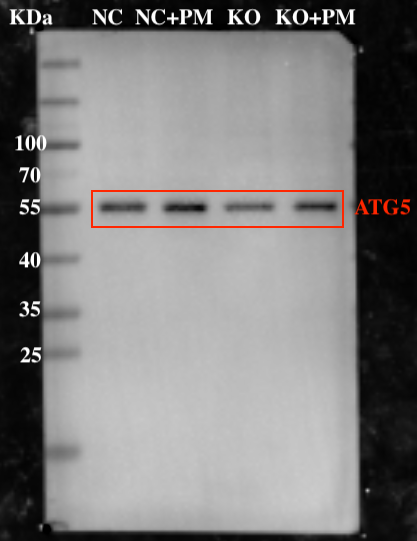

Supplement: Supplementary file 11 — Source data Fig. 5 [file 44321_2025_341_MOESM11_ESM.zip › Figure 5/5F/WB ATG5.tif]

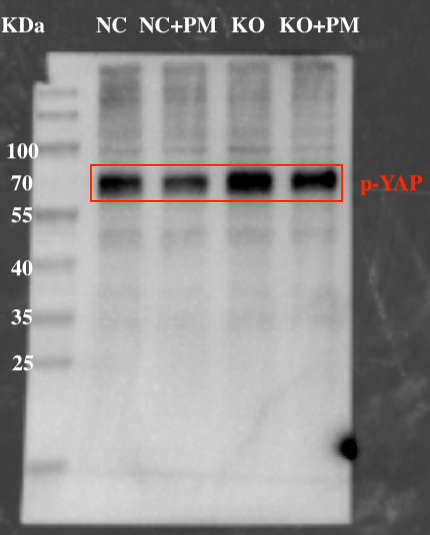

Supplement: Supplementary file 11 — Source data Fig. 5 [file 44321_2025_341_MOESM11_ESM.zip › Figure 5/5F/WB p-YAP.tif]

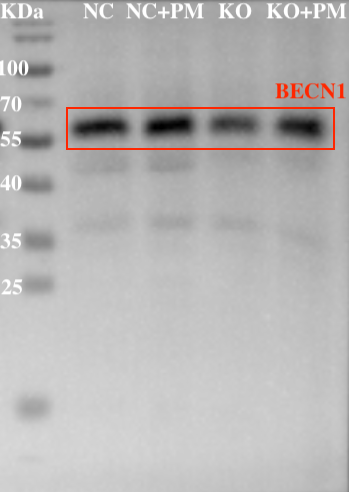

Supplement: Supplementary file 11 — Source data Fig. 5 [file 44321_2025_341_MOESM11_ESM.zip › Figure 5/5F/WB BECN1.tif]

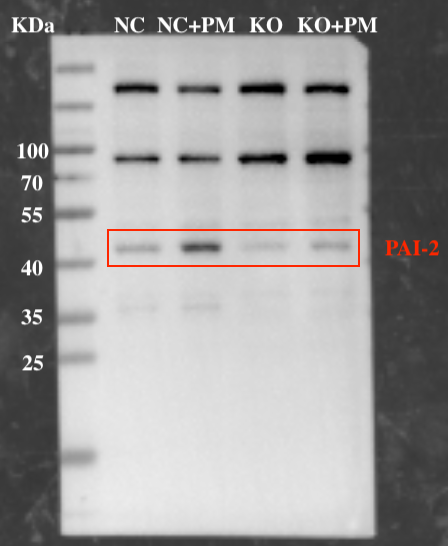

Supplement: Supplementary file 11 — Source data Fig. 5 [file 44321_2025_341_MOESM11_ESM.zip › Figure 5/5F/WB PAI-2.tif]

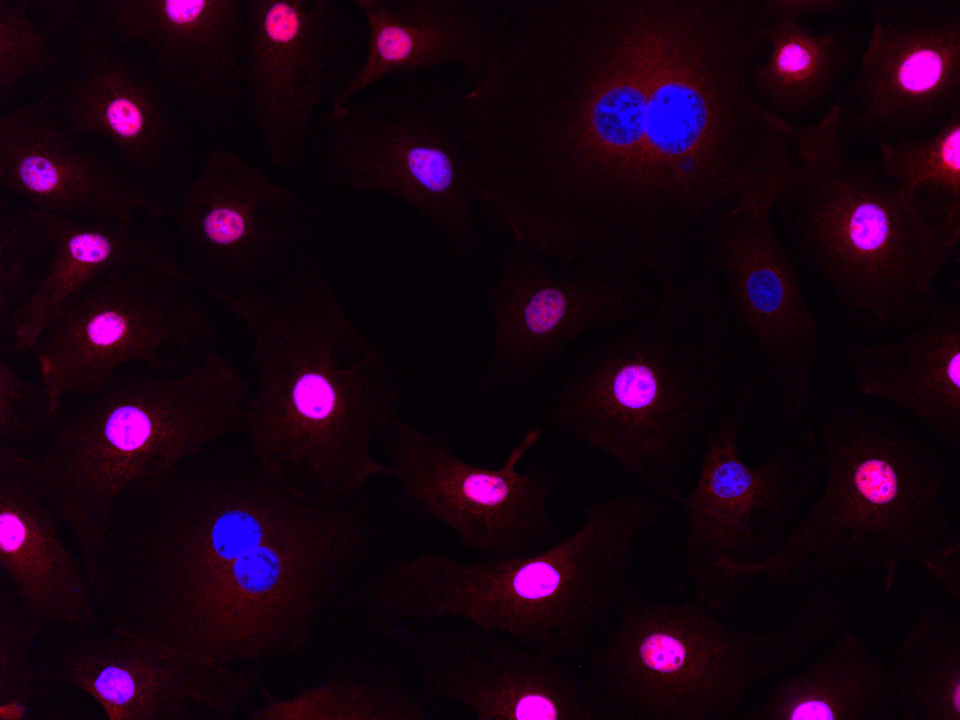

Supplement: Supplementary file 11 — Source data Fig. 5 [file 44321_2025_341_MOESM11_ESM.zip › Figure 5/5B&5D/Immunofluorescence_NC Control merge.tif]

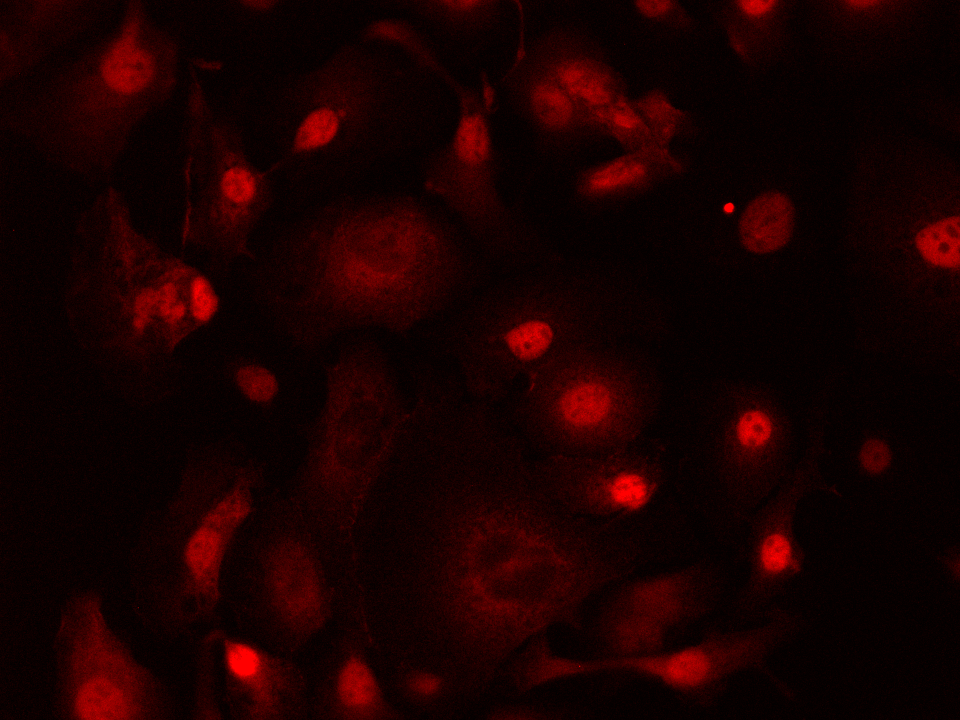

Supplement: Supplementary file 11 — Source data Fig. 5 [file 44321_2025_341_MOESM11_ESM.zip › Figure 5/5B&5D/Immunofluorescence_KO PM YAP.tif]

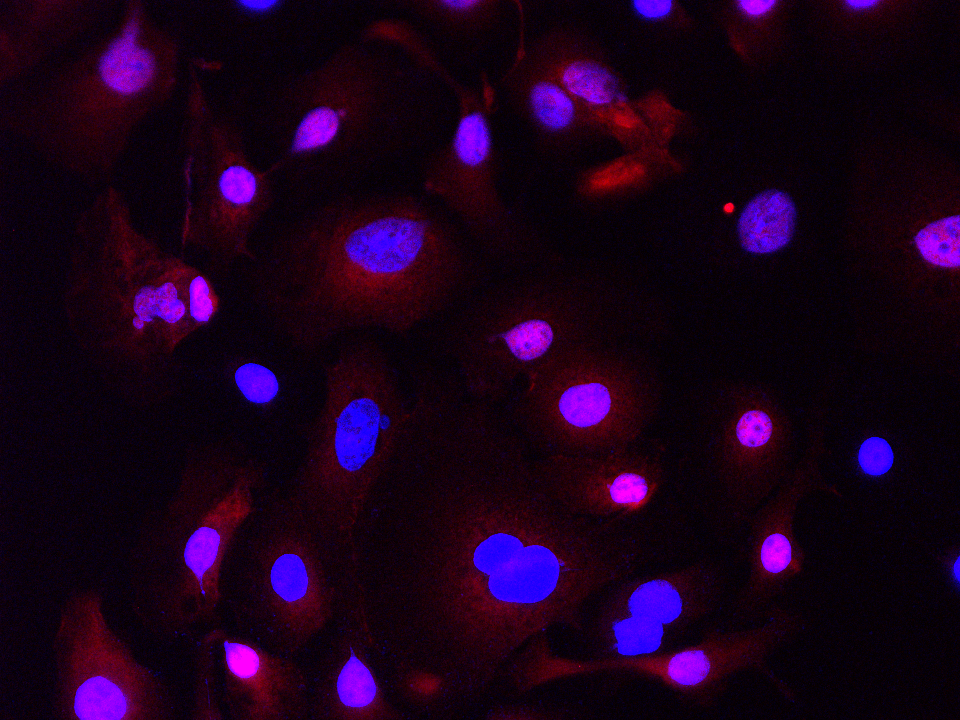

Supplement: Supplementary file 11 — Source data Fig. 5 [file 44321_2025_341_MOESM11_ESM.zip › Figure 5/5B&5D/Immunofluorescence_KO PM merge.tif]

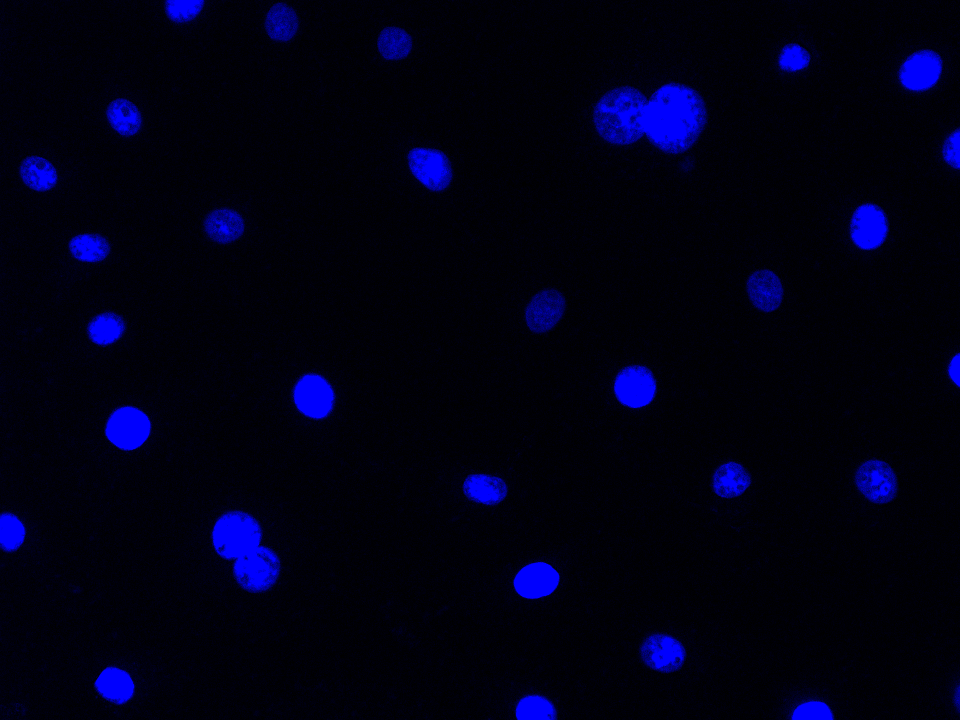

Supplement: Supplementary file 11 — Source data Fig. 5 [file 44321_2025_341_MOESM11_ESM.zip › Figure 5/5B&5D/Immunofluorescence_NC Control DAPI.tif]

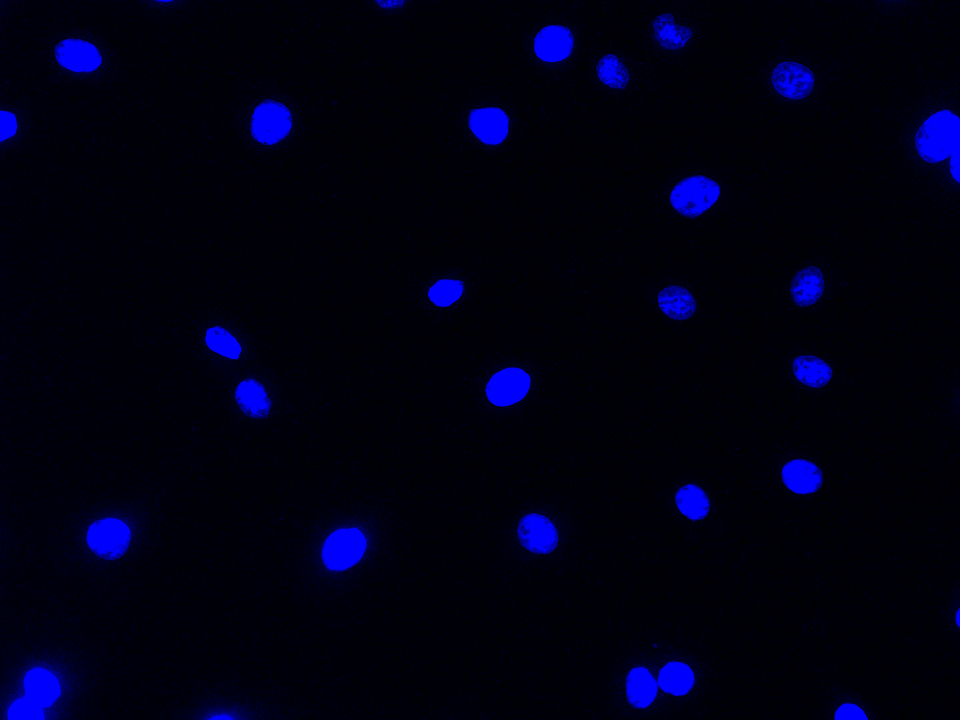

Supplement: Supplementary file 11 — Source data Fig. 5 [file 44321_2025_341_MOESM11_ESM.zip › Figure 5/5B&5D/Immunofluorescence_NC PM DAPI.tif]

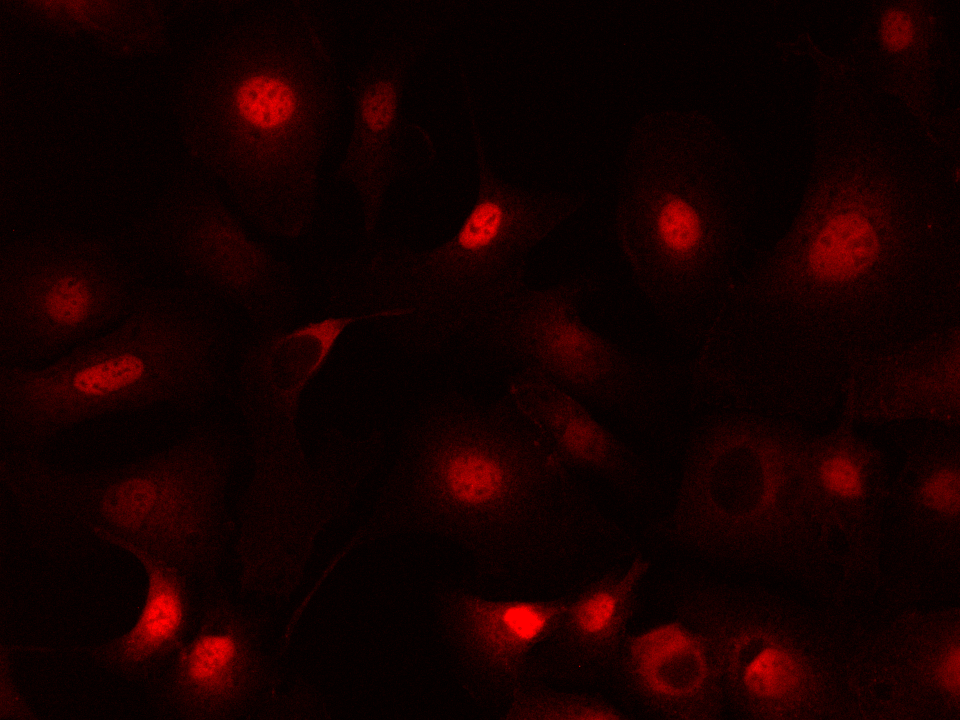

Supplement: Supplementary file 11 — Source data Fig. 5 [file 44321_2025_341_MOESM11_ESM.zip › Figure 5/5B&5D/Immunofluorescence_KO Control YAP.tif]

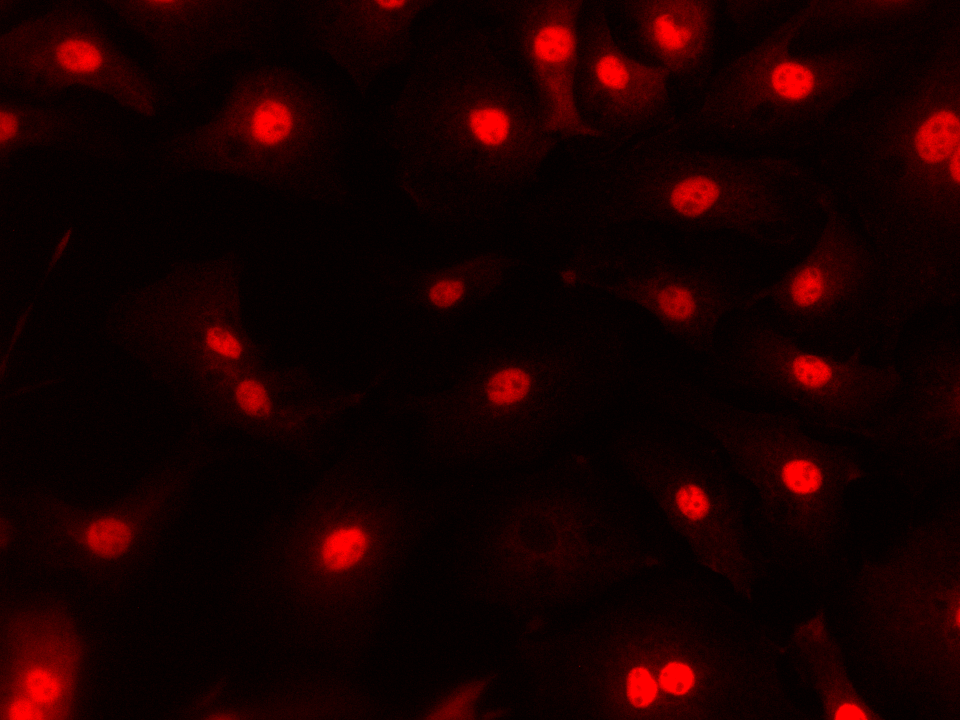

Supplement: Supplementary file 11 — Source data Fig. 5 [file 44321_2025_341_MOESM11_ESM.zip › Figure 5/5B&5D/Immunofluorescence_NC PM YAP.tif]

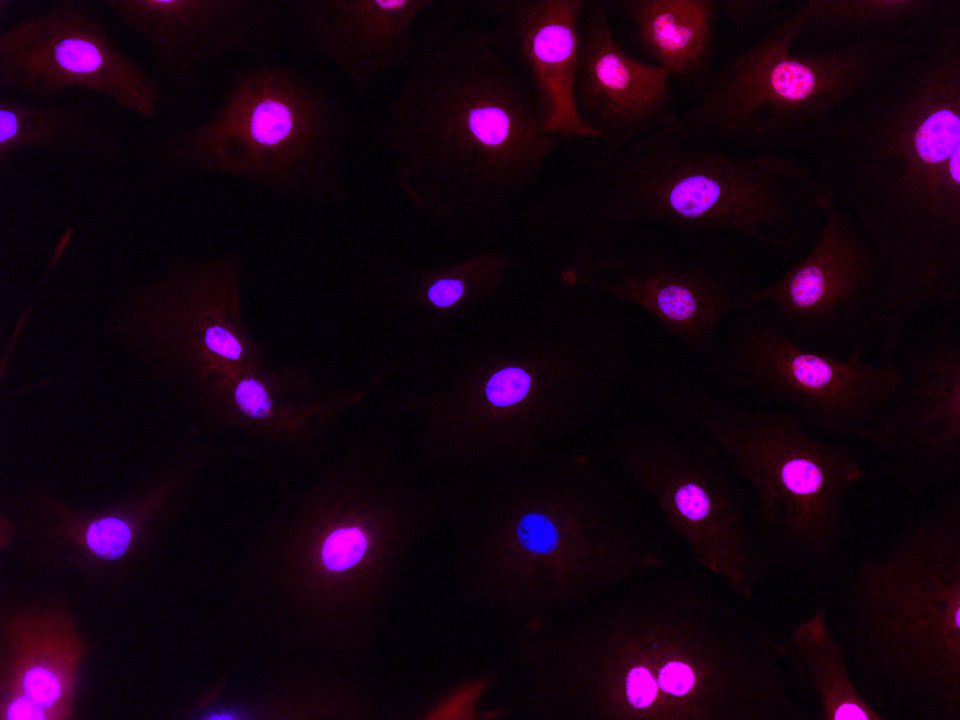

Supplement: Supplementary file 11 — Source data Fig. 5 [file 44321_2025_341_MOESM11_ESM.zip › Figure 5/5B&5D/Immunofluorescence_NC PM merge.tif]

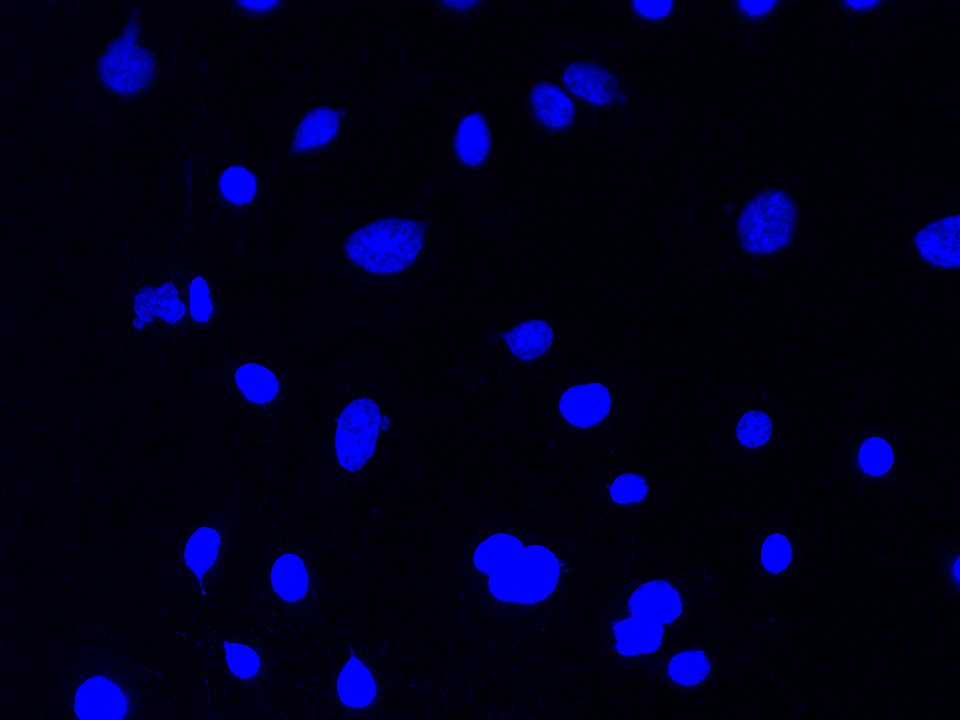

Supplement: Supplementary file 11 — Source data Fig. 5 [file 44321_2025_341_MOESM11_ESM.zip › Figure 5/5B&5D/Immunofluorescence_KO PM DAPI.tif]

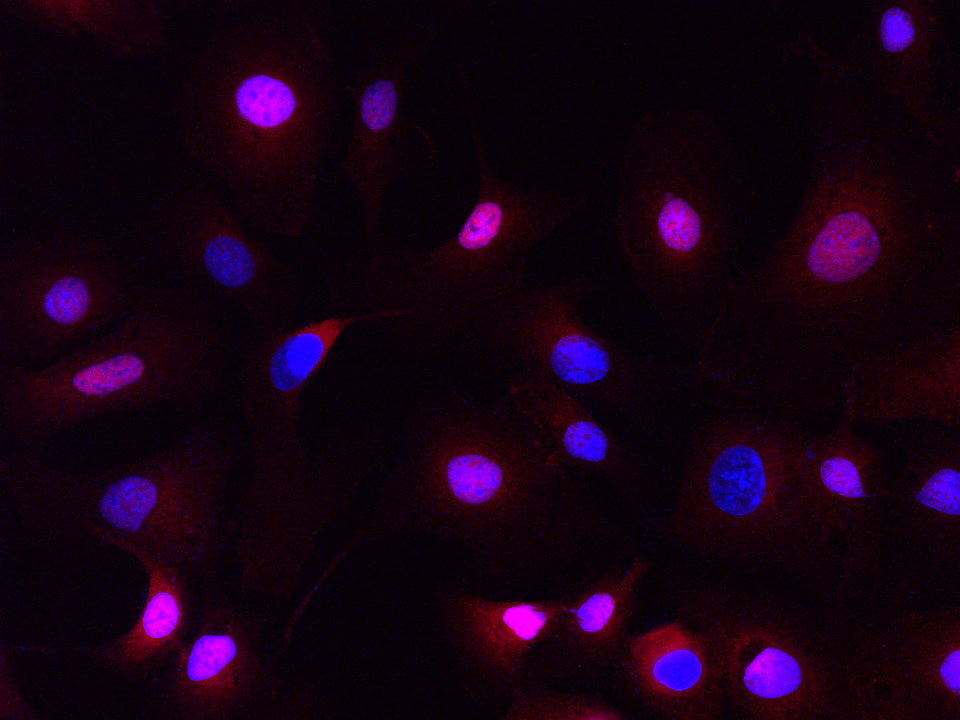

Supplement: Supplementary file 11 — Source data Fig. 5 [file 44321_2025_341_MOESM11_ESM.zip › Figure 5/5B&5D/Immunofluorescence_KO Control merge.tif]

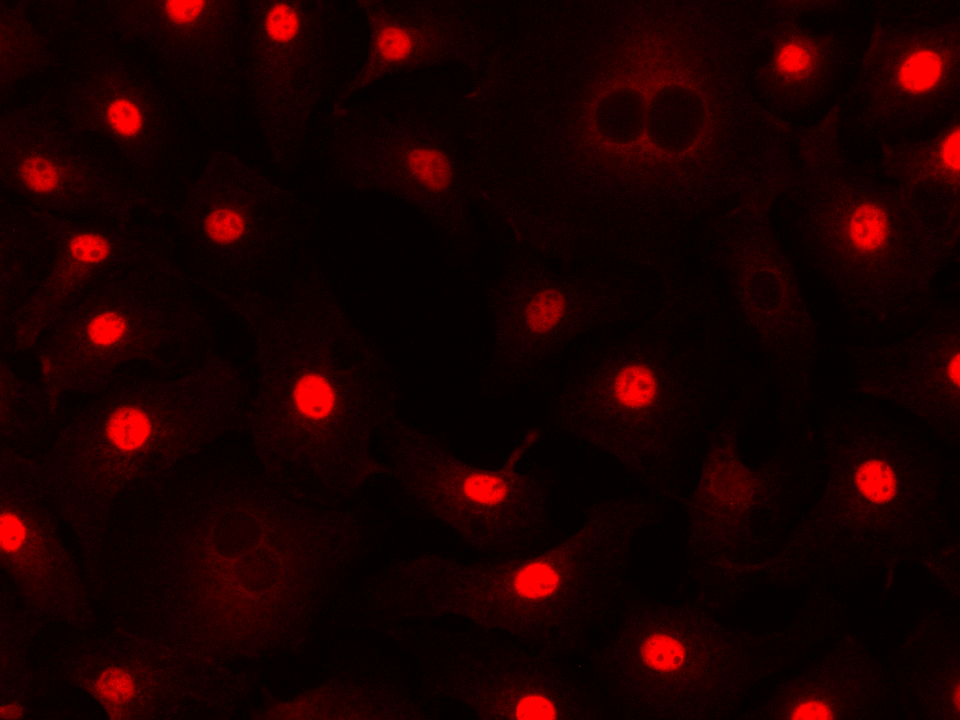

Supplement: Supplementary file 11 — Source data Fig. 5 [file 44321_2025_341_MOESM11_ESM.zip › Figure 5/5B&5D/Immunofluorescence_NC Control YAP.tif]

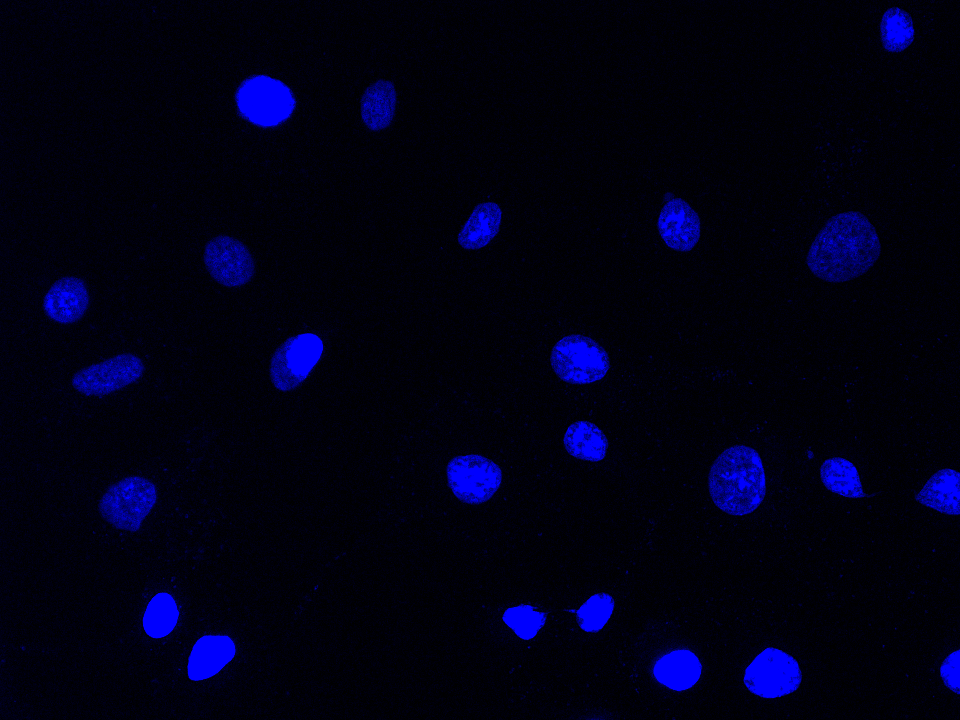

Supplement: Supplementary file 11 — Source data Fig. 5 [file 44321_2025_341_MOESM11_ESM.zip › Figure 5/5B&5D/Immunofluorescence_KO Control DAPI.tif]

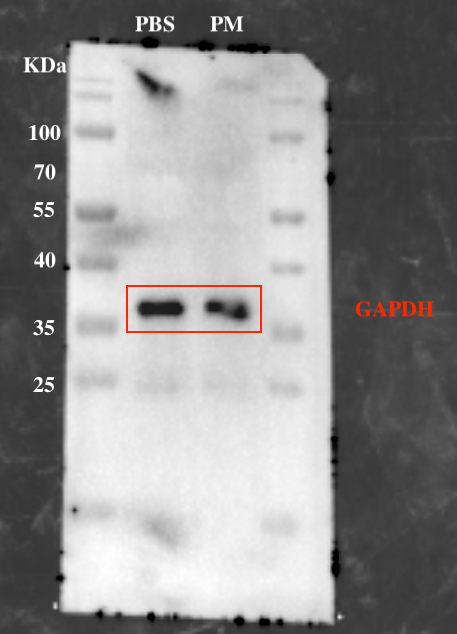

Supplement: Supplementary file 11 — Source data Fig. 5 [file 44321_2025_341_MOESM11_ESM.zip › Figure 5/5E/WB GAPDH.tif]

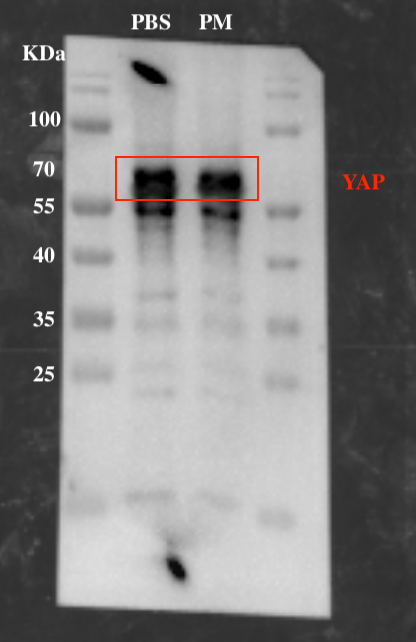

Supplement: Supplementary file 11 — Source data Fig. 5 [file 44321_2025_341_MOESM11_ESM.zip › Figure 5/5E/WB YAP.tif]

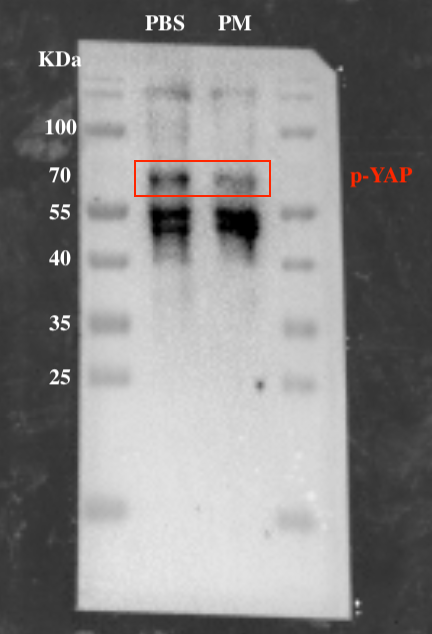

Supplement: Supplementary file 11 — Source data Fig. 5 [file 44321_2025_341_MOESM11_ESM.zip › Figure 5/5E/WB p-YAP.tif]

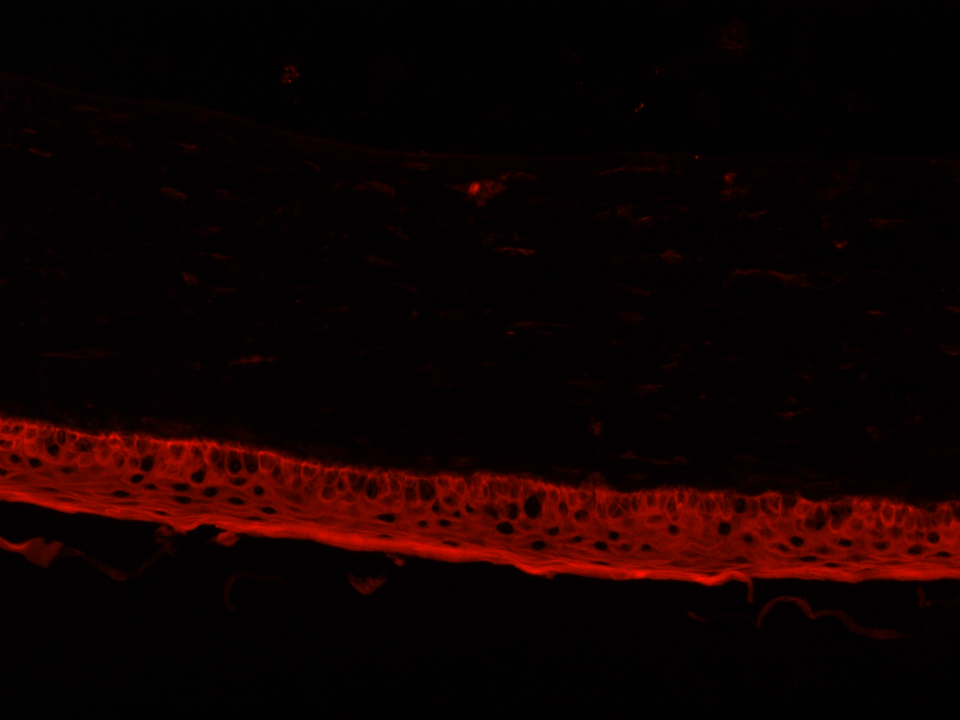

Supplement: Supplementary file 11 — Source data Fig. 5 [file 44321_2025_341_MOESM11_ESM.zip › Figure 5/5A&5C/Immunofluorescence_PBS YAP.tif]

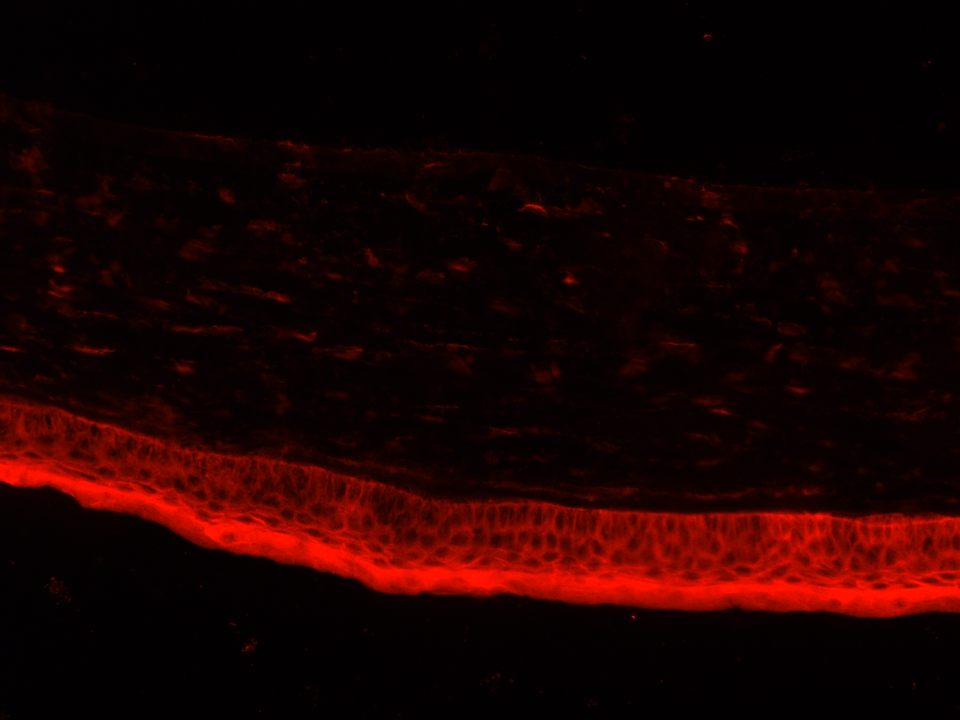

Supplement: Supplementary file 11 — Source data Fig. 5 [file 44321_2025_341_MOESM11_ESM.zip › Figure 5/5A&5C/Immunofluorescence_PM YAP.tif]

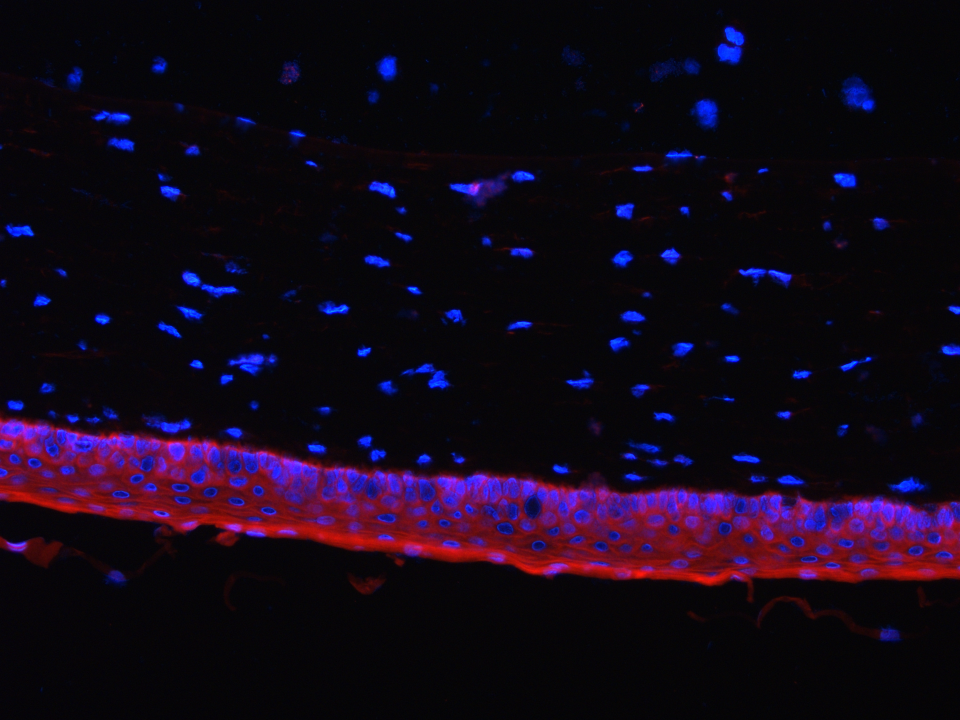

Supplement: Supplementary file 11 — Source data Fig. 5 [file 44321_2025_341_MOESM11_ESM.zip › Figure 5/5A&5C/Immunofluorescence_PBS merge.tif]

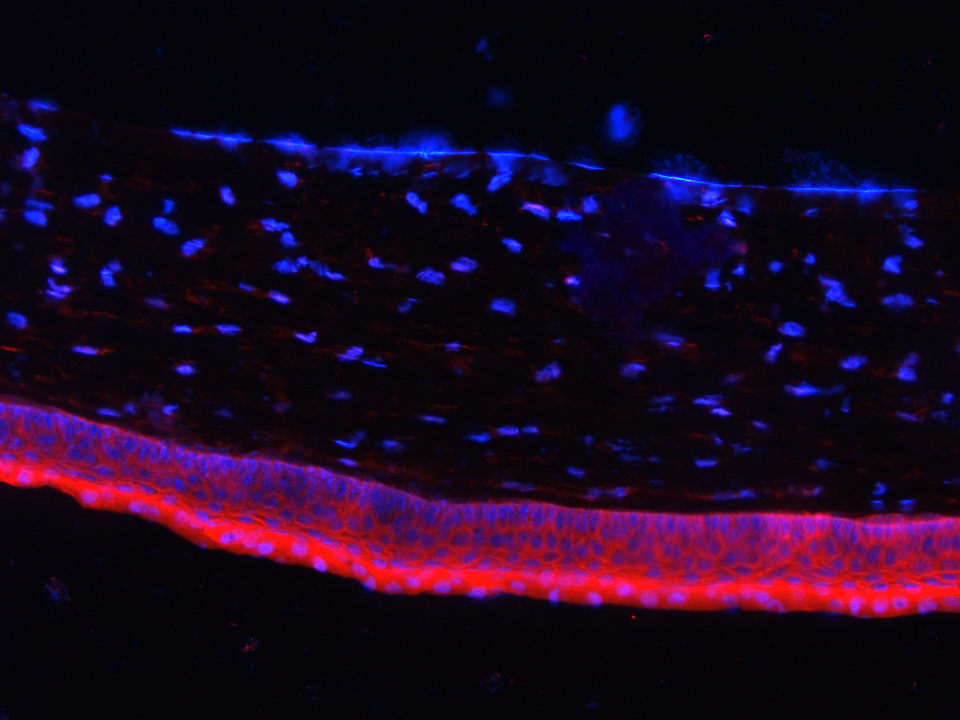

Supplement: Supplementary file 11 — Source data Fig. 5 [file 44321_2025_341_MOESM11_ESM.zip › Figure 5/5A&5C/Immunofluorescence_PM merge.tif]

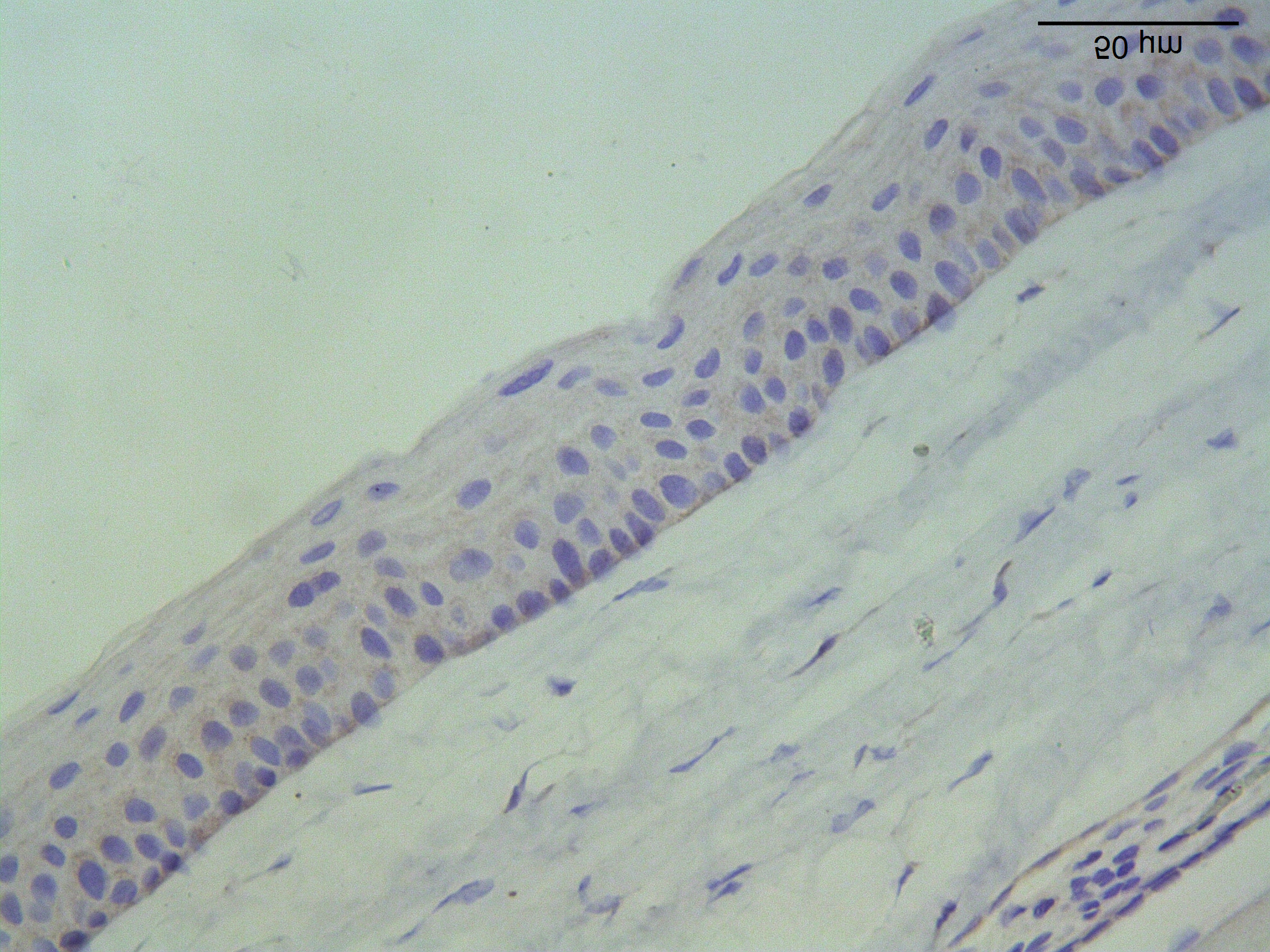

Supplement: Supplementary file 13 — Source data Fig. 7 [file 44321_2025_341_MOESM13_ESM.zip › Figure 7/7D/Immunohistochemistry_PBS.jpg]

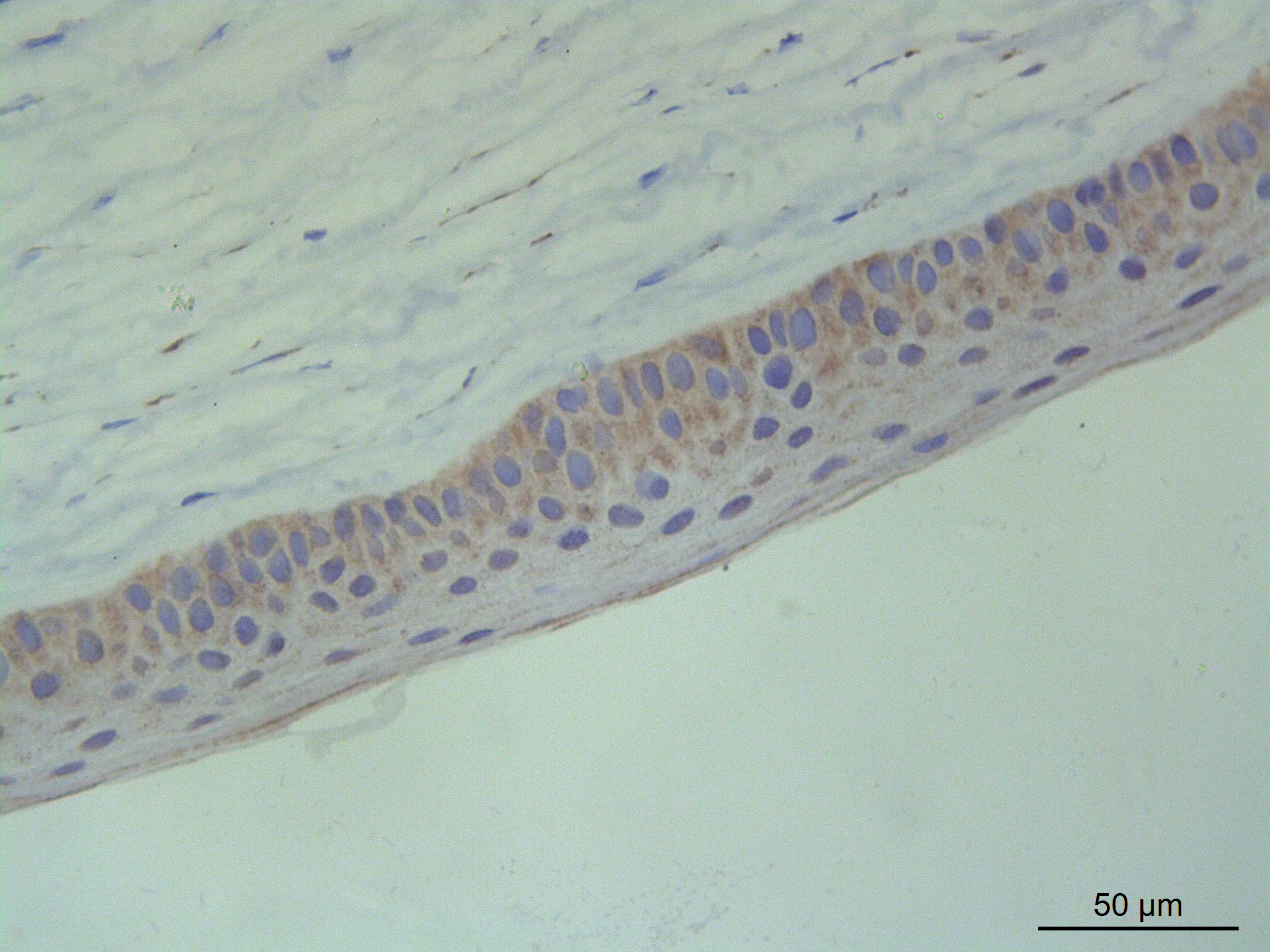

Supplement: Supplementary file 13 — Source data Fig. 7 [file 44321_2025_341_MOESM13_ESM.zip › Figure 7/7D/Immunohistochemistry_PM+LNP-siPAI-2.jpg]

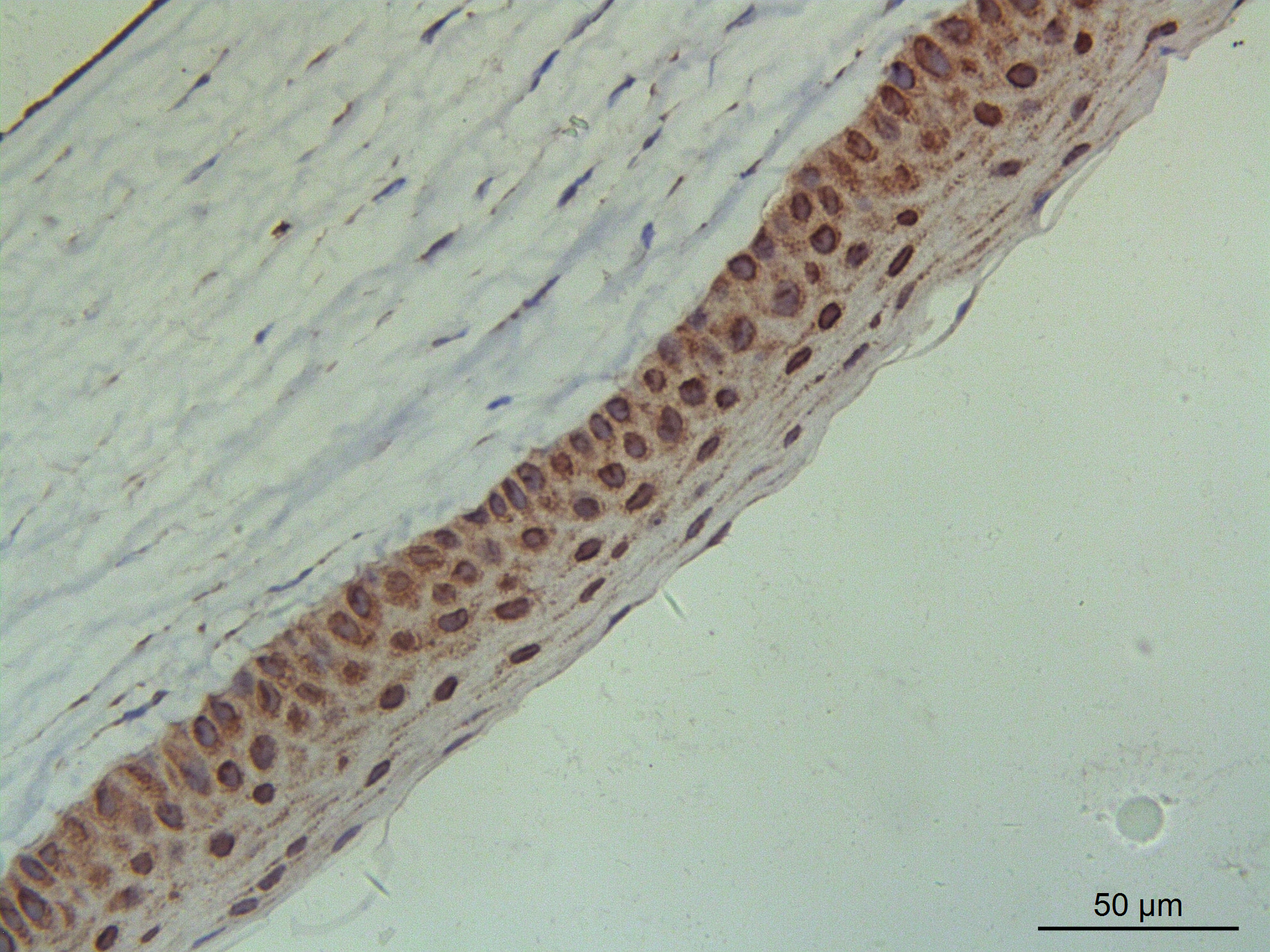

Supplement: Supplementary file 13 — Source data Fig. 7 [file 44321_2025_341_MOESM13_ESM.zip › Figure 7/7D/Immunohistochemistry_PM+LNP.jpg]

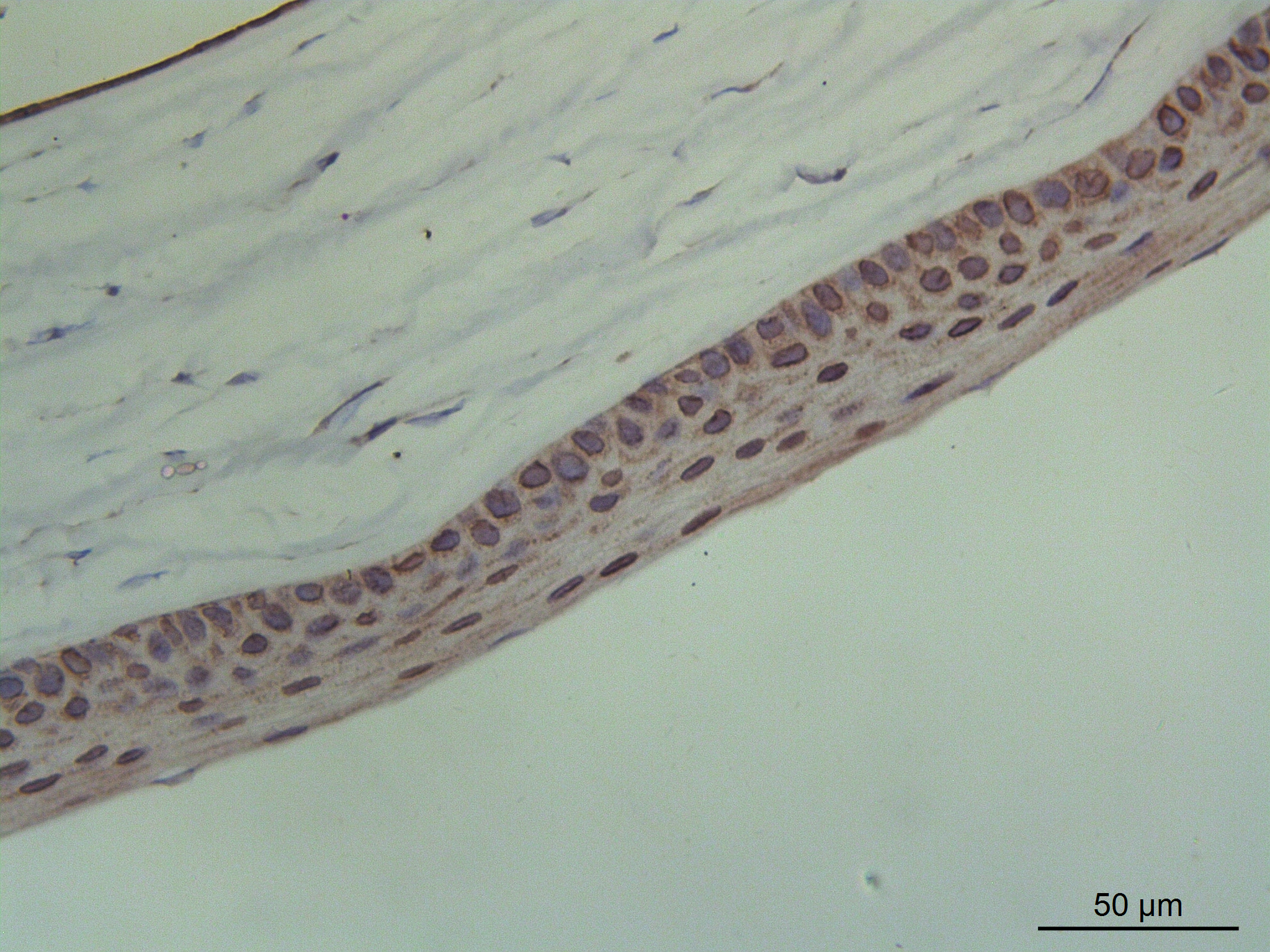

Supplement: Supplementary file 13 — Source data Fig. 7 [file 44321_2025_341_MOESM13_ESM.zip › Figure 7/7D/Immunohistochemistry_PM.jpg]

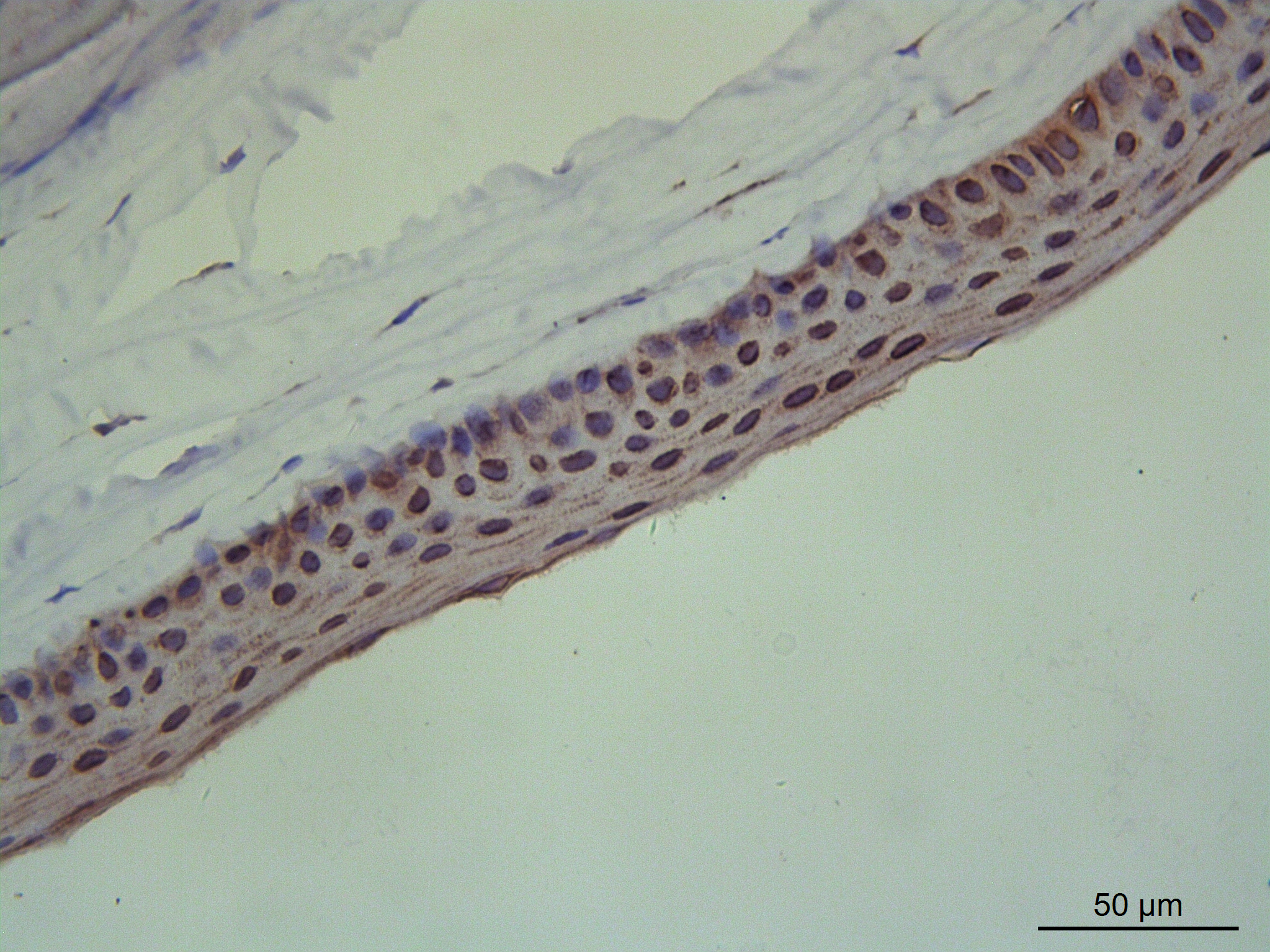

Supplement: Supplementary file 13 — Source data Fig. 7 [file 44321_2025_341_MOESM13_ESM.zip › Figure 7/7D/Immunohistochemistry_PM+LNP-siNC.jpg]

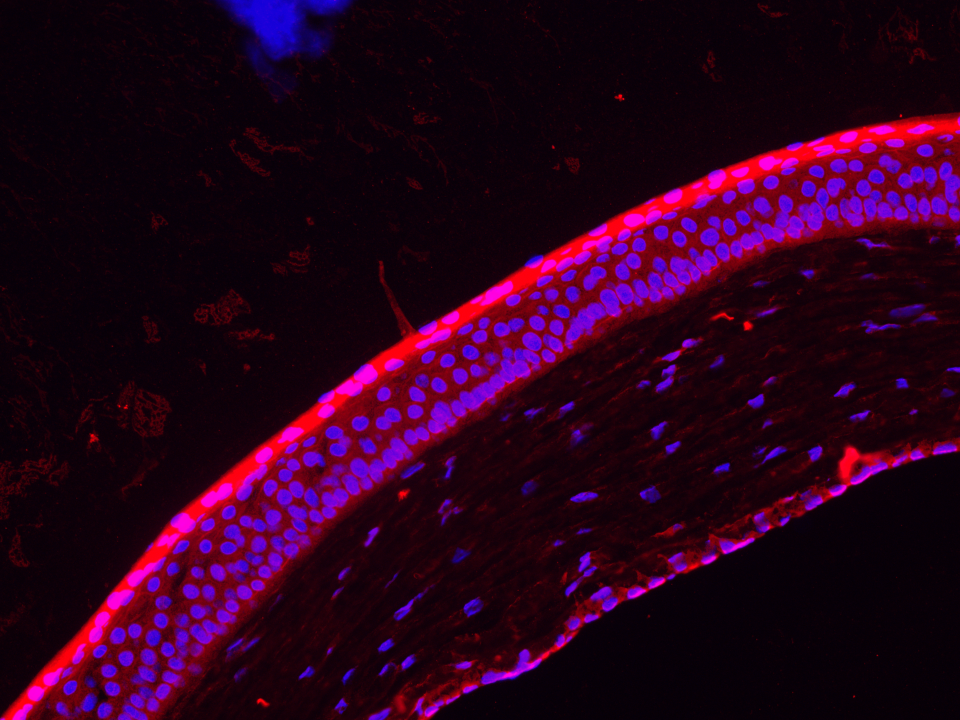

Supplement: Supplementary file 13 — Source data Fig. 7 [file 44321_2025_341_MOESM13_ESM.zip › Figure 7/7C/Immunofluorescence_LC3B_PM+LNP.tif]

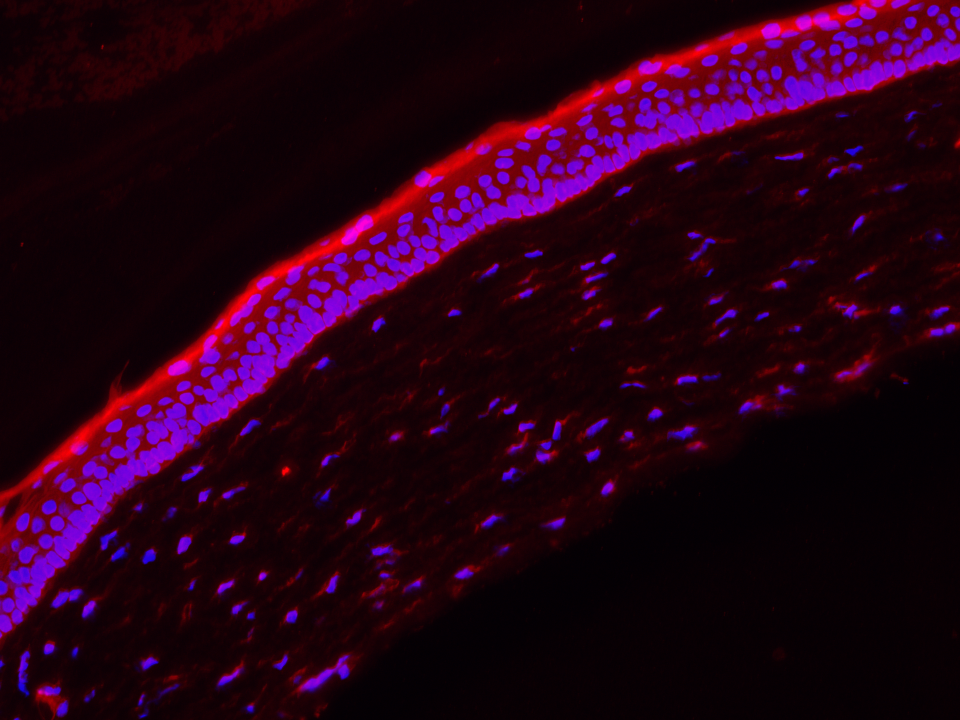

Supplement: Supplementary file 13 — Source data Fig. 7 [file 44321_2025_341_MOESM13_ESM.zip › Figure 7/7C/Immunofluorescence_LC3B_PM+LNP-siNC.tif]

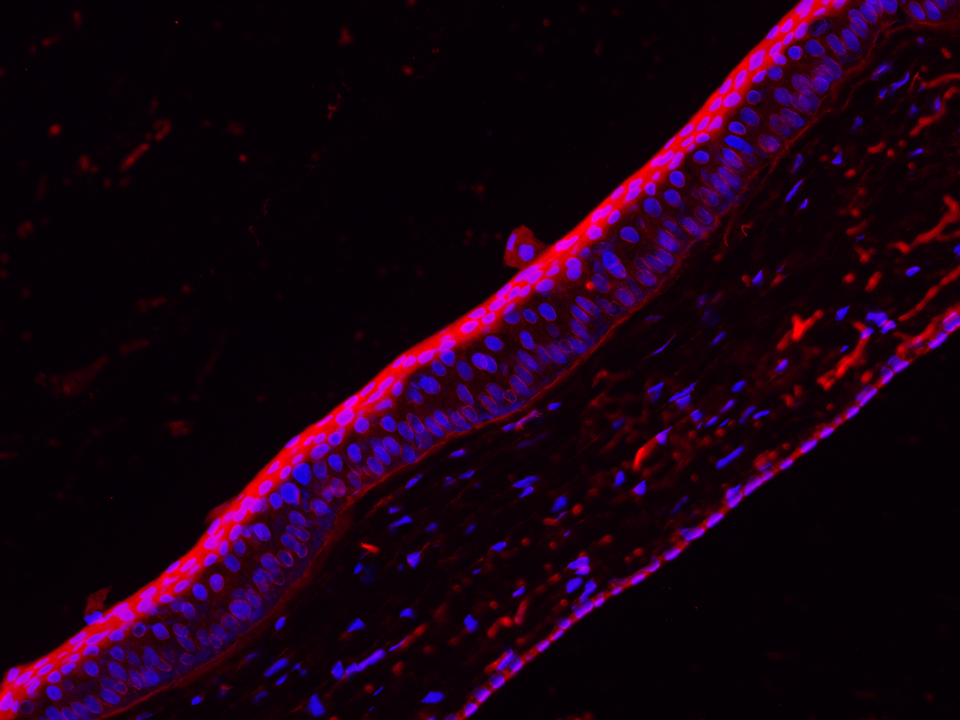

Supplement: Supplementary file 13 — Source data Fig. 7 [file 44321_2025_341_MOESM13_ESM.zip › Figure 7/7C/Immunofluorescence_LC3B_PBS.tif]

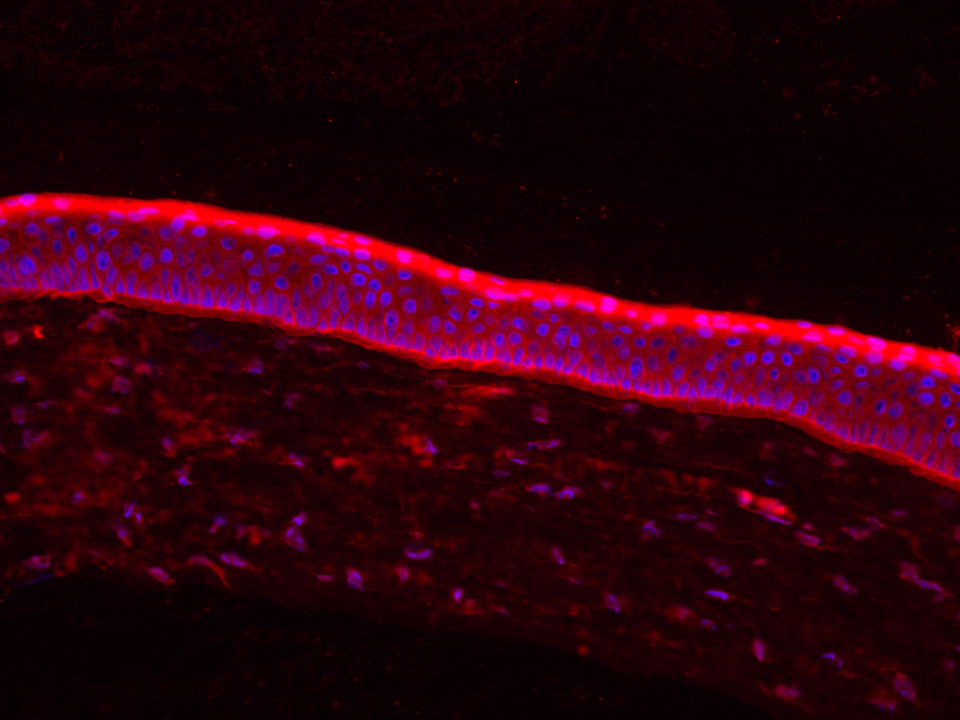

Supplement: Supplementary file 13 — Source data Fig. 7 [file 44321_2025_341_MOESM13_ESM.zip › Figure 7/7C/Immunofluorescence_LC3B_PM.tif]

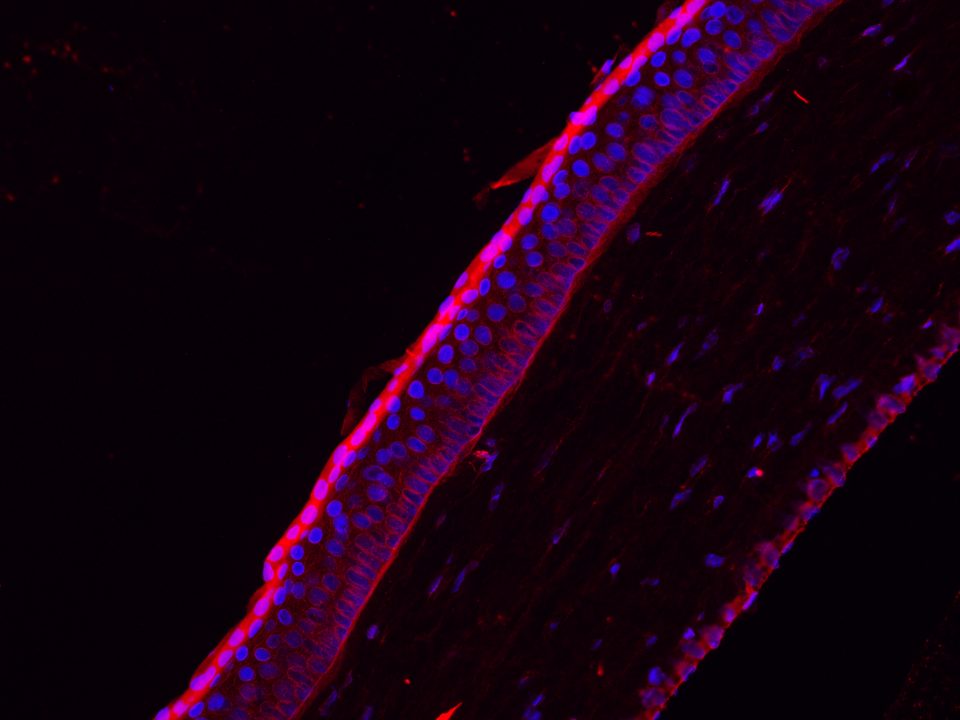

Supplement: Supplementary file 13 — Source data Fig. 7 [file 44321_2025_341_MOESM13_ESM.zip › Figure 7/7C/Immunofluorescence_LC3B_PM+LNP-siPAI-2.tif]

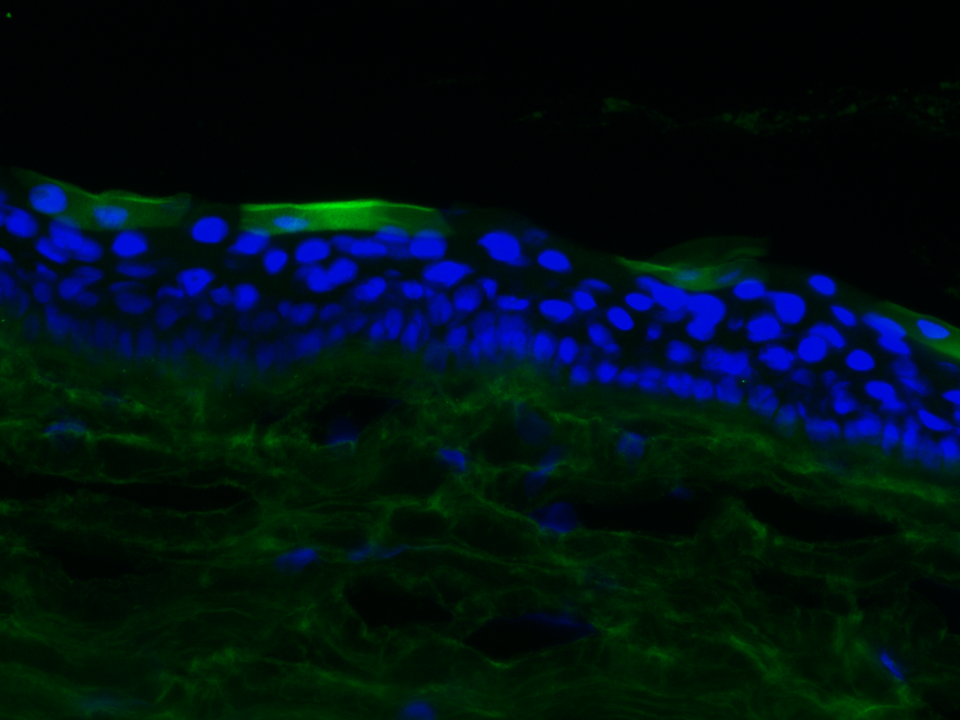

Supplement: Supplementary file 13 — Source data Fig. 7 [file 44321_2025_341_MOESM13_ESM.zip › Figure 7/7C/Immunofluorescence_PAI-2_PM+LNP-siNC.tif]

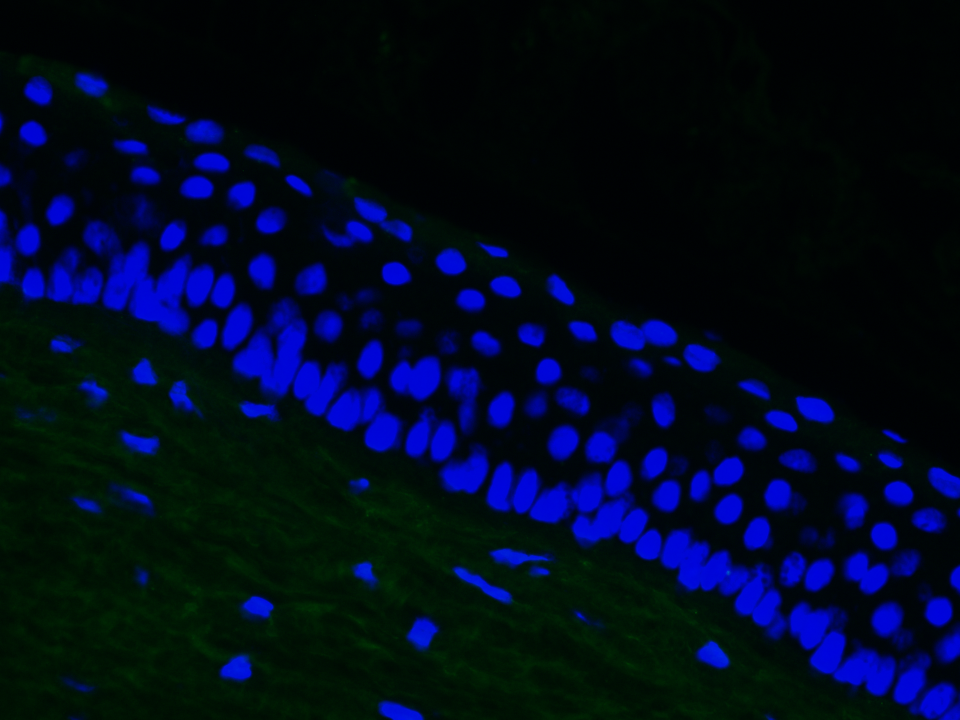

Supplement: Supplementary file 13 — Source data Fig. 7 [file 44321_2025_341_MOESM13_ESM.zip › Figure 7/7C/Immunofluorescence_PAI-2_PM+LNP-siPAI-2.tif]

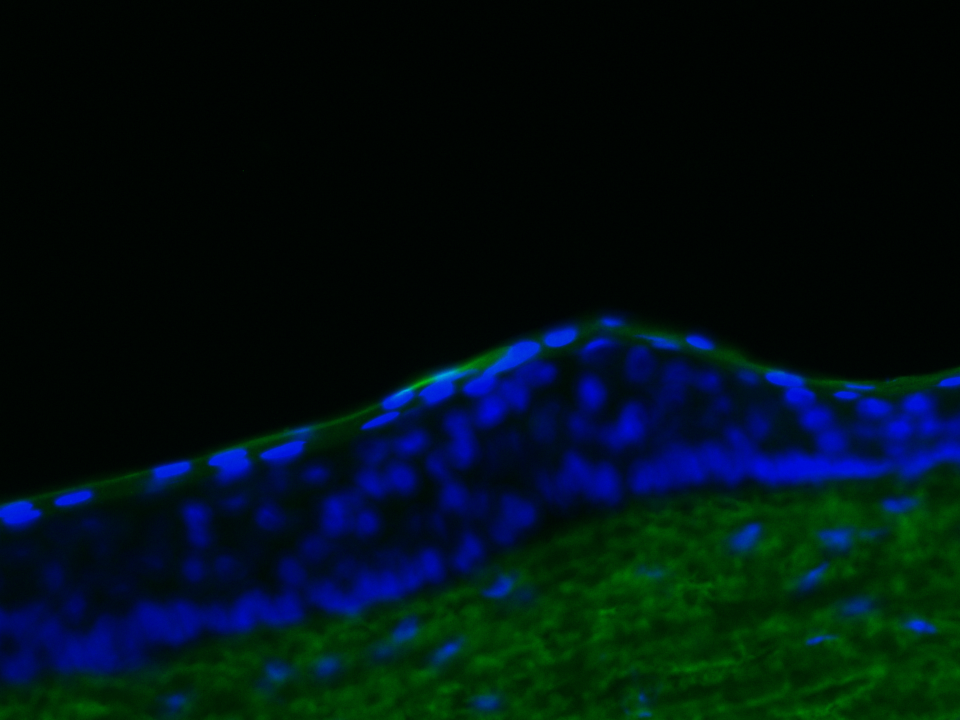

Supplement: Supplementary file 13 — Source data Fig. 7 [file 44321_2025_341_MOESM13_ESM.zip › Figure 7/7C/Immunofluorescence_PAI-2_PM.tif]
