# Supplementary figures and images for: Corneal biomechanical cues mediated by PAI-2: the origin of PM2.5-induced corneal disease (part 2 of 2)
Source: EMBO Mol Med. 2025 Dec 1;18(1):120–50. doi: 10.1038/s44321-025-00341-0 (PMC12808792; doi:10.1038/s44321-025-00341-0)

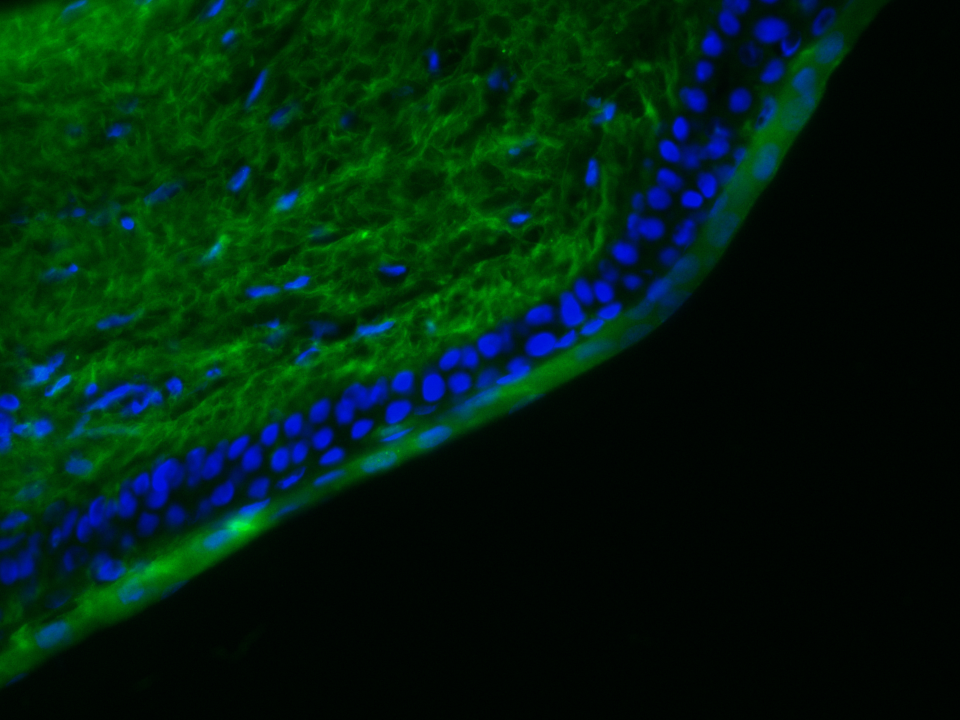

Supplement: Supplementary file 13 — Source data Fig. 7 [file 44321_2025_341_MOESM13_ESM.zip › Figure 7/7C/Immunofluorescence_PAI-2_PM+LNP.tif]

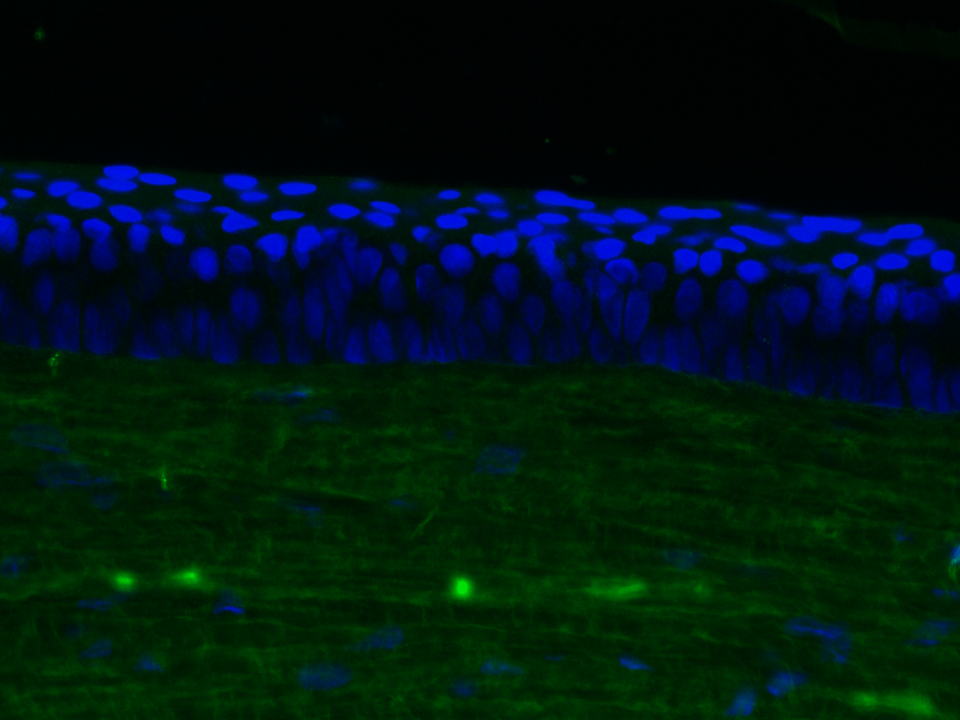

Supplement: Supplementary file 13 — Source data Fig. 7 [file 44321_2025_341_MOESM13_ESM.zip › Figure 7/7C/Immunofluorescence_PAI-2_PBS.tif]

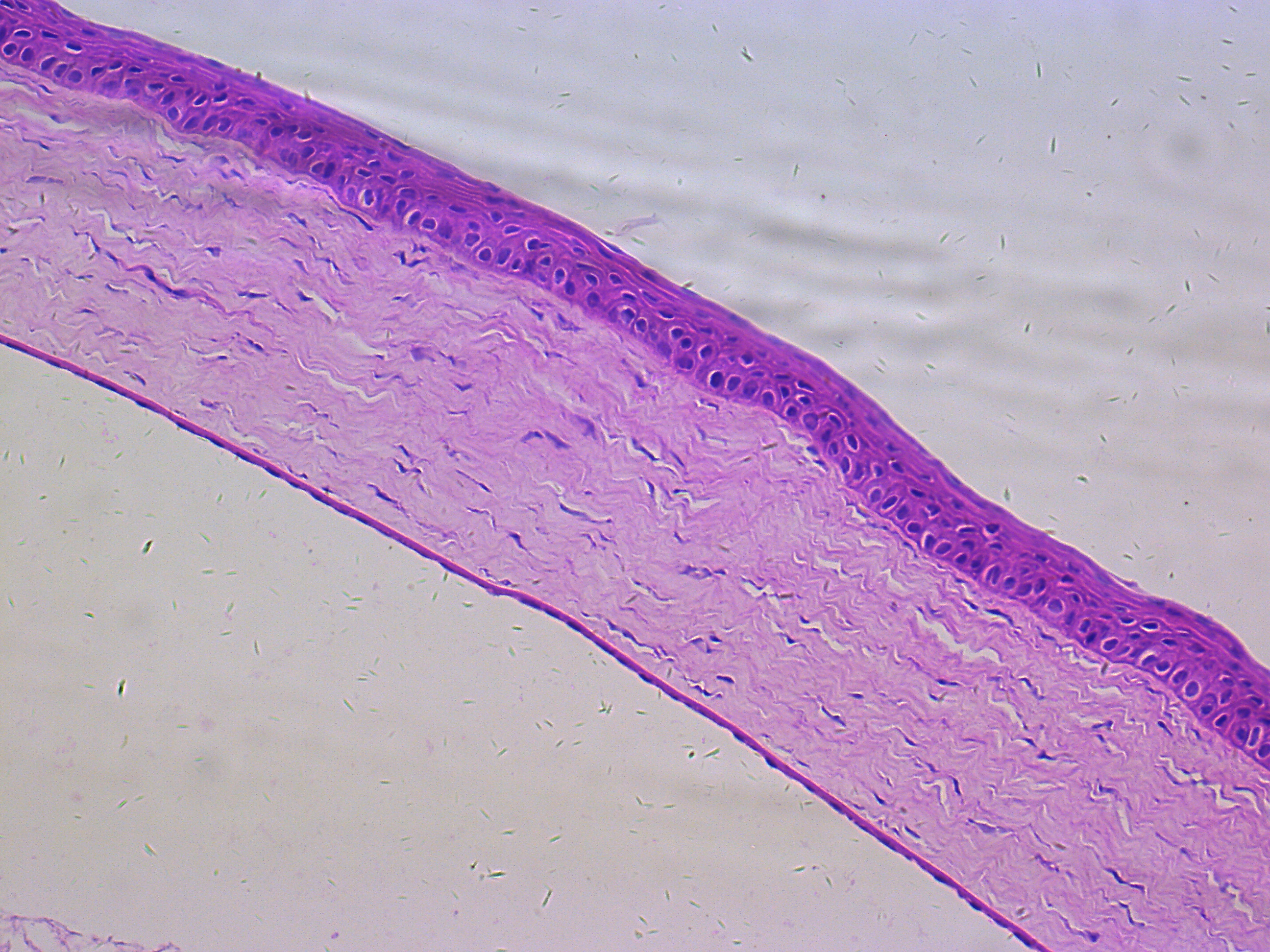

Supplement: Supplementary file 13 — Source data Fig. 7 [file 44321_2025_341_MOESM13_ESM.zip › Figure 7/7B/HE staining_Cornea PM+LNP-siNC.tif]

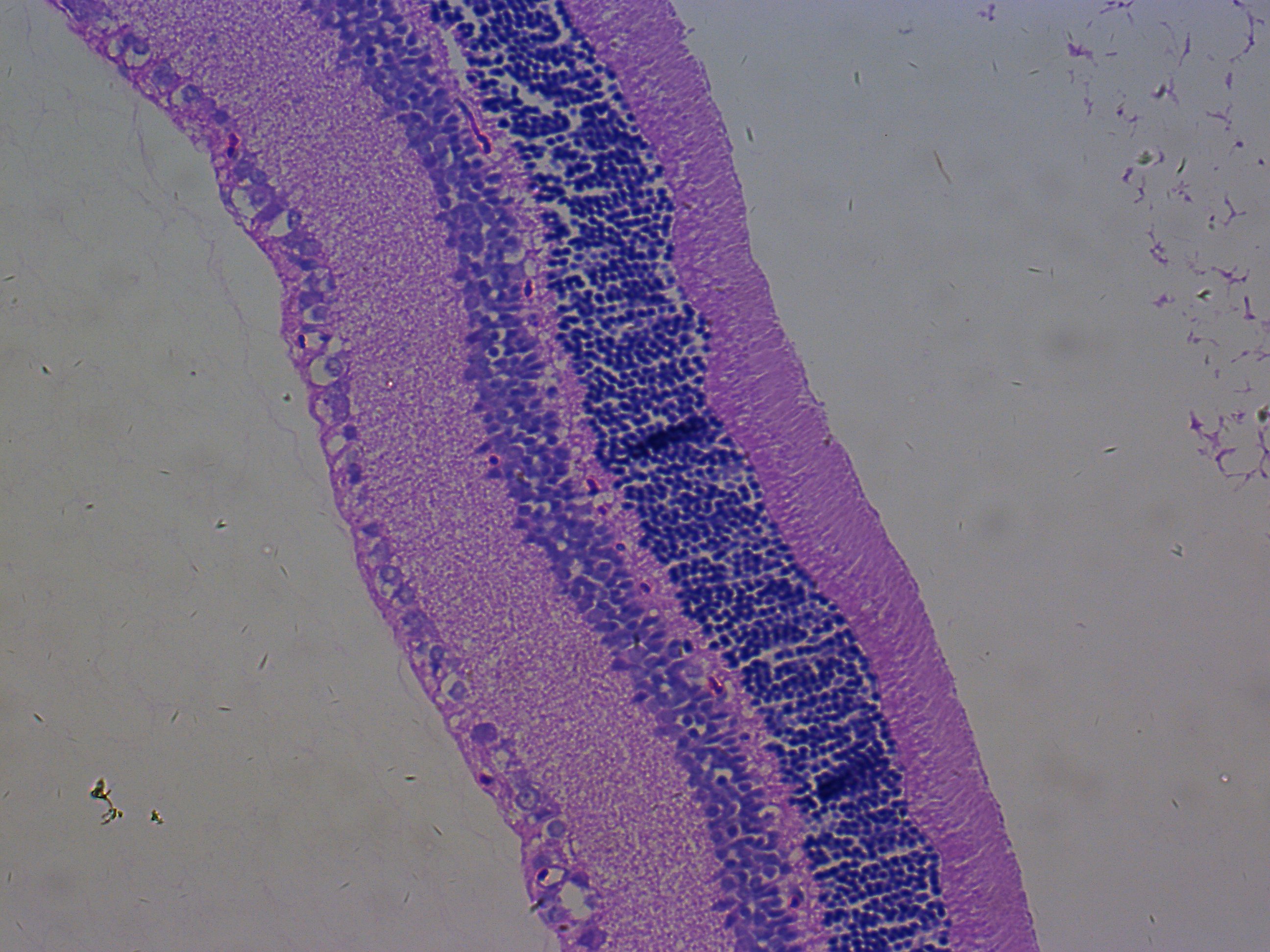

Supplement: Supplementary file 13 — Source data Fig. 7 [file 44321_2025_341_MOESM13_ESM.zip › Figure 7/7B/HE staining_Retina PM+LNP-siPAI-2.tif]

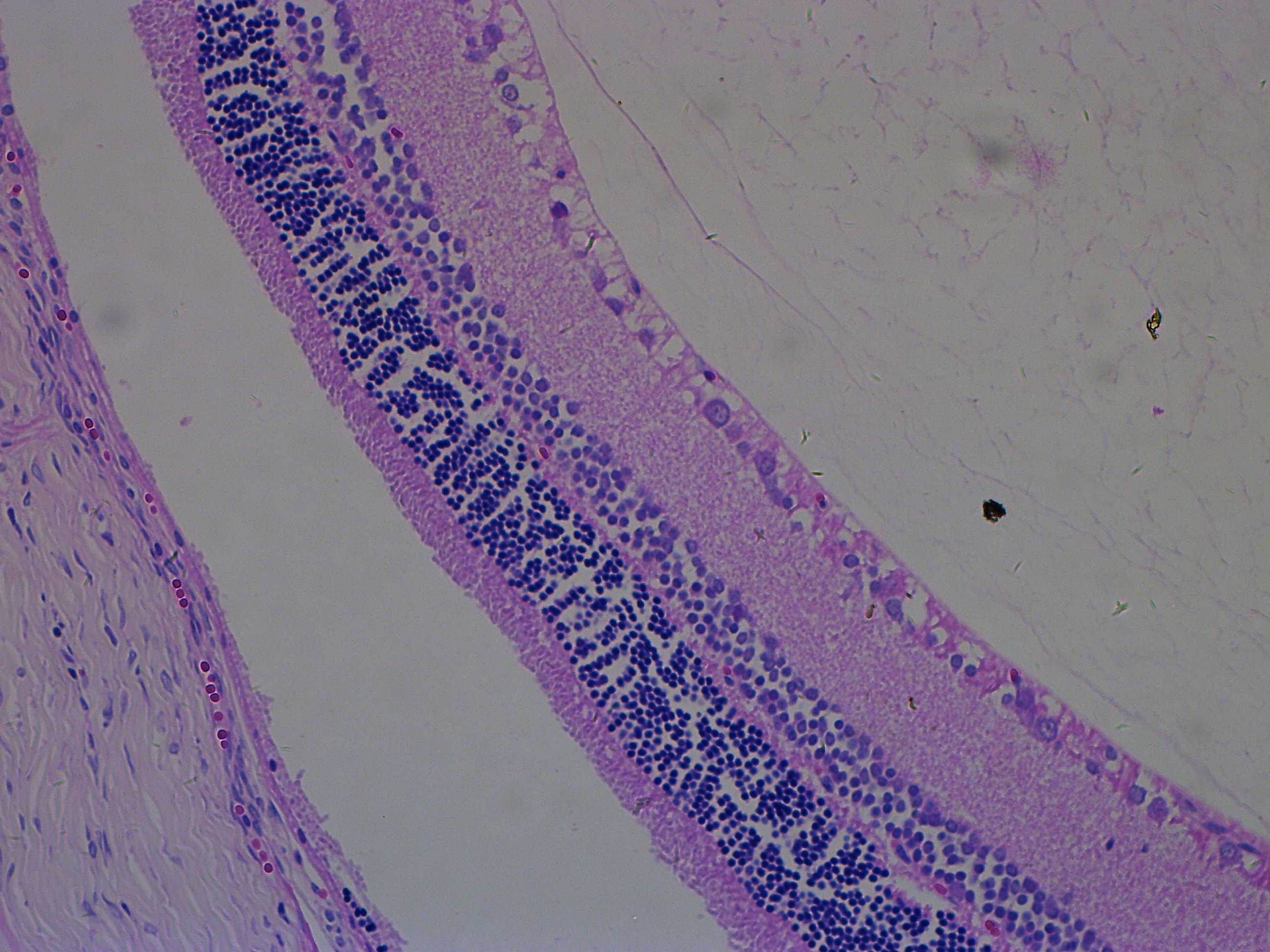

Supplement: Supplementary file 13 — Source data Fig. 7 [file 44321_2025_341_MOESM13_ESM.zip › Figure 7/7B/HE staining_Retina PM.tif]

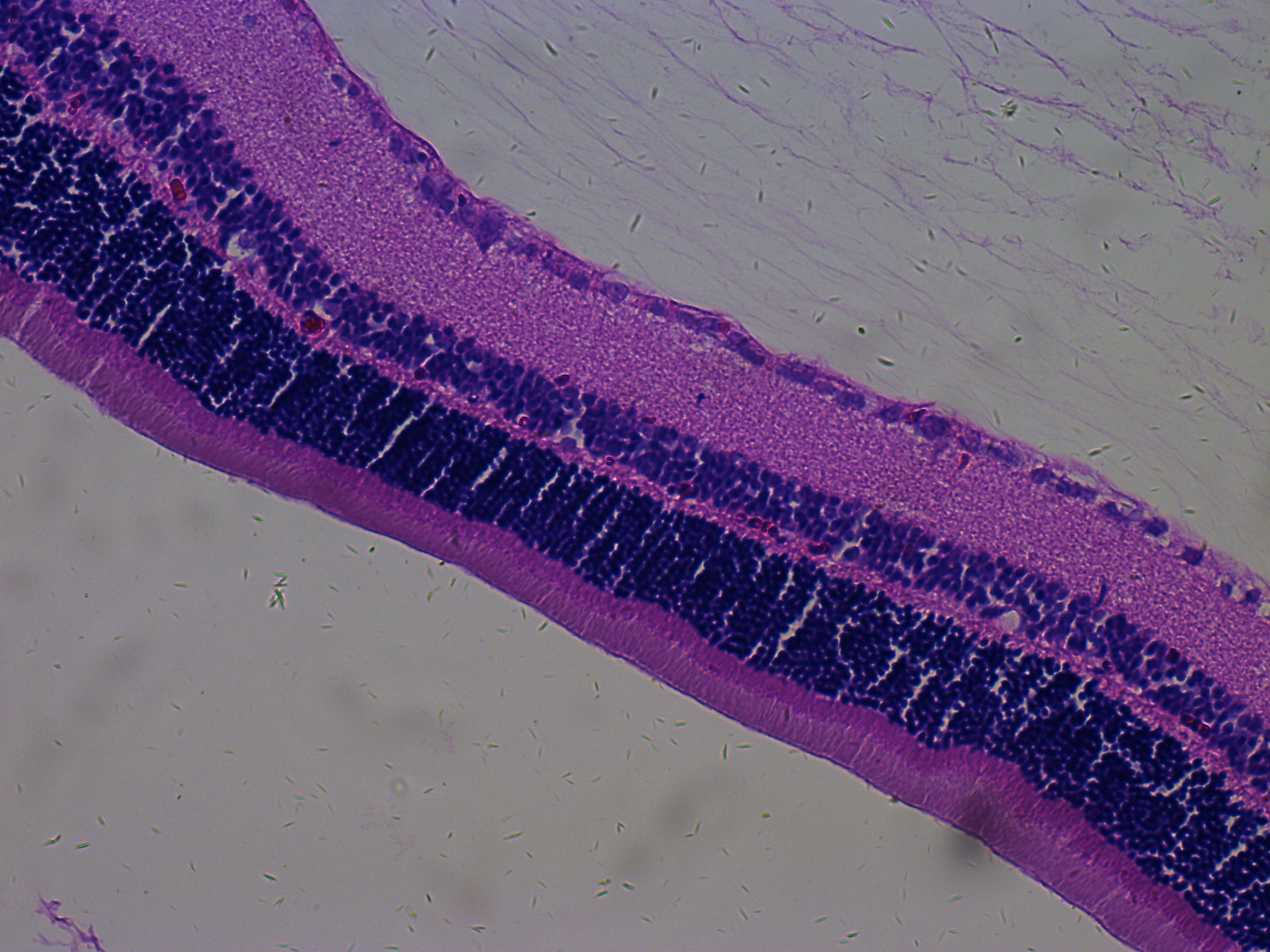

Supplement: Supplementary file 13 — Source data Fig. 7 [file 44321_2025_341_MOESM13_ESM.zip › Figure 7/7B/HE staining_Retina PM+LNP-siNC.tif]

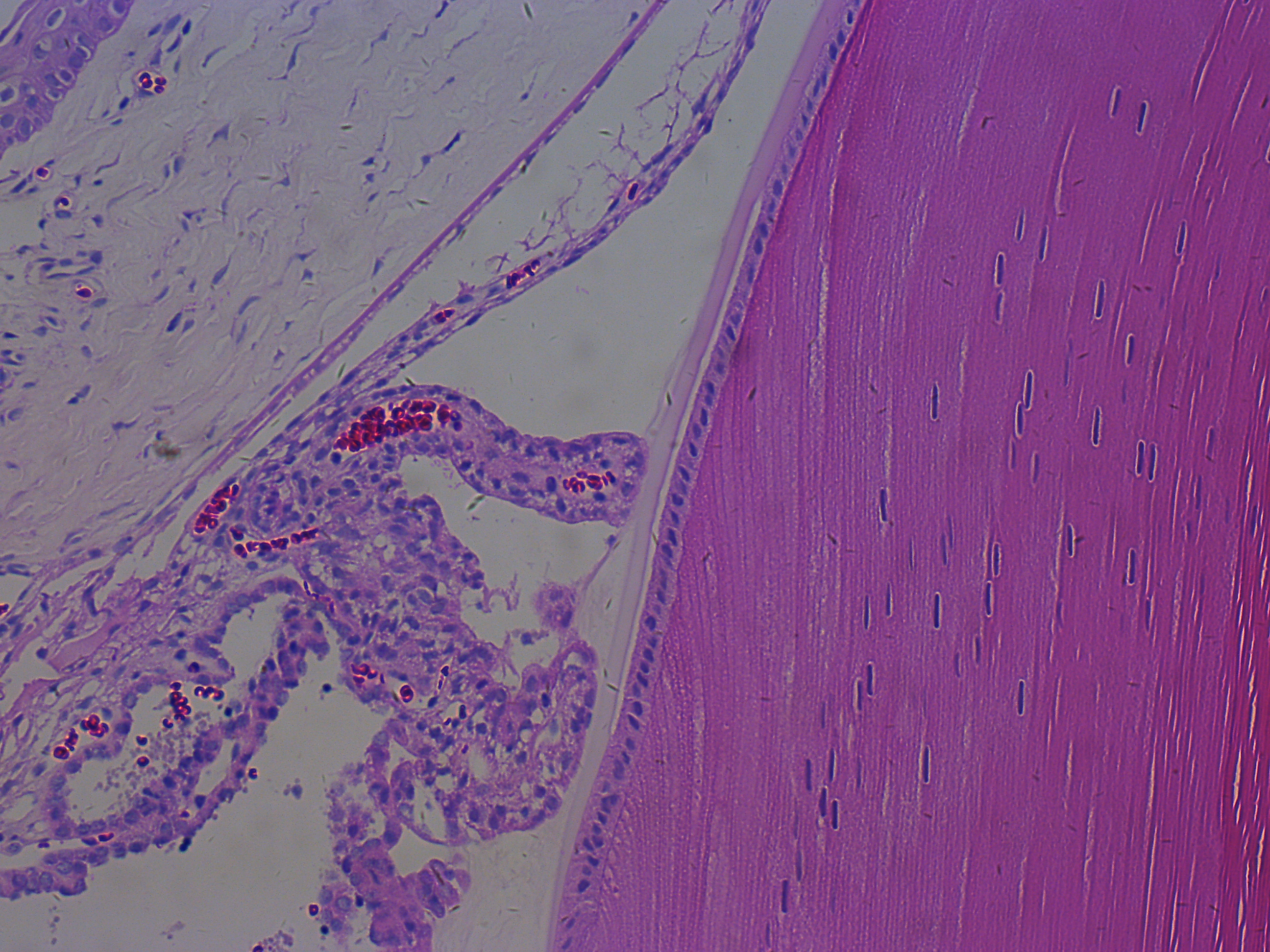

Supplement: Supplementary file 13 — Source data Fig. 7 [file 44321_2025_341_MOESM13_ESM.zip › Figure 7/7B/HE staining_Lens PBS.tif]

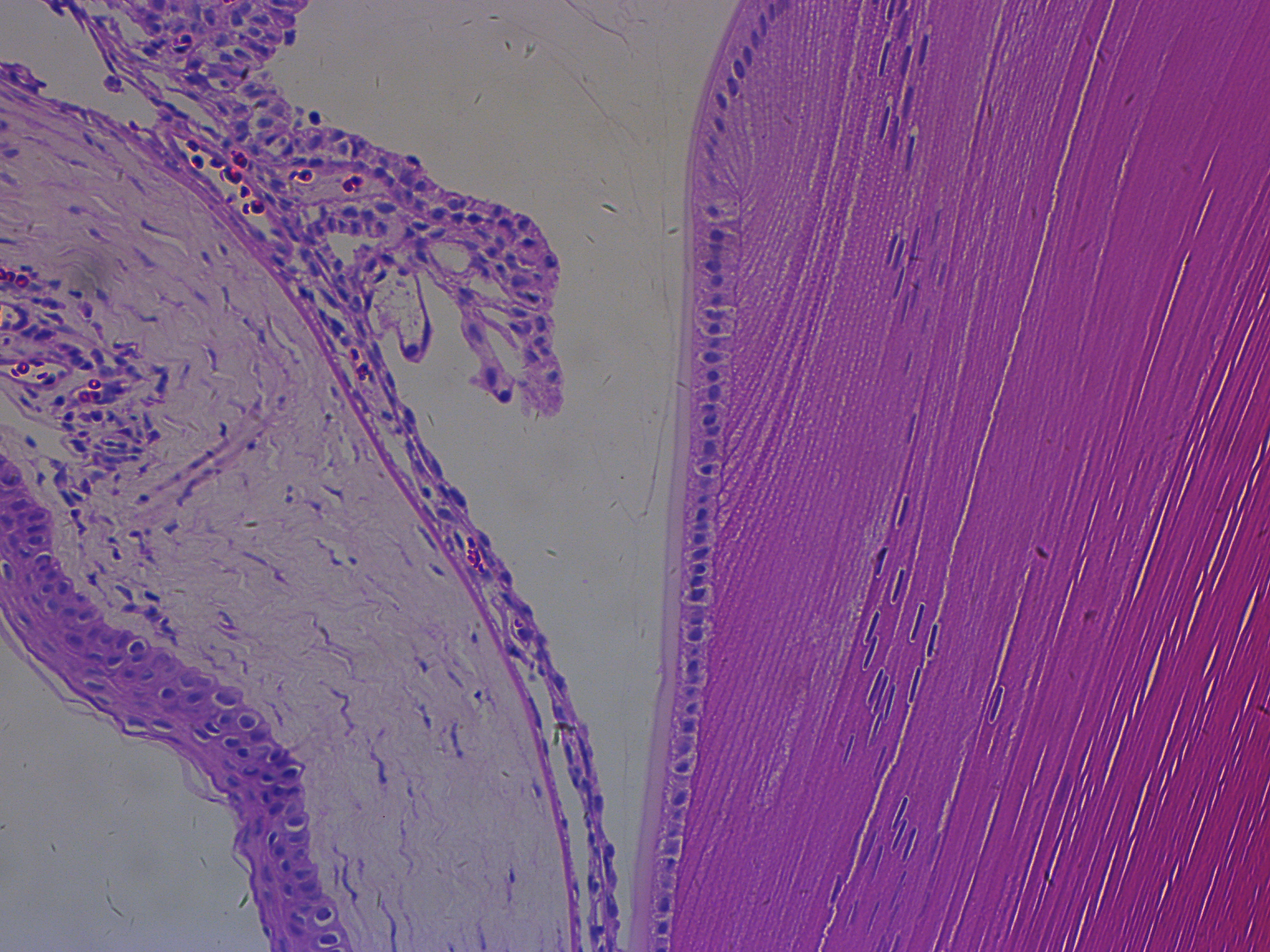

Supplement: Supplementary file 13 — Source data Fig. 7 [file 44321_2025_341_MOESM13_ESM.zip › Figure 7/7B/HE staining_Lens PM+LNP.tif]

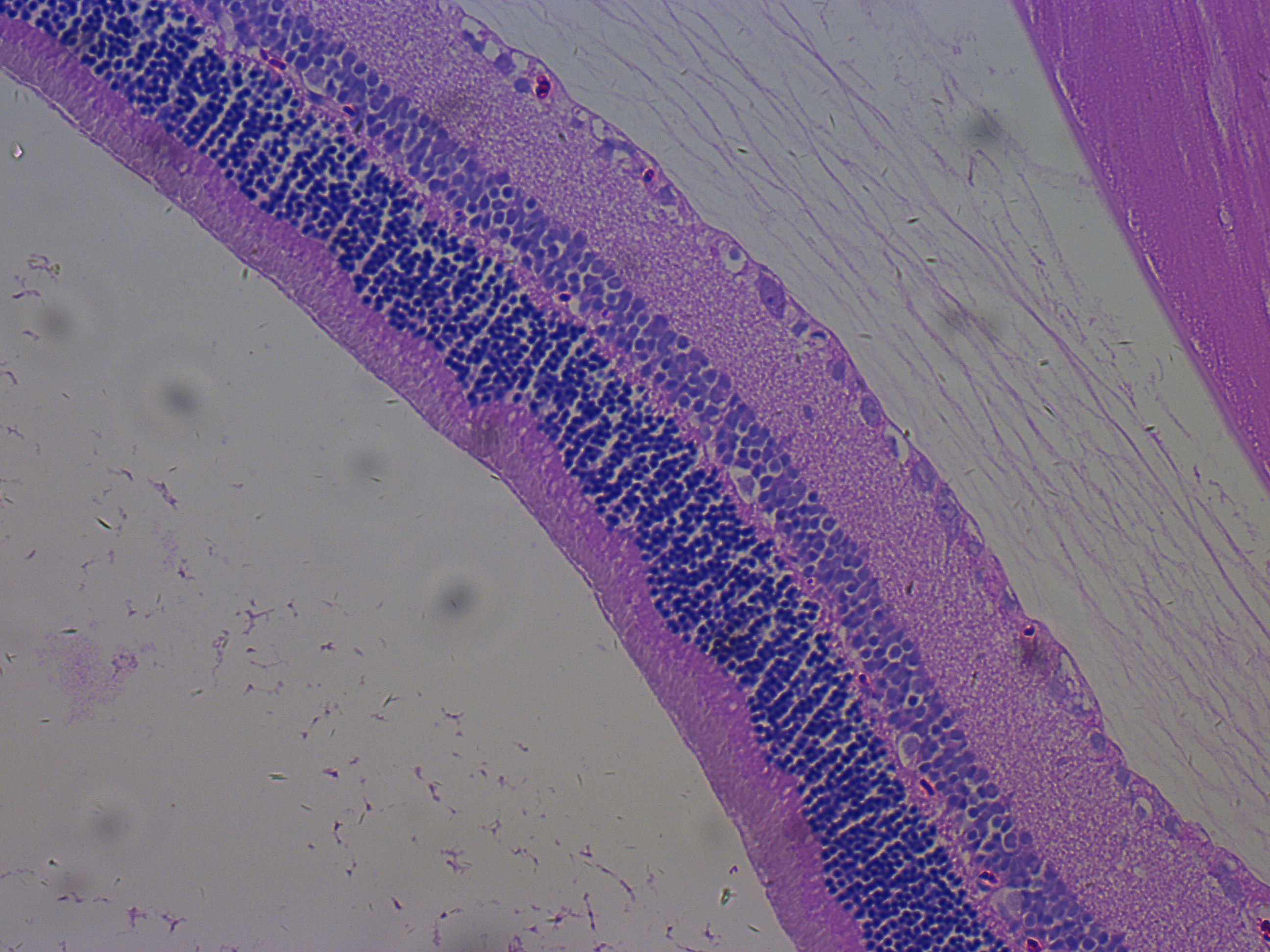

Supplement: Supplementary file 13 — Source data Fig. 7 [file 44321_2025_341_MOESM13_ESM.zip › Figure 7/7B/HE staining_Retina PM+LNP.tif]

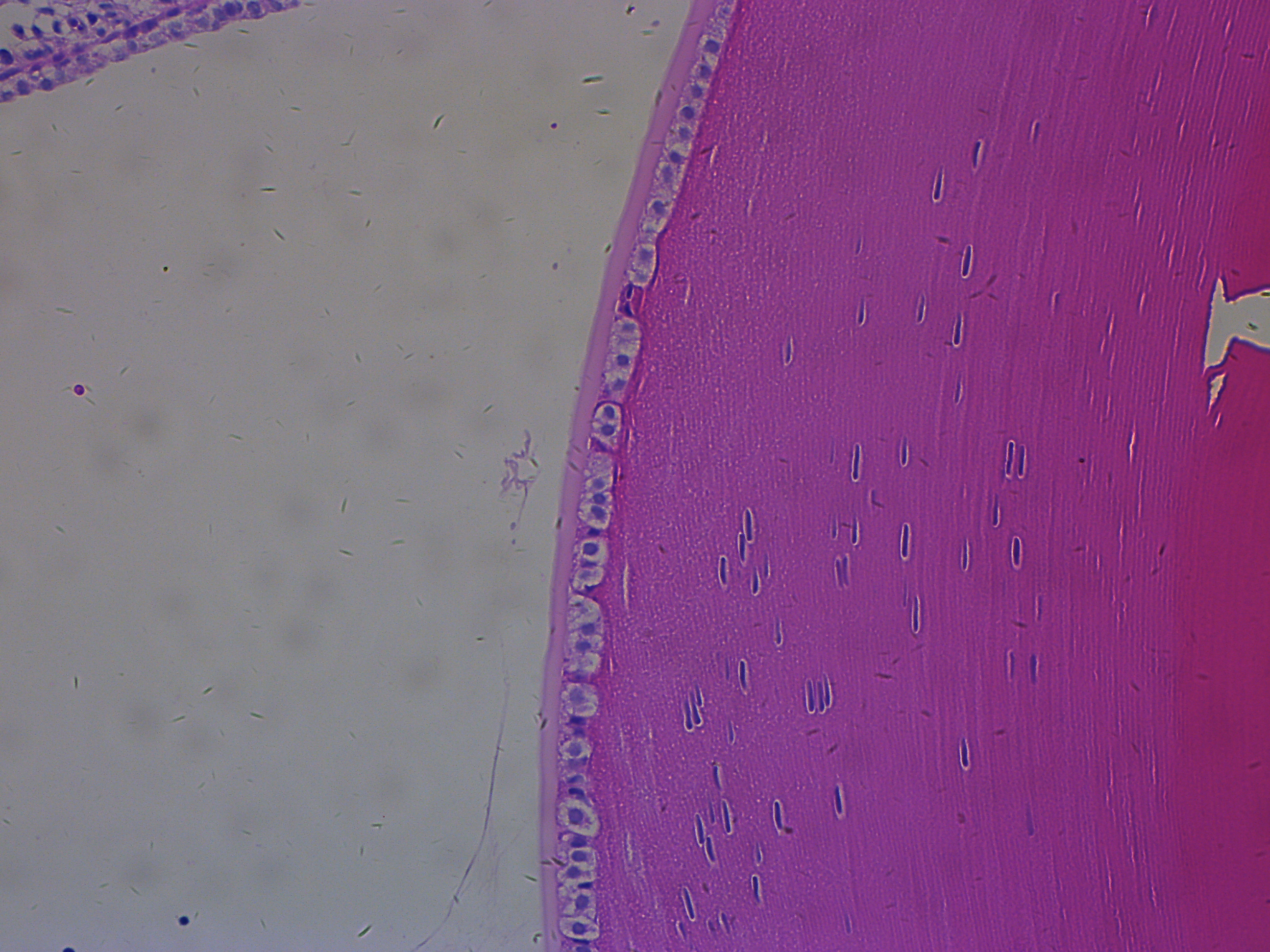

Supplement: Supplementary file 13 — Source data Fig. 7 [file 44321_2025_341_MOESM13_ESM.zip › Figure 7/7B/HE staining_Lens PM.tif]

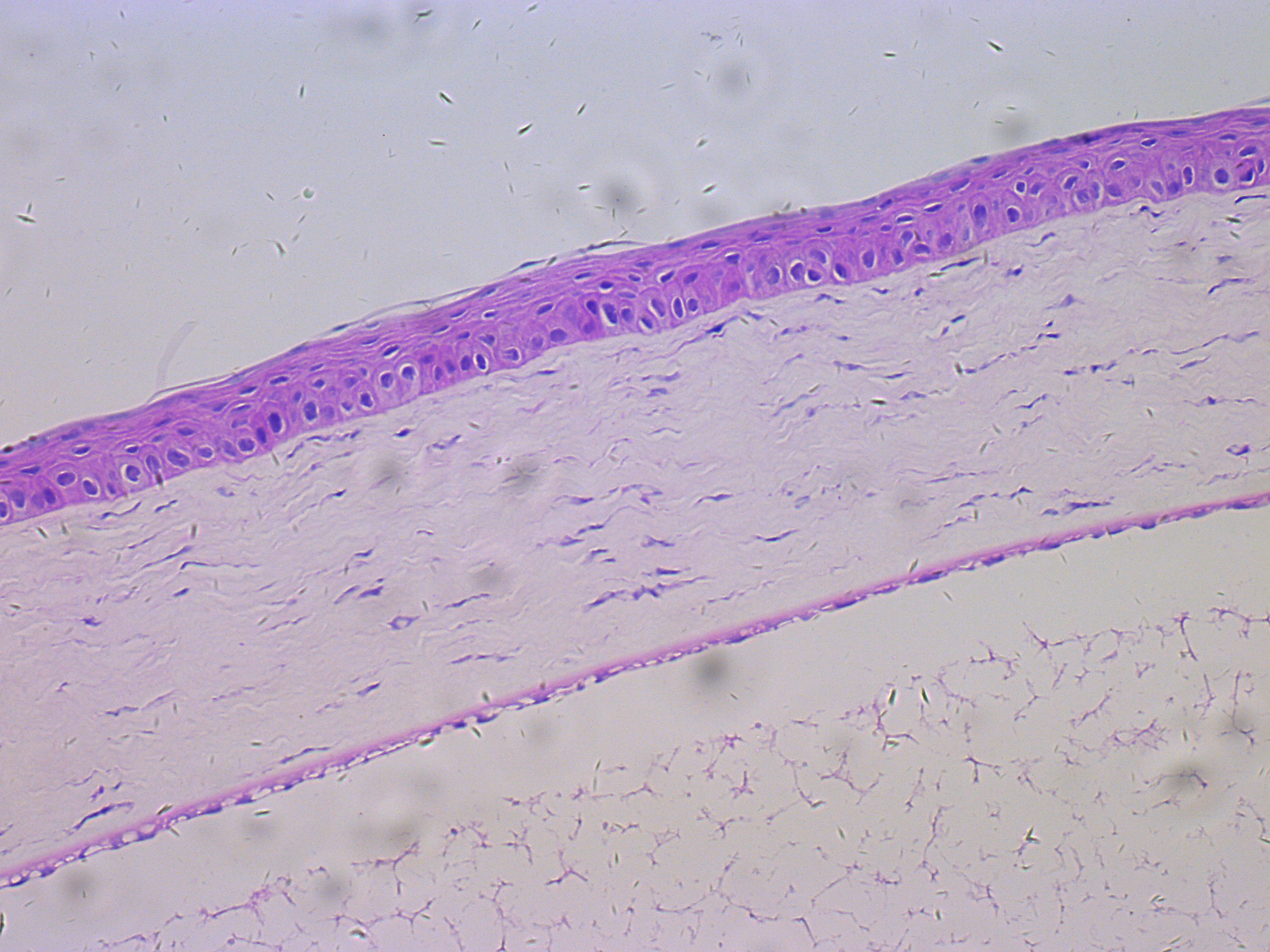

Supplement: Supplementary file 13 — Source data Fig. 7 [file 44321_2025_341_MOESM13_ESM.zip › Figure 7/7B/HE staining_Cornea PM+LNP.tif]

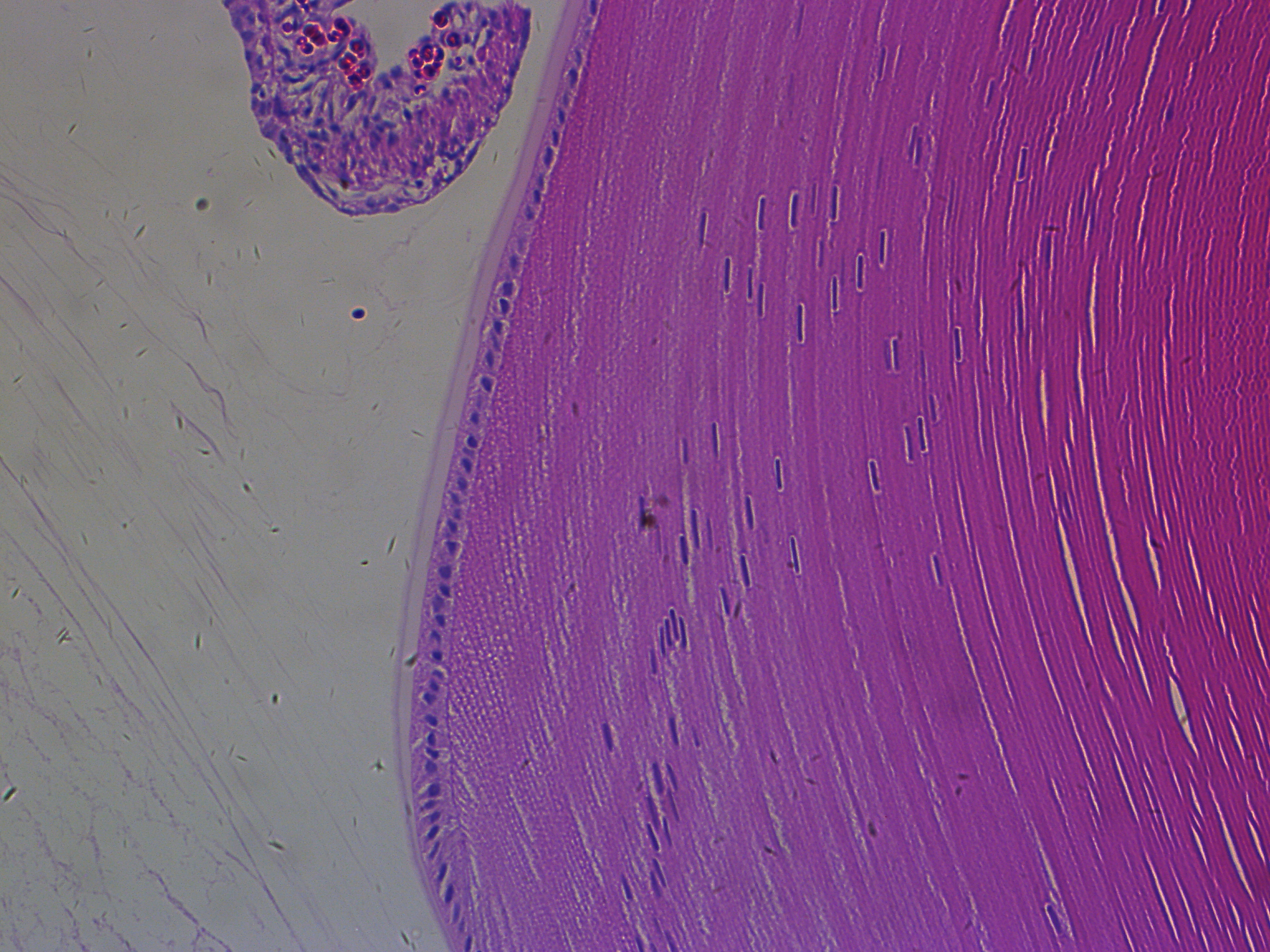

Supplement: Supplementary file 13 — Source data Fig. 7 [file 44321_2025_341_MOESM13_ESM.zip › Figure 7/7B/HE staining_Lens PM+LNP-siPAI-2.tif]

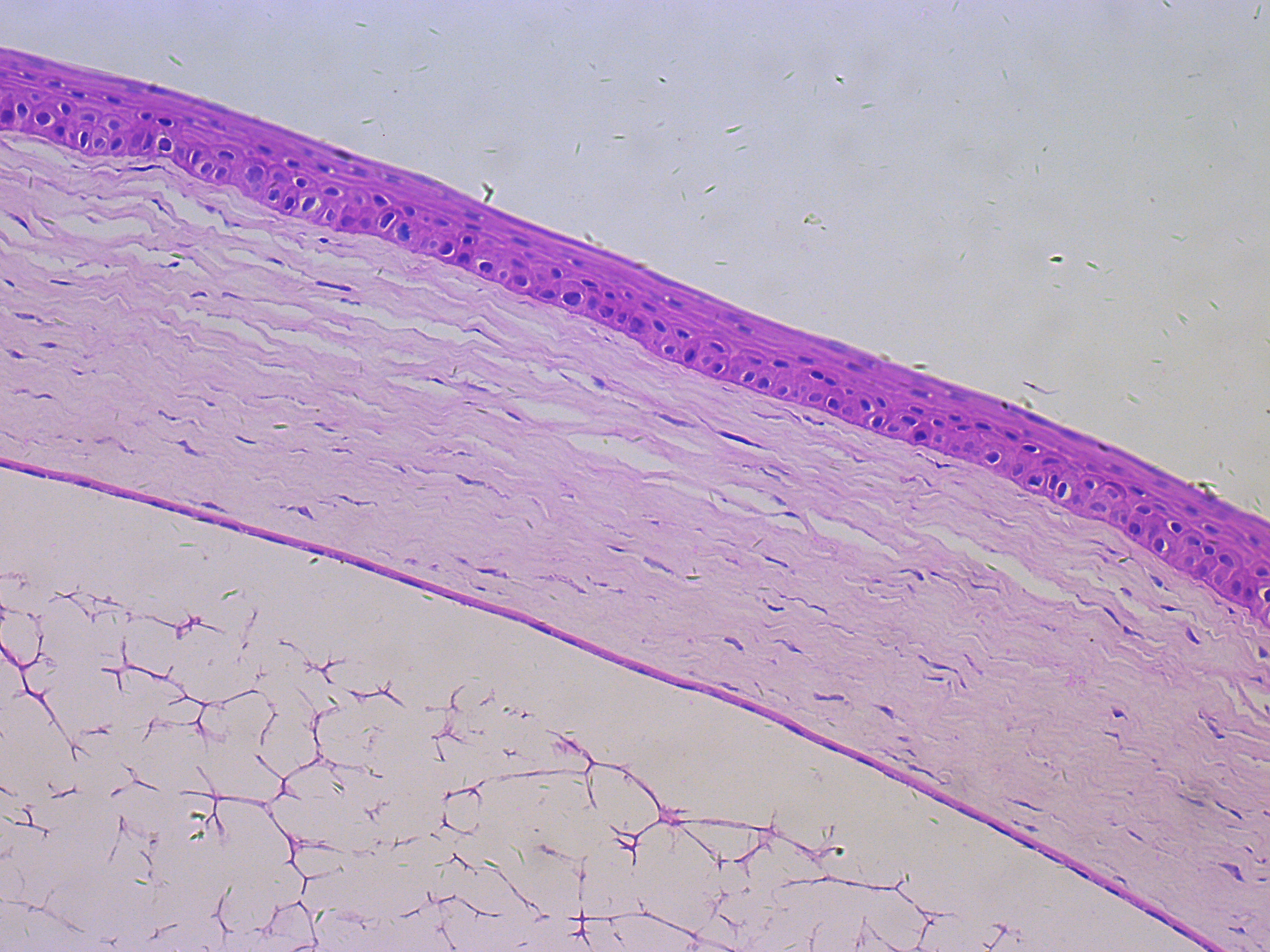

Supplement: Supplementary file 13 — Source data Fig. 7 [file 44321_2025_341_MOESM13_ESM.zip › Figure 7/7B/HE staining_Cornea PM.tif]

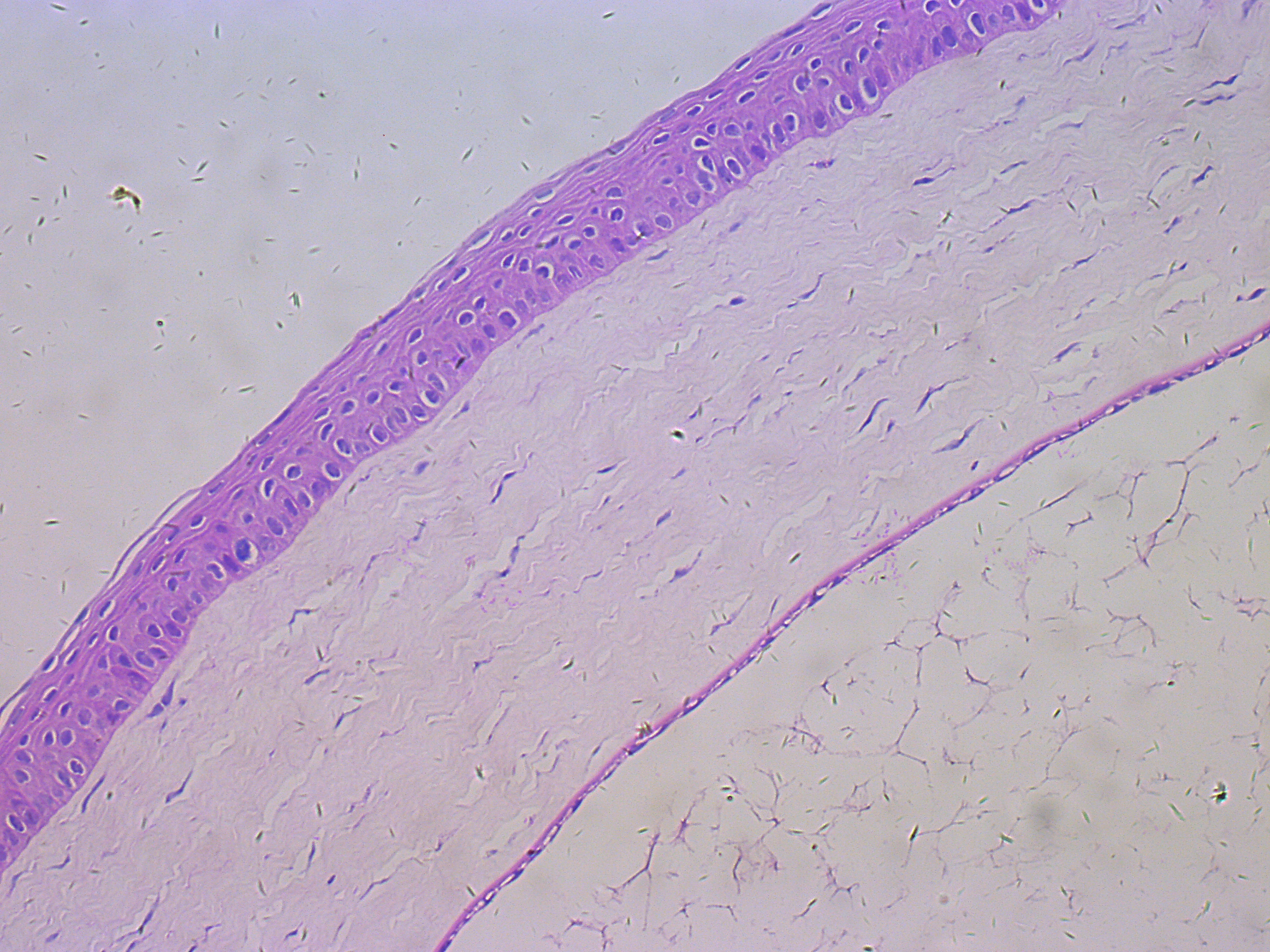

Supplement: Supplementary file 13 — Source data Fig. 7 [file 44321_2025_341_MOESM13_ESM.zip › Figure 7/7B/HE staining_Cornea PBS.tif]

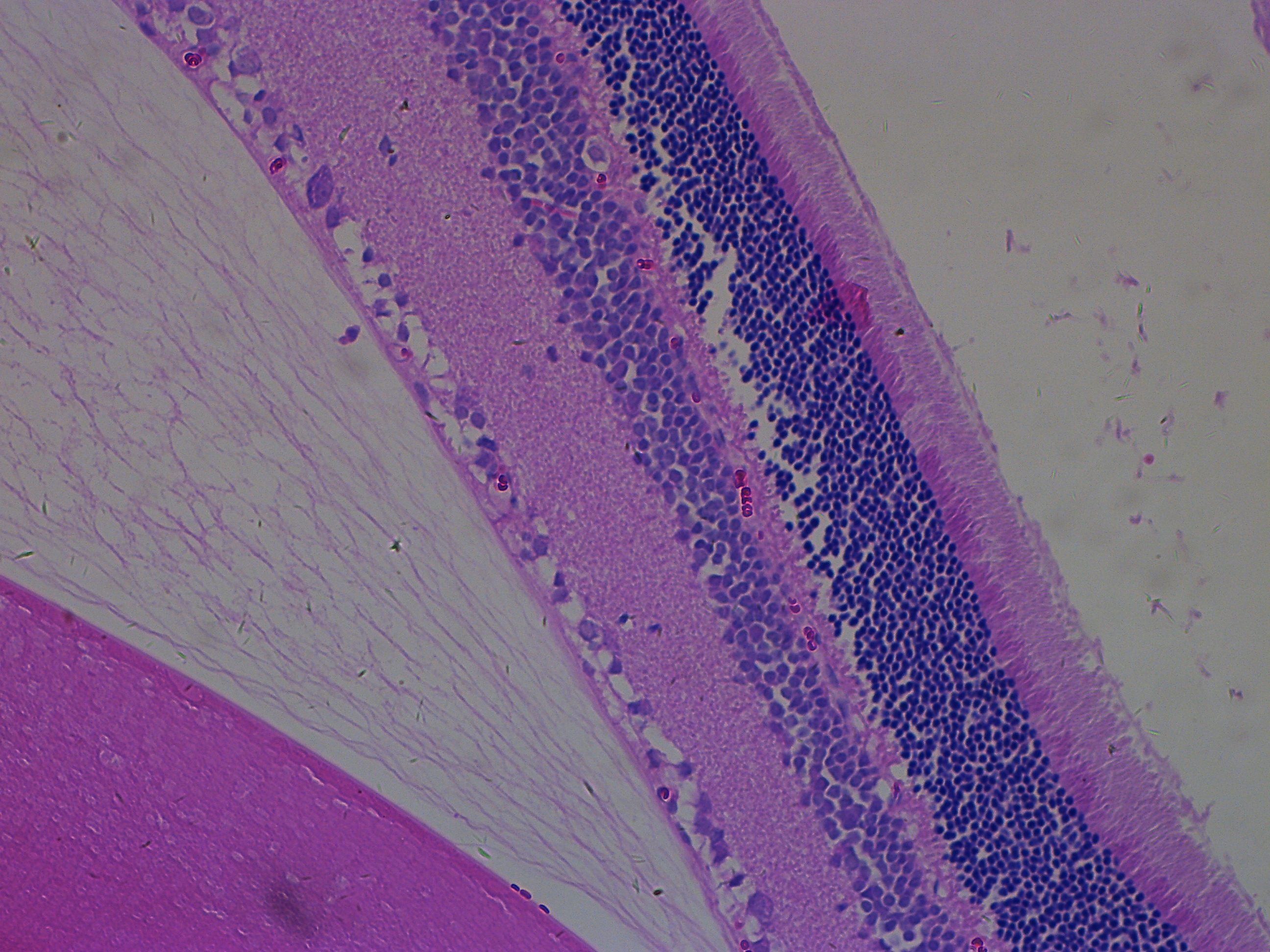

Supplement: Supplementary file 13 — Source data Fig. 7 [file 44321_2025_341_MOESM13_ESM.zip › Figure 7/7B/HE staining_Retina PBS.tif]

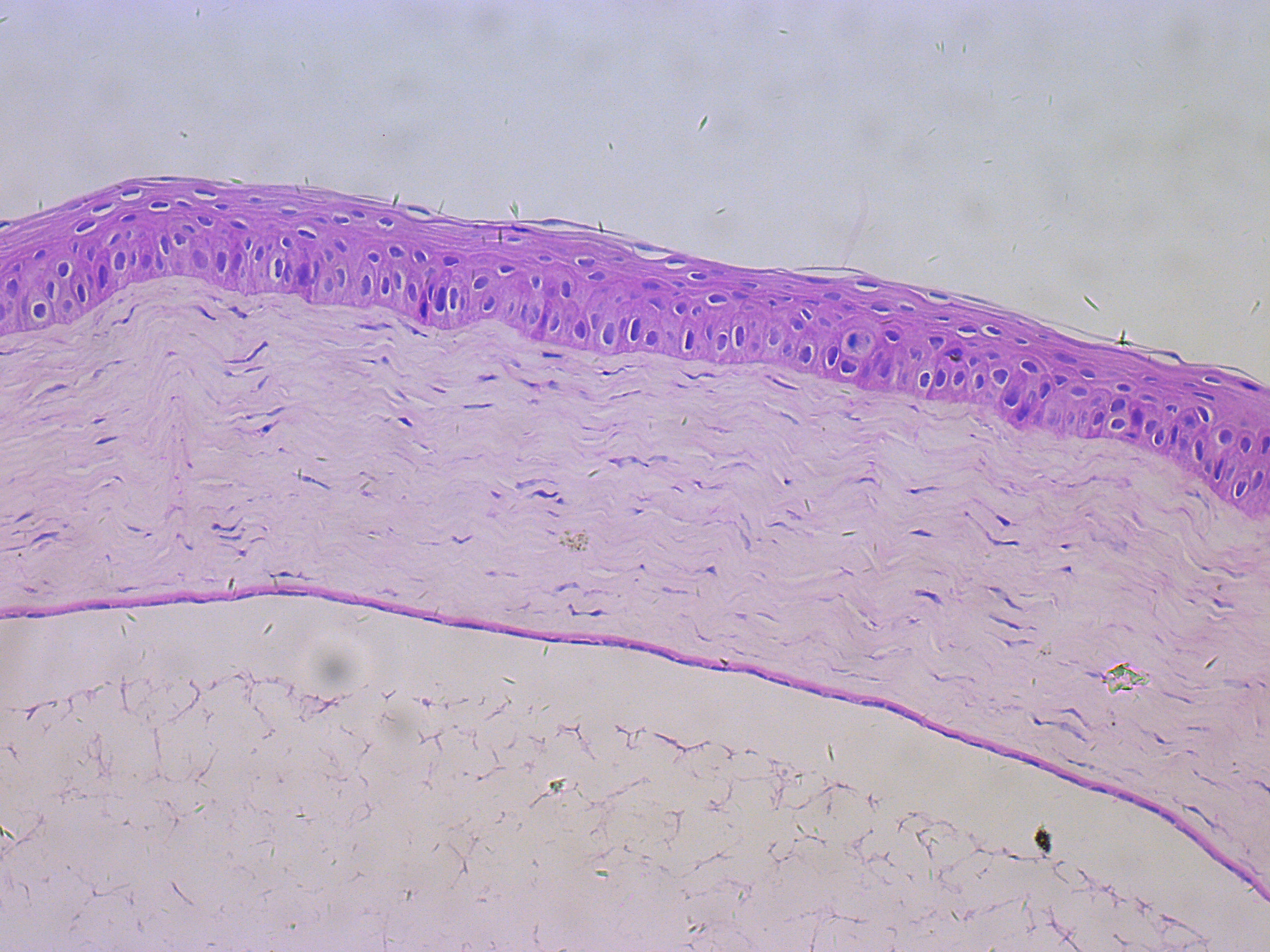

Supplement: Supplementary file 13 — Source data Fig. 7 [file 44321_2025_341_MOESM13_ESM.zip › Figure 7/7B/HE staining_Cornea PM+LNP-siRPAI-2.tif]

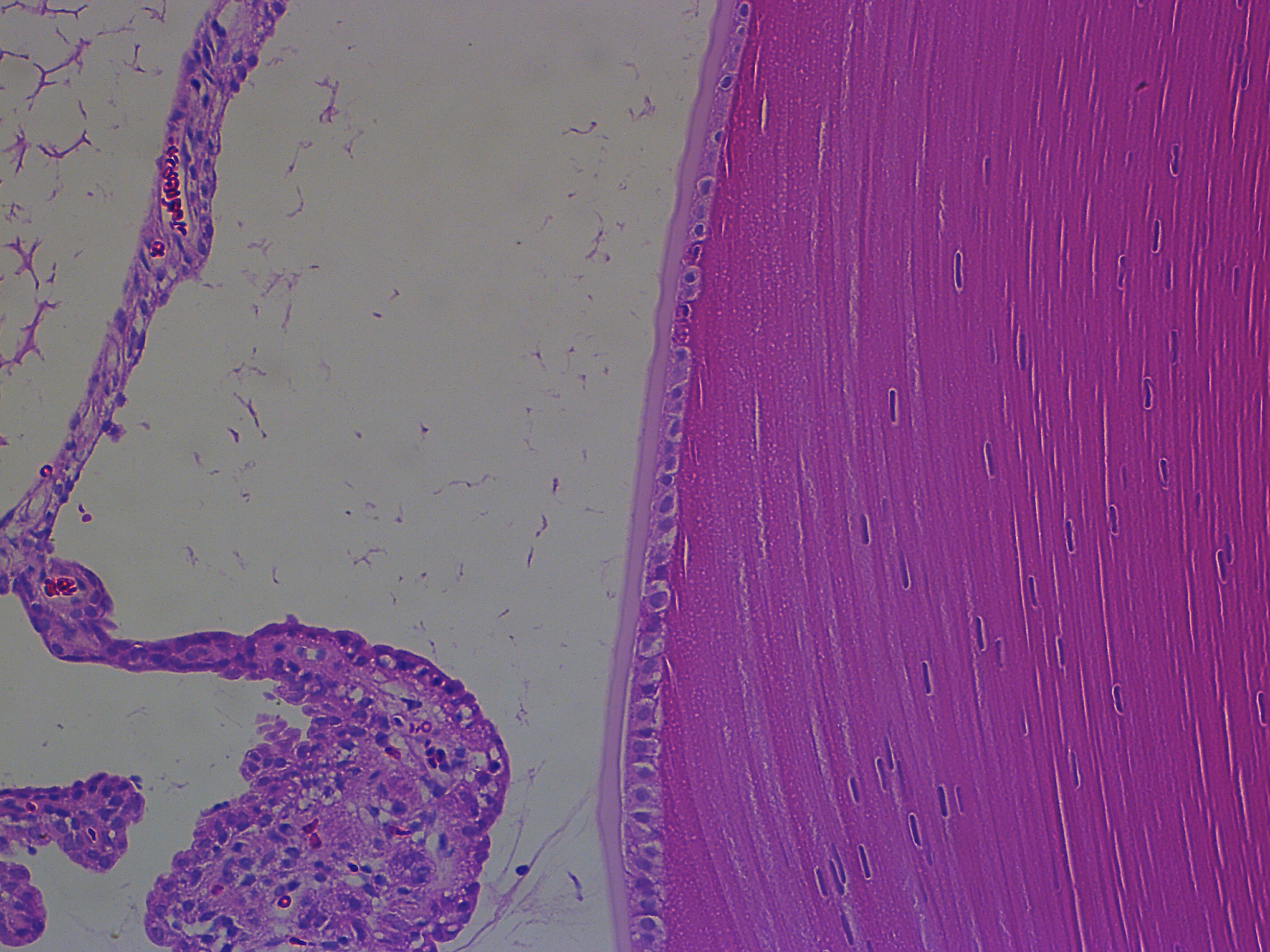

Supplement: Supplementary file 13 — Source data Fig. 7 [file 44321_2025_341_MOESM13_ESM.zip › Figure 7/7B/HE staining_Lens PM+LNP-siNC.tif]

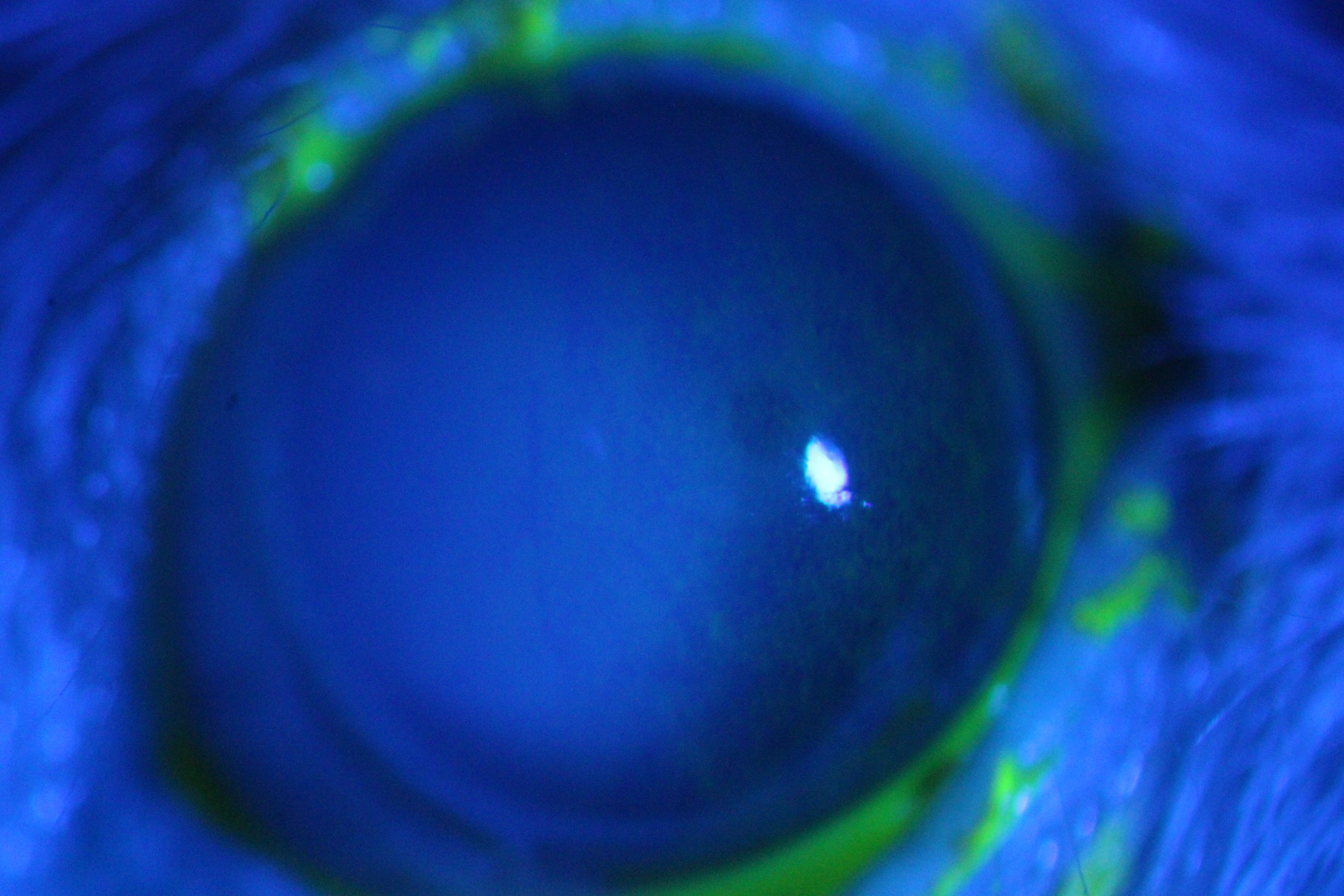

Supplement: Supplementary file 13 — Source data Fig. 7 [file 44321_2025_341_MOESM13_ESM.zip › Figure 7/7A/Rat corneal photo by slit lamp_PM+LNP.JPG]

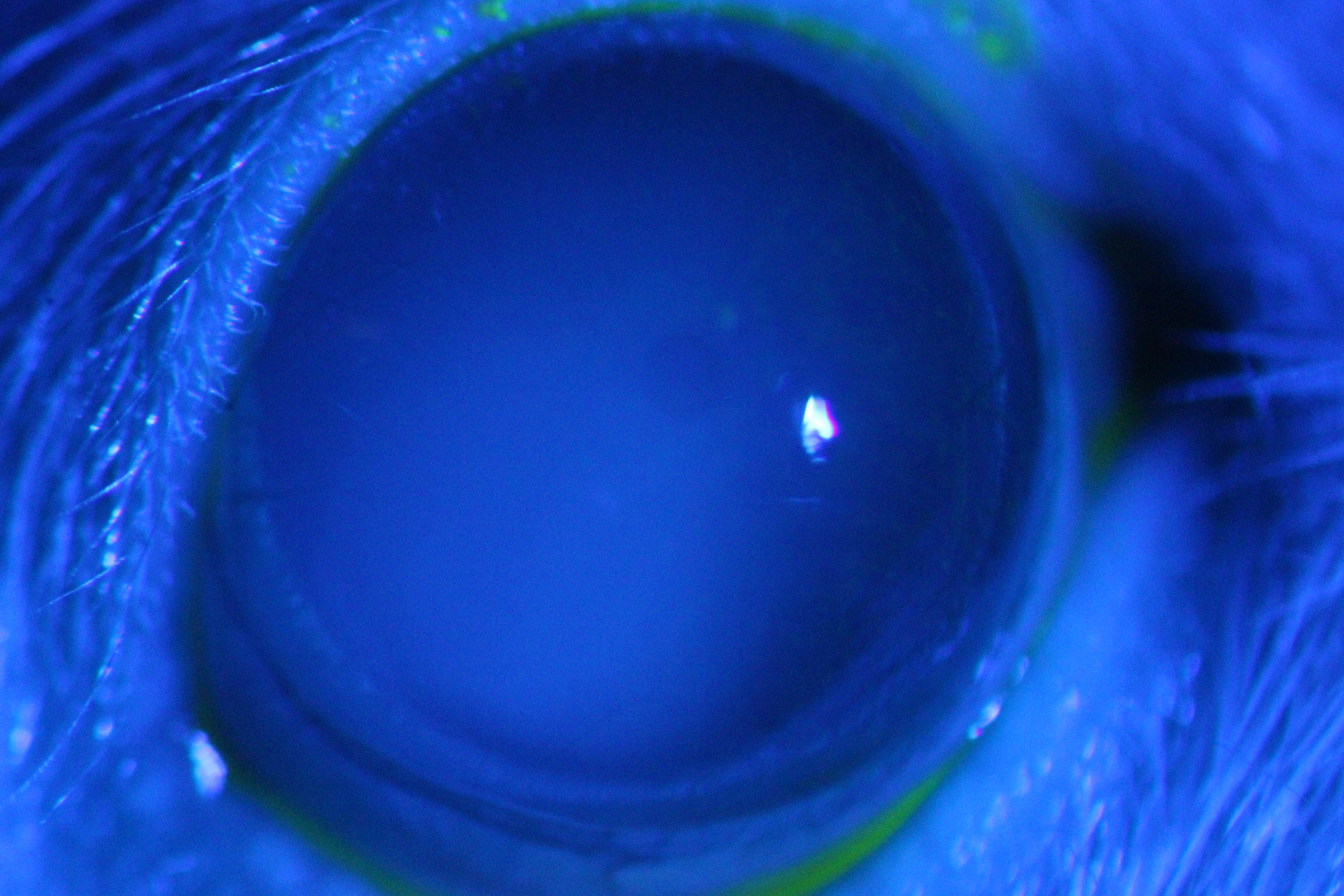

Supplement: Supplementary file 13 — Source data Fig. 7 [file 44321_2025_341_MOESM13_ESM.zip › Figure 7/7A/Rat corneal photo by slit lamp_PBS.JPG]

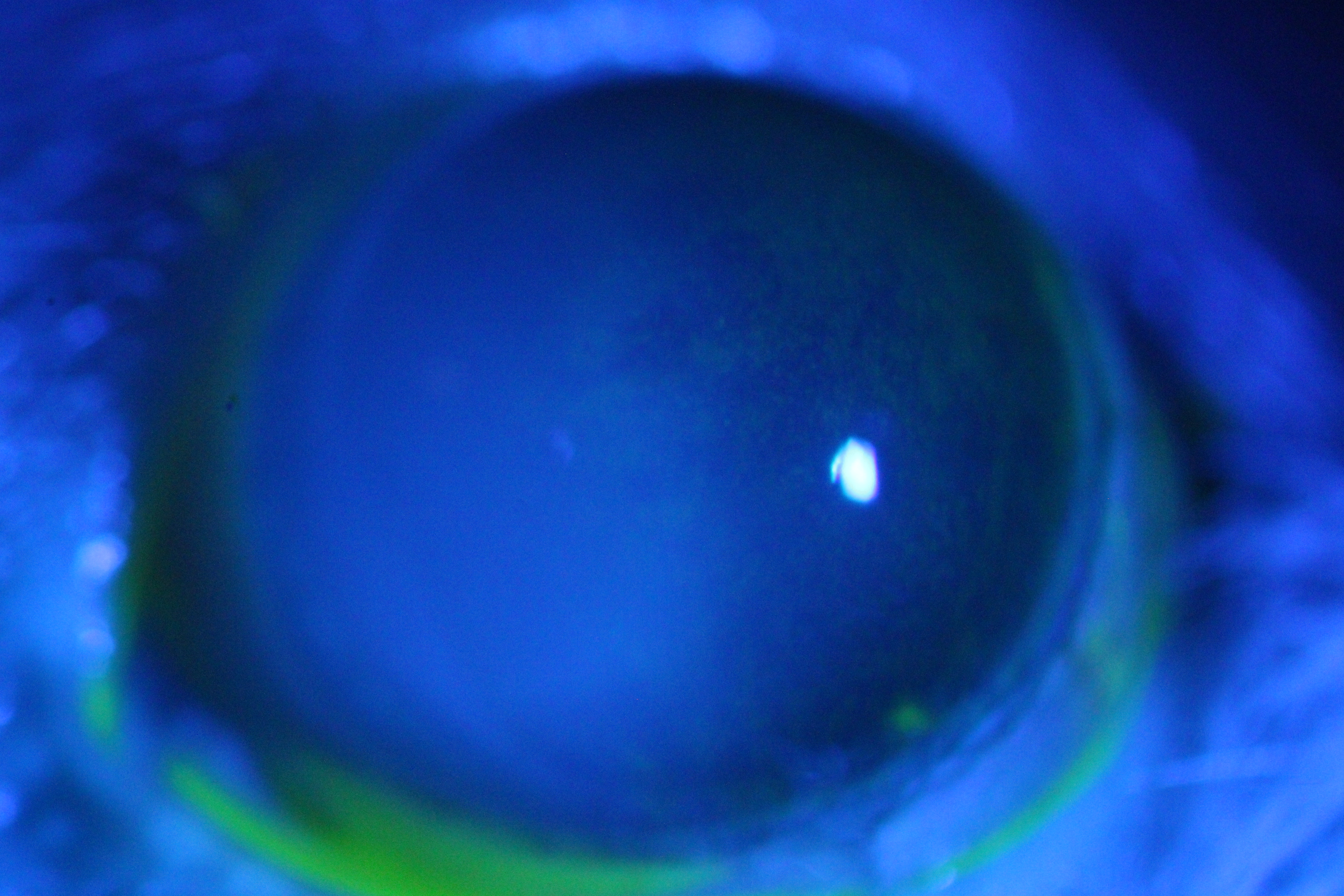

Supplement: Supplementary file 13 — Source data Fig. 7 [file 44321_2025_341_MOESM13_ESM.zip › Figure 7/7A/Rat corneal photo by slit lamp_PM+LNP-siNC.JPG]

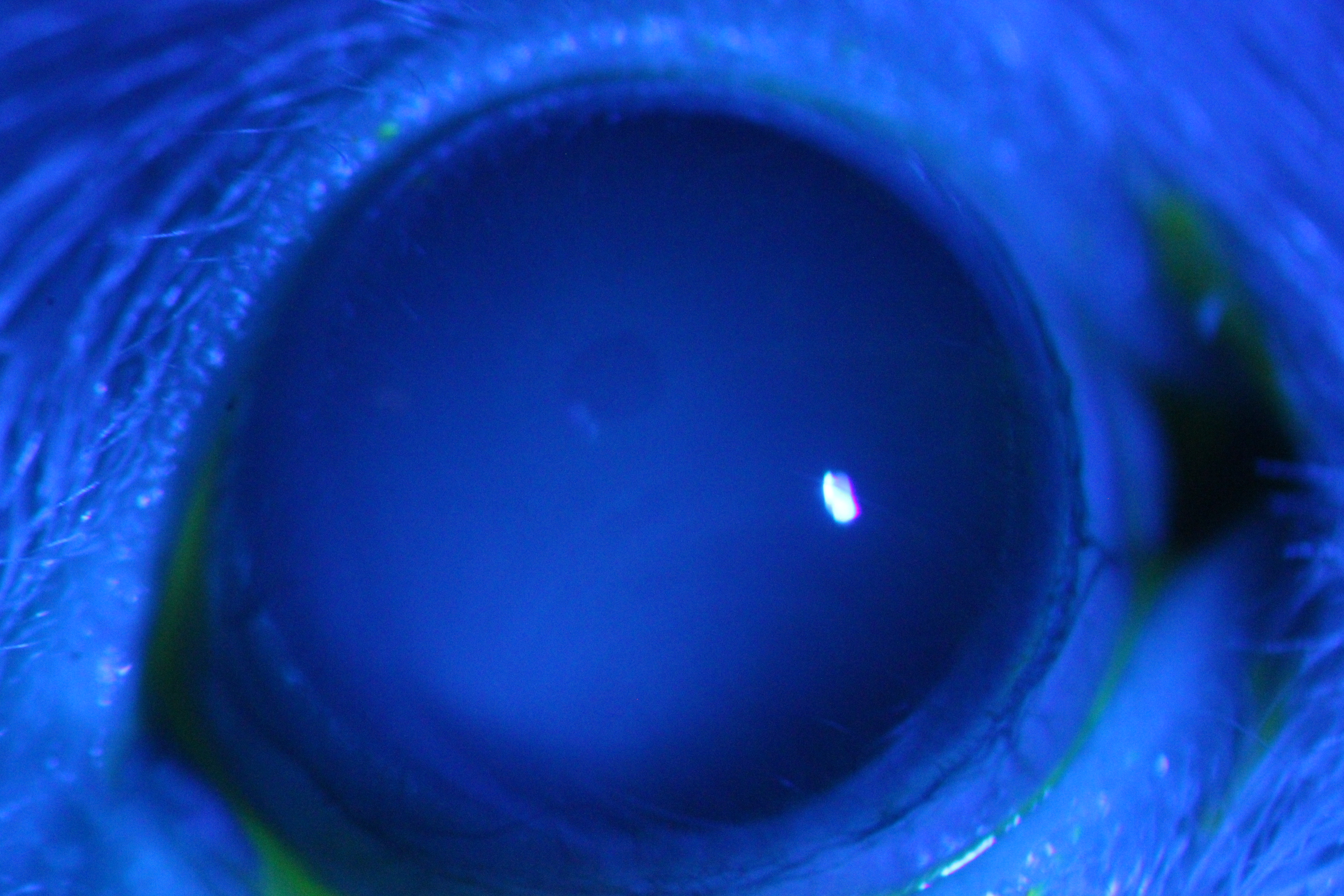

Supplement: Supplementary file 13 — Source data Fig. 7 [file 44321_2025_341_MOESM13_ESM.zip › Figure 7/7A/Rat corneal photo by slit lamp_PM+LNP-siPAI-2.JPG]

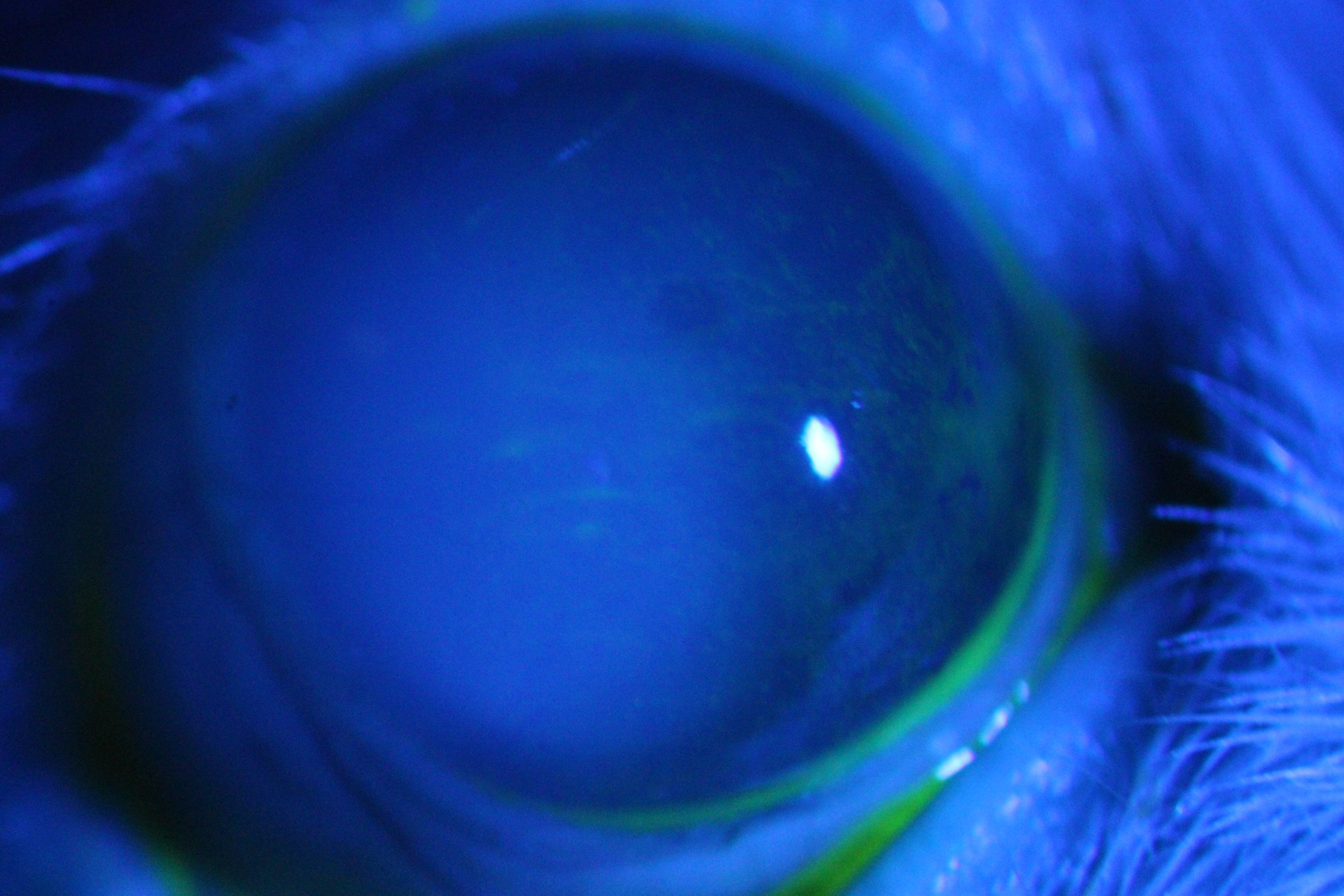

Supplement: Supplementary file 13 — Source data Fig. 7 [file 44321_2025_341_MOESM13_ESM.zip › Figure 7/7A/Rat corneal photo by slit lamp_PM.JPG]
