# Supplementary material for: Corneal biomechanical cues mediated by PAI-2: the origin of PM2.5-induced corneal disease
Source: EMBO Mol Med. 2025 Dec 1;18(1):120–50. doi: 10.1038/s44321-025-00341-0 (PMC12808792; doi:10.1038/s44321-025-00341-0)
Supplement: Supplementary file 14 — Expanded View Figures [file 44321_2025_341_MOESM14_ESM.pdf]

Expanded View Figures

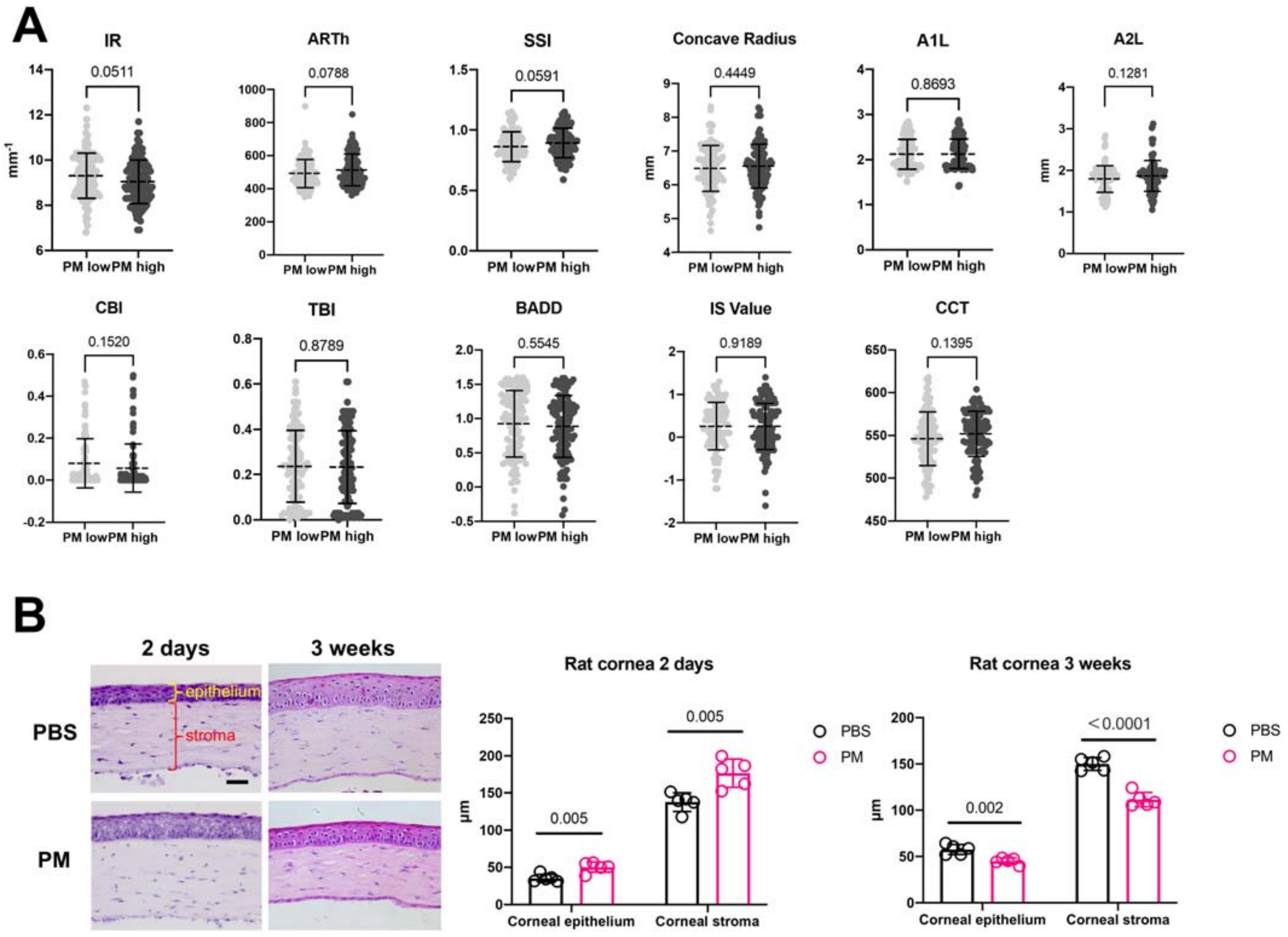

Figure EV1. Short-term PM2.5 exposure changes corneal biomechanical cues in humans and rat models.

(**A**) Corneal biomechanical parameters from Corvis ST in PM2.5 high-exposure group (PM2.5 ≥ 75 μg/cm^3, n = 113) and PM2.5 low-exposure group (PM2.5 < 35 μg/cm^3, n = 105). (**B**) H&E staining of rat cornea in two groups and corneal thickness measurement (scale bar, 50 μm). n = 5. Data in (**A**, **B**) are graphed as mean ± standard deviation with individual values shown as dots or circles. Statistical analysis was conducted using the unpaired t test in (**A**, **B**). The P values are labeled in the figure. IR integrated radius, ARTh the Ambrosio relational thickness horizontal profile, SSI stress strain index, A1L first applanation length, A2L second applanation length, CR concave radius, CBI Corvis biomechanical index, TBI tomographic biomechanical index, BADD Belin/Ambrósio Deviation, IS value inferior minus superior value, CCT central corneal thickness.

© The Author(s)

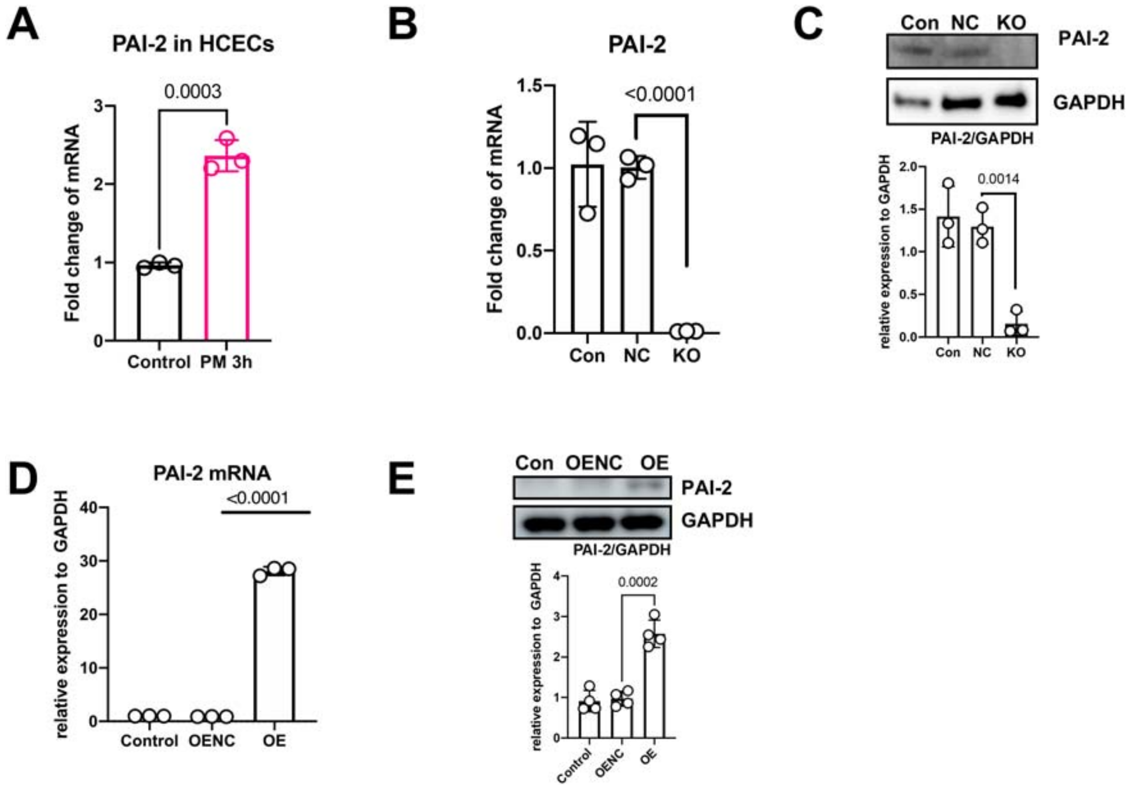

Figure EV2. The efficiency of PAI-2 knockdown and overexpression in HCECs.

(**A**) The mRNA level of PAI-2 in HCECs after short-term (3-h) PM2.5 exposure ($n = 3$ biological replicates). (**B**) The PAI-2 mRNA expression level of NC and KO ($n = 3$ biological replicates). (**C**) The PAI-2 protein expression level of NC and KO ($n = 3$ biological replicates). (**D**) The PAI-2 mRNA expression level of OENC and OE ($n = 3$ biological replicates). (**E**) The PAI-2 protein expression level of OENC and OE ($n = 3$ biological replicates). Data in (**A–E**) are graphed as mean ± standard deviation with individual values shown as circles. Statistical analysis was conducted using the unpaired *t* test in (**A–E**). The *P* values are labeled in the figure.

 © The Author(s)

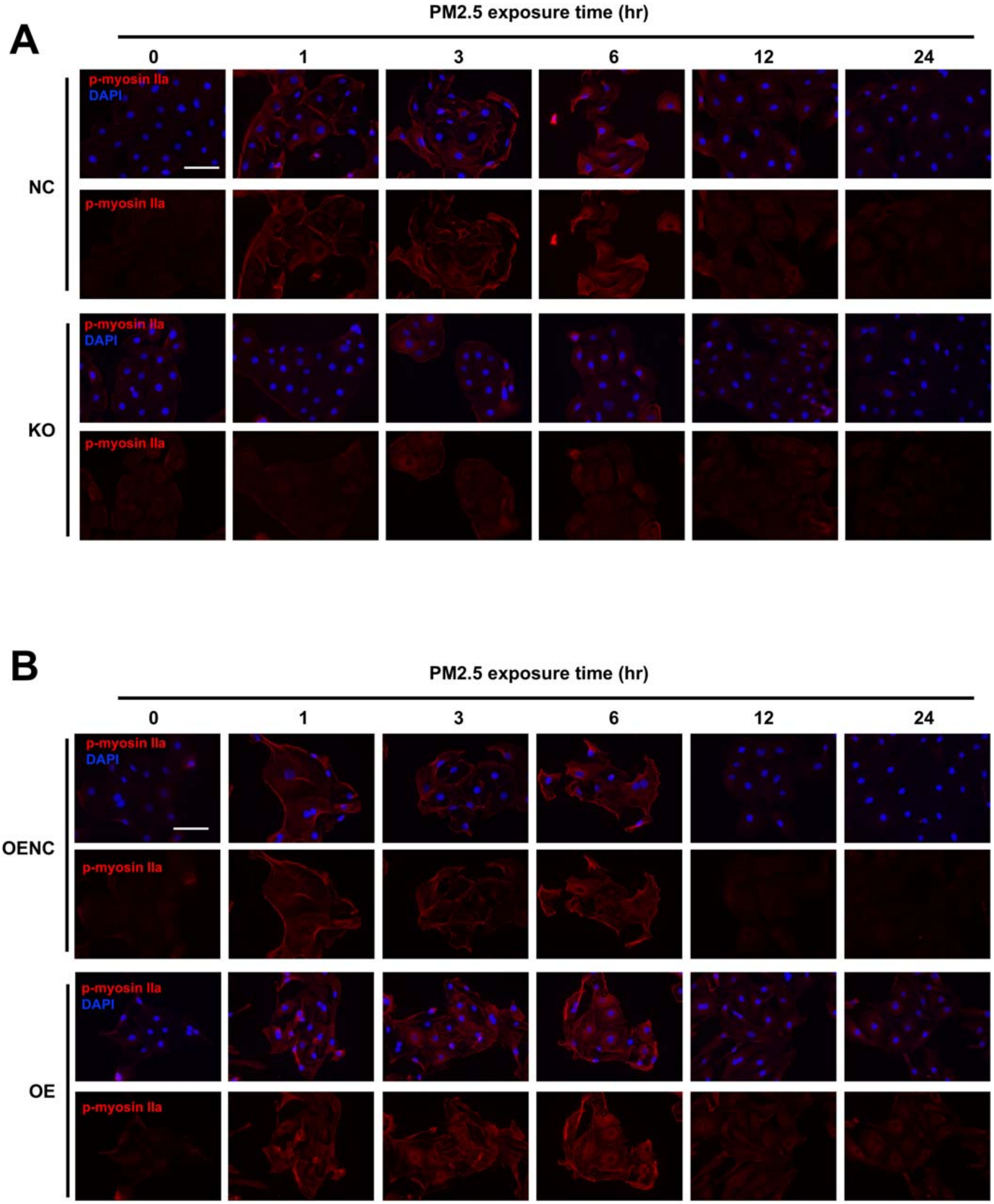
A
PM2.5 exposure time (hr)
0
1
3
6
12
24
p-myosin IIa
DAPI
NC
p-myosin IIa
p-myosin IIa
DAPI
KO
p-myosin IIa
B
PM2.5 exposure time (hr)
0
1
3
6
12
24
p-myosin IIa
DAPI
OENC
p-myosin IIa
p-myosin IIa
DAPI
OE
p-myosin IIa

Figure EV3. Phosphorylated non-muscle myosin IIa staining in NC, KO, OENC and OE with PM2.5 exposure.

(**A**) Phosphorylated myosin IIa staining in NC and KO with PM2.5 exposure (scale bar, 75 μm) ($n = 3$ biological replicates). (**B**) Phosphorylated myosin IIa staining in OENC and OE with PM2.5 exposure (scale bar, 75 μm) ($n = 3$ biological replicates).

 © The Author(s)

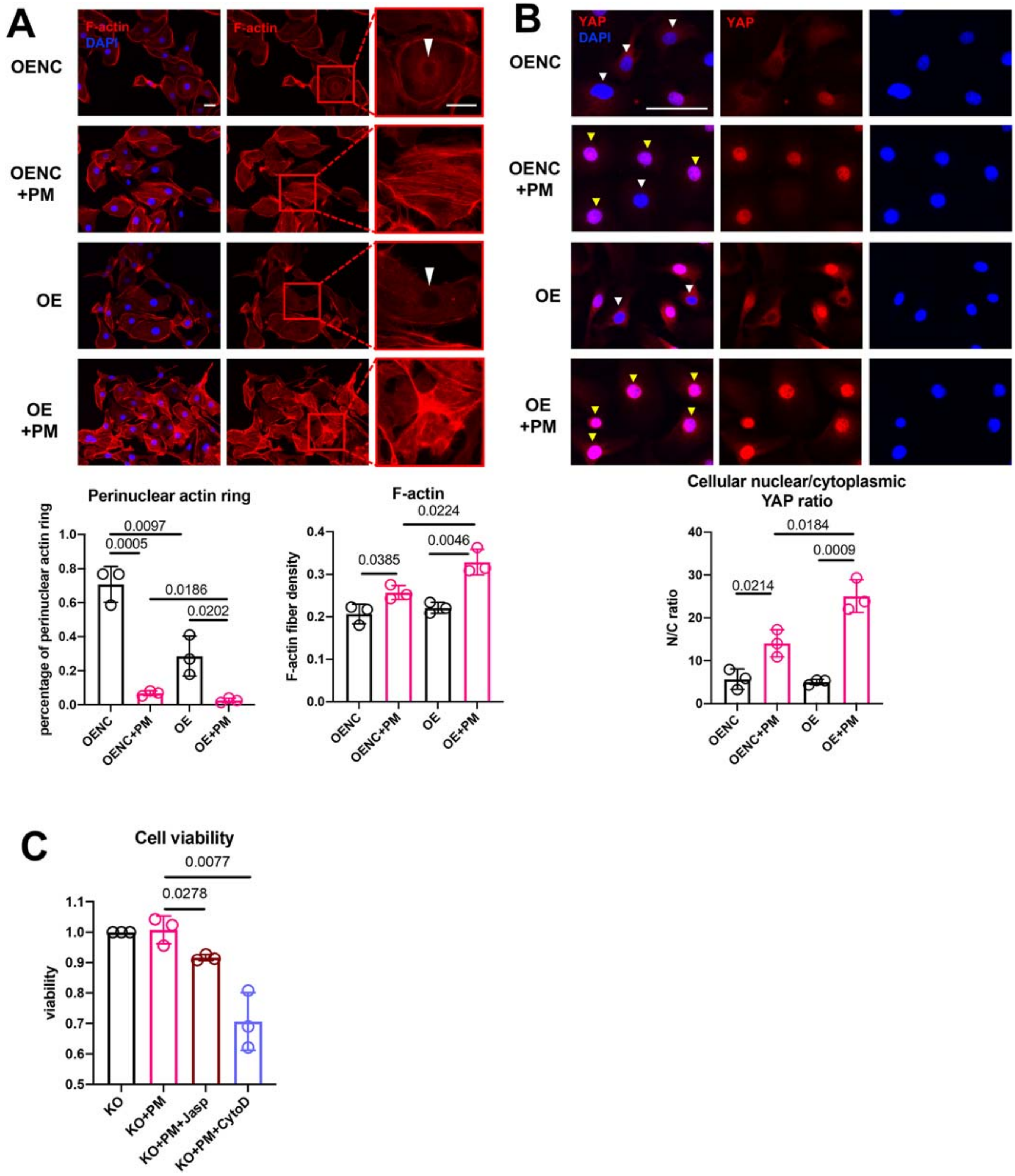
A
OENC
OENC +PM
OE
OE +PM
F-actin DAPI
F-actin
Perinuclear actin ring
percentage of perinuclear actin ring
0.0097
0.0005
0.0186
0.0202
1.0
0.8
0.6
0.4
0.2
0.0
OENC
OENC+PM
OE
OE+PM
F-actin
F-actin fiber density
0.0224
0.0046
0.0385
0.4
0.3
0.2
0.1
0.0
B
YAP DAPI
YAP
Cellular nuclear/cytoplasmic YAP ratio
N/C ratio
0.0184
0.0009
0.0214
40
30
20
10
0
C
Cell viability
viability
0.0077
0.0278
1.1
1.0
0.9
0.8
0.7
0.6
0.5
KO
KO+PM
KO+PM+Jasp
KO+PM+CytoD

Figure EV4. The staining of F-actin and YAP in OENC and OE with PM2.5 exposure.

(**A**) Staining of F-actin in OENC and OE exposed to PM2.5 for 3 h, and F-actin fiber density and PARs were measured (scale bar, 25 μm) ($n = 3$ biological replicates). The white arrow pointed to PAR. (**B**) YAP staining and YAP n/c ratio of OENC and OE with 24-h PM2.5 exposure (scale bar, 75 μm) ($n = 3$ biological replicates). The white arrow indicates cytoplasmic YAP, the yellow arrow indicates nuclear YAP. (**C**) Cellular viability after 24 h of PM2.5 treatment accompanied by PAR being interfered by jasp or cytoD ($n = 3$ biological replicates). Data in (**A–C**) are graphed as mean ± standard deviation with individual values shown as circles. Statistical analysis was conducted using the unpaired *t* test in (**A–C**). The *P* values are labeled in the figure.

© The Author(s)

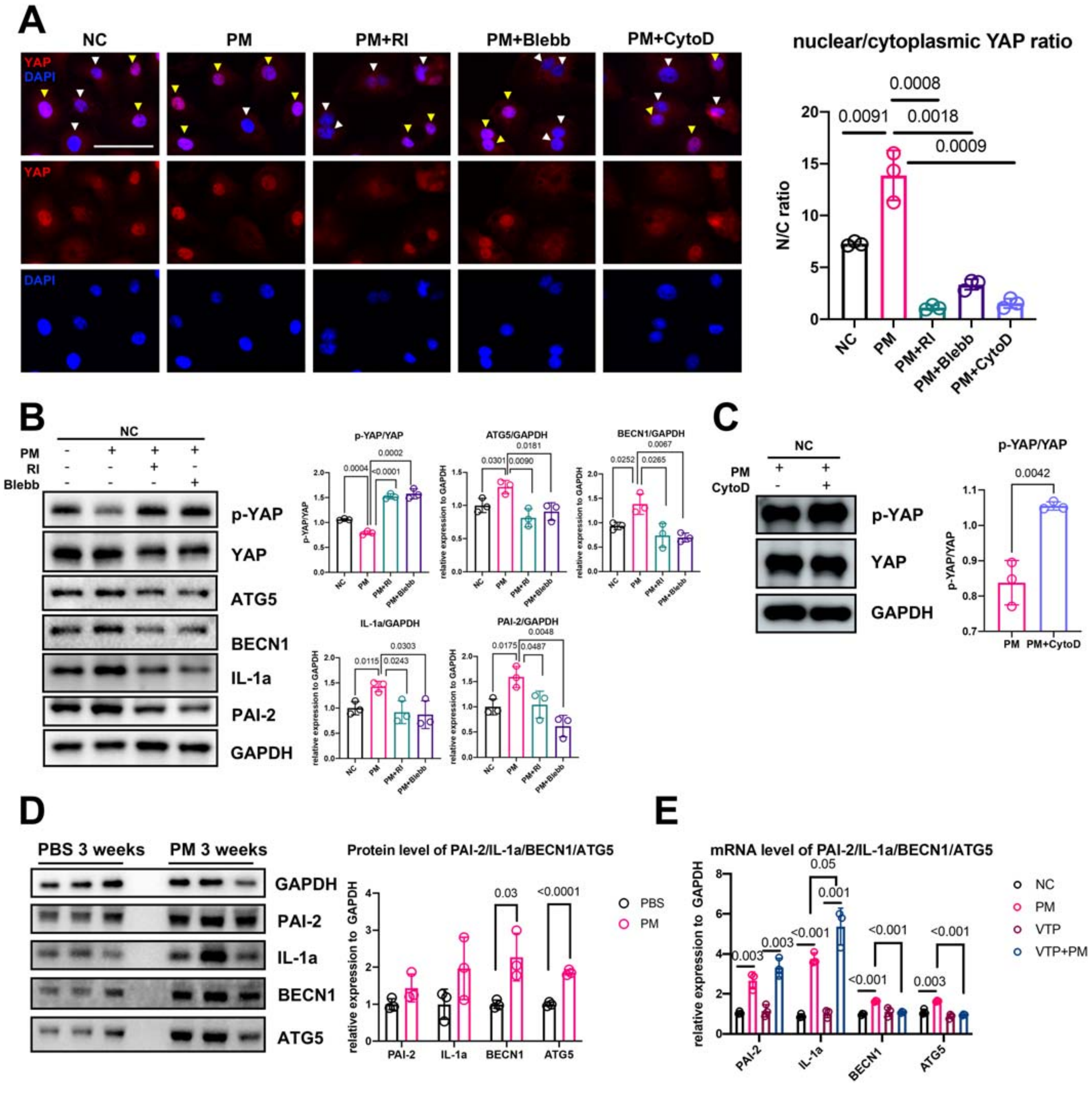

Figure EV5. YAP nuclear translocation mediates the expression of PAI-2, IL-1a, BECN1 and ATG5.

(**A**) Staining of YAP in NC after 24-h PM2.5 treatment with blebb, RI or cytoD and the nuclear/cytoplasmic YAP ratio (scale bar, 75 μm) (n = 3 biological replicates). White arrows refer to cytoplasmic YAP, and yellow arrows refer to nuclear YAP. (**B**) Protein level of p-YAP/YAP, ATG5, BECN1, IL-1a and PAI-2 in NC after 24-h PM2.5 exposure accompanied with blebb or RI (n = 3 biological replicates). (**C**) Protein expression ratio of p-YAP and YAP in NC after 24-h PM2.5 exposure accompanied with cytoD (n = 3 biological replicates). (**D**) The protein expression level of PAI-2, IL-1a, BECN1 and ATG5 in rat cornea after 3-week PM2.5 exposure (n = 3 biological replicates). (**E**) The mRNA expression level of PAI-2, IL-1a, BECN1 and ATG5 in NC after 24-h PM2.5 treatment with VTP (n = 3 biological replicates). Data in (**A–E**) are graphed as mean ± standard deviation with individual values shown as circles. Statistical analysis was conducted using the unpaired t test in (**A–E**). The P values are labeled in the figure.

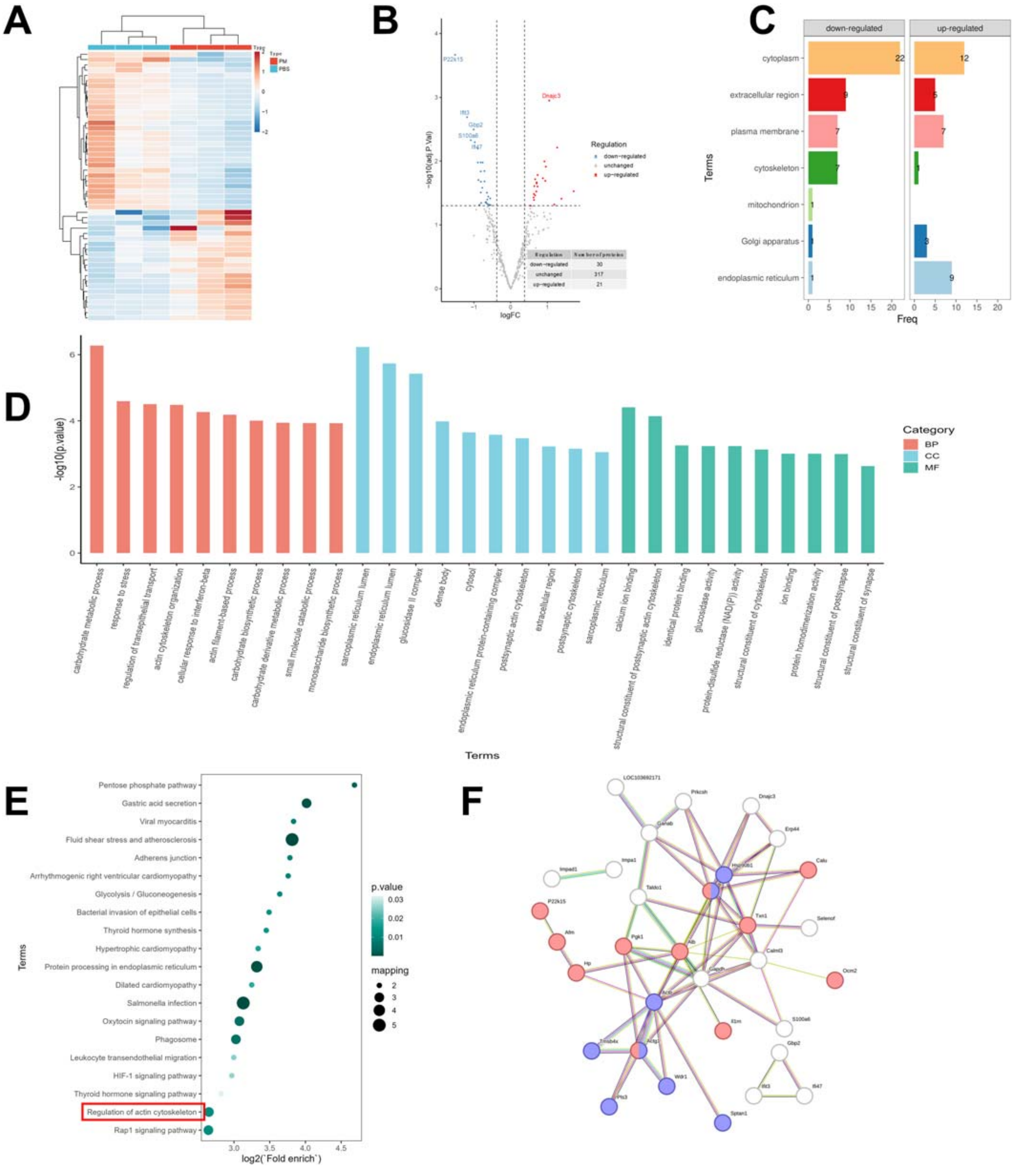

Figure EV6. Proteomic analysis of tear fluid from rats exposed to PM2.5 for 2 days.

(**A**) The heatmap showed the different expression profiling in PBS and PM2.5 groups. $n = 3$. (**B**) The volcano plot indicated the DEPs between two groups. (**C**) Subcellular localization classification of DEPs. (**D**) GO analysis of DEPs including biological process, cellular component, and molecular function. (**E**) KEGG enrichment analysis of DEPs. (**F**) PPI analysis of DEPs. The red spots refer to DEPs located in the extracellular region, the purple spots refer to DEPs related to actin organization.

 © The Author(s)

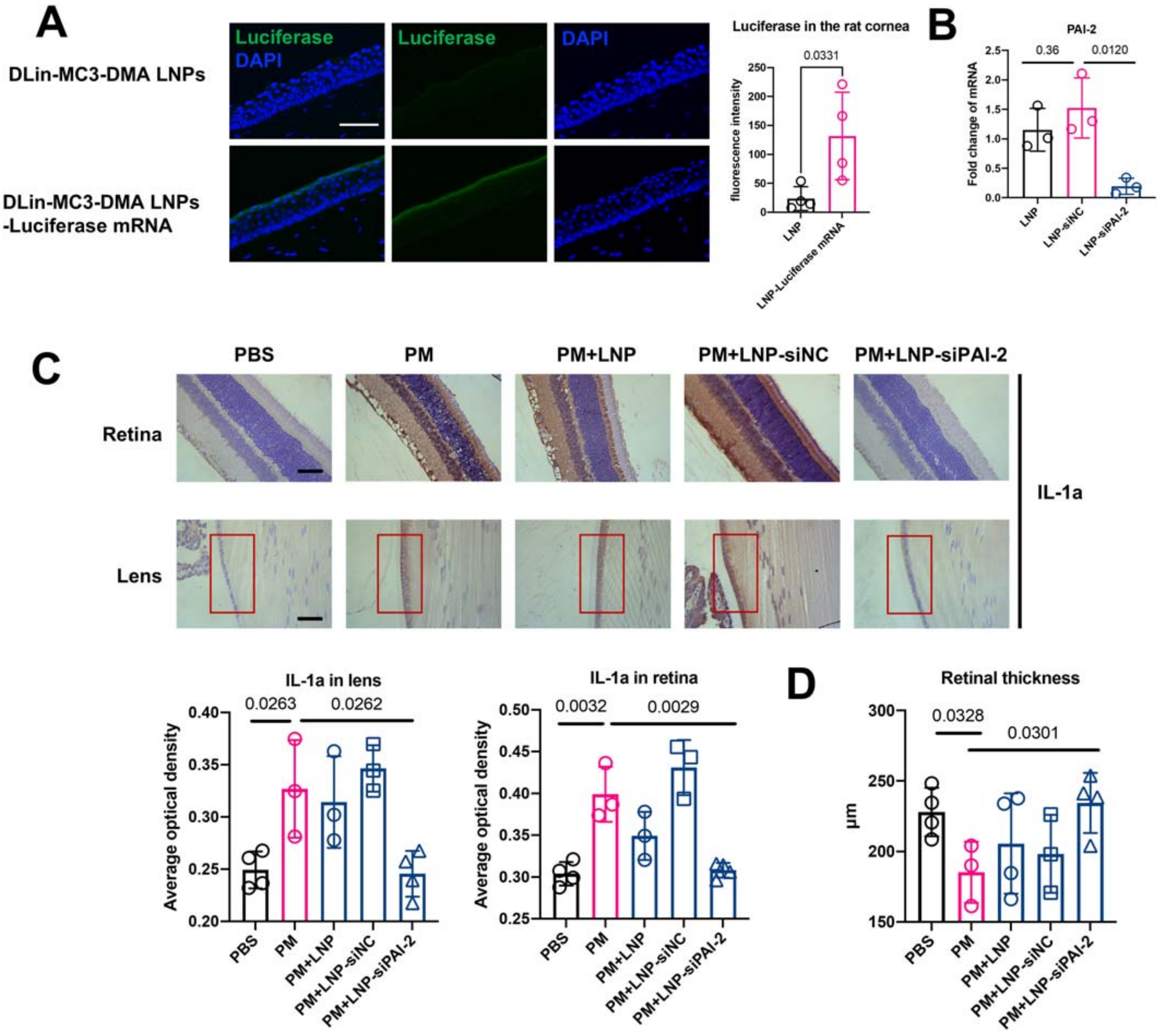

Figure EV7. LNP (siPAI-2) treatment system relieves PM2.5-induced ocular damage.

(**A**) DLin-MC3-DMA LNPs can deliver the load (luciferase mRNA) into the corneal epithelium of rats (scale bar, 75 μm). $n = 4$. (**B**) Detection of PAI-2 knockdown efficiency by LNP (siPAI-2) ($n = 3$ biological replicates). (**C**) Immunohistochemistry showed the expression level of IL-1a in rat lens and retinas of different groups (scale bar, 50 μm). $n = 4$ (PBS), 3 (PM), 3 (PM + LNP), 3 (PM + LNP-siNC), 4 (PM + LNP-siPAI-2). The red boxed refer to the rat lens epithelial cells. The average optical density of IL-1a in rat lens and retinas of different groups. (**D**) The retinal thickness based on H&E staining in Fig. 7B. $n = 4$ (PBS), 3 (PM), 4 (PM + LNP), 3 (PM + LNP-siNC), 4 (PM + LNP-siPAI-2). Data in (**A**–**D**) are graphed as mean ± standard deviation with individual values shown as circles, squares, or triangles. Statistical analysis was conducted using the unpaired t test in (**A**–**D**). The P values are labeled in the figure.
